# Label-free comparative proteomic analysis reveals distinct profiles of direct oral anticoagulants in human endothelial cells

**DOI:** 10.3389/fphar.2026.1913833

**Published:** 2026-10-28

**Authors:** Myria Pallikarou, Natalia Atzemian, George Leonidis, Konstantina Portokallidou, Georgia Ragia, Vangelis G. Manolopoulos

**Affiliations:** 1 Laboratory of Pharmacology, Medical School, Democritus University of Thrace, Dragana Campus, Alexandroupolis, Greece; 2 Individualised Medicine and Pharmacological Research Solutions (IMPReS) Center, Dragana Campus, Alexandroupolis, Greece; 3 Clinical Pharmacology Unit, Academic General Hospital of Alexandroupolis, Dragana Campus, Alexandroupolis, Greece

**Keywords:** apixaban, dabigatran, DOACs, edoxaban, HUVECs, pharmacoproteomics, proteomics, rivaroxaban

## Abstract

**Introduction:**

Direct Oral Anticoagulants (DOACs) are the first-line treatment for thromboembolism prevention in patients with atrial fibrillation. Clinical and preclinical studies suggest that, beyond their anticoagulant properties, DOACs may have additional vascular effects. However, their effects on endothelial protein-abundance profiles remain incompletely characterized. The objective of this study was to explore the protein-abundance profiles associated with exposure of human umbilical vein endothelial cells (HUVECs) to each of the four DOACs in the absence of external stimuli, using a label-free quantification (LFQ) methodology.

**Methods:**

Cultured HUVECs were exposed for 6 h to concentrations selected to approximate reported therapeutic plasma concentrations of apixaban, rivaroxaban, edoxaban, and dabigatran. All experiments were carried out in serum-free media, after a starvation period of 18 h. After protein extraction using the TRIzol Reagent, label-free quantitative proteomic analysis was performed using nanoLC-MS/MS, followed by the identification of differentially abundant proteins (DAPs) and bioinformatic enrichment analyses.

**Results:**

We identified 1,643 protein entries, of which 753 were differentially abundant. Of these, 32 showed higher abundance and 26 showed lower abundance than the vehicle control in all four DOAC comparisons. Proteins showing shared abundance changes were mapped to gene identifiers that were enriched for annotations related to autophagy and cardiac development, and cellular activation. Several proteins showing abundance changes in all three FXa-inhibitor comparisons, but not in the dabigatran comparison, were mapped to annotations related to hypoxia response, vascular permeability, and cholesterol homeostasis. However, this comparison is potentially confounded by the absence of an HCl-matched vehicle control for dabigatran. The majority of DAPs were drug specific, with distinct signatures for each DOAC. Exploratory enrichment subnetworks linked mapped gene identifiers from drug specific protein sets to ontology terms including endothelial-cell chemotaxis, sodium-ion transport, necrotic cell death, mRNA splicing, and regulation of gene expression. These cellular functions were not directly measured.

**Conclusion:**

Under the experimental conditions used, exposure to the four DOACs was associated with common and drug-specific protein-abundance patterns in HUVECs. These protein-abundance patterns provide hypothesis-generating observations for subsequent functional investigation.

## 1 Introduction

The vascular endothelium consists of the cell layer lining the blood luminal surface of vessels and plays a crucial role in providing proper hemostatic balance (Pasut et al., 2025). Endothelial dysfunction is a major constituent in the pathogenesis of thrombotic and cardiovascular diseases, including atrial fibrillation (AF), venous thromboembolism, and atherosclerosis (Sun et al., 2020; Allbritton-King and García-Cardeña, 2023; Florido and Ziats, 2024; Corban et al., 2021). Endothelial cells recognize numerous stimuli (such as oxidative stress, inflammatory signals, and hypoxia) and trigger different responses to preserve vascular homeostasis. Groten et al. (2024) showed that HUVECs represent a broad range of proteins detected across *in vitro* cultured endothelial-cell models, emphasizing their suitability for studying EC biology (Groten et al., 2024). In addition, HUVECs are used to study physiological and pathological processes associated with the cardiovascular system and to investigate molecular responses to pharmacological interventions due to their well-characterized phenotype and relevance to human vascular physiology (Böckmann and Hinz, 2020; Gimbrone and García-Cardeña, 2016; Zolotoff et al., 2022; Aird, 2012; Cai and Harrison, 2000).

Direct oral anticoagulants (DOACs) are the established first-line therapies used for the prevention and treatment of thromboembolic indications and stroke in AF. DOACs, including direct factor Xa (FXa) inhibitors (apixaban, rivaroxaban and edoxaban) and direct thrombin (FIIa) inhibitor (dabigatran), constitute a major improvement in handling thromboembolic risk, especially in AF and venous thromboembolism, with stable pharmacokinetics that avoid the difficulties of routine warfarin monitoring (Chen et al., 2020; Steffel et al., 2021; Riva and Ageno, 2021; Carnicelli et al., 2022; Ruff et al., 2014). Yet, their effects on endothelial cells apart from anticoagulation, including reported associations with inflammatory markers and endothelial-cell responses in experimental models, are not fully understood and mandate further study (Atzemian et al., 2023; Jannati et al., 2024). Apixaban has demonstrated pleiotropic endothelial effects including anti-inflammatory and anti-oxidant actions in preclinical models (Atzemian et al., 2023). These include modulation of cytokine expression, reduction in reactive oxygen species, and protection of endothelial integrity, consistent with broader non-coagulant DOAC actions (Torramade-Moix et al., 2021; Simmers et al., 2016). In HUVECs, rivaroxaban treatment has been linked to dysregulation of inflammatory and adhesion molecules, including VCAM-1, ICAM-1, and IL-8, which has been interpreted in previous studies as being consistent with changes in inflammatory, proliferative, or angiogenesis-related responses in endothelial cells (Atzemian et al., 2023; Papadaki et al., 2021; Kitasato et al., 2024). Previous studies have reported rivaroxaban-associated changes in markers related to endothelial metabolism and cellular stress. Treatment with rivaroxaban altered CYP4F2 expression and reduced oxidative stress markers, associating with metabolic and redox regulation (Ciapiene et al., 2021). Edoxaban shares a similar pharmacologic profile to apixaban and rivaroxaban. Experimental studies have reported that edoxaban exposure is associated with reducing inflammatory and oxidative pathways, decreasing endothelial permeability, and exerting anti-atherosclerotic effects (Atzemian et al., 2023; Goette et al., 2023; Almengló et al., 2020). Finally, the direct thrombin inhibitor dabigatran has been reported to reduce inflammatory signaling and angiogenic responses in cultured endothelial cell models (Atzemian et al., 2023), and to protect hepatic sinusoidal endothelial cells and reduce liver injury in a murine hepatic ischemia-reperfusion model, associated with enhanced thrombomodulin expression and reduced HMGB-1 release (Noguchi et al., 2021).

Despite their established mode of action, there is a lack of information on specific proteins whose abundance may change following DOAC exposure. In addition, little is known about potential differences between individual DOACs at the cellular level. Liquid chromatography–tandem mass spectrometry (LC–MS/MS) based label-free quantification (LFQ) offers a powerful approach for identifying and characterizing differentially abundant proteins (DAPs) in response to pharmacological treatments and identifying protein sets annotated to signaling pathways and biological processes that may warrant subsequent functional investigation in various cell types, including endothelial cells (Mann et al., 2013; Cox et al., 2014; Liu et al., 2013; Ren et al., 2020). In addition, LFQ proteomics has become a reliable and widely used technique for comparative proteomics, allowing accurate relative quantification of thousands of proteins without the need for isotopic labeling (Aebersold and Mann, 2016). However, discovery-scale proteomic profiling of DOAC-treated endothelial cells remains insufficiently investigated, since there are no studies reporting LC-MS/MS-based proteomic analysis of *in vitro* endothelial cell models specifically treated with apixaban, rivaroxaban, edoxaban, dabigatran, alone or in combination.

In this study, we used LFQ-based proteomics to perform discovery-scale proteomic profiling of the four DOACs in HUVECs. By identifying DAPs and enriched pathway annotations, this work aims to provide exploratory insight into the endothelial responses to each DOAC therapy and to expand our understanding of their potential endothelial responses that may warrant functional investigation. In addition, comparison of the protein-abundance patterns observed for the four DOAC treatment conditions was used to identify differences between them at protein level.

## 2 Methodology

### 2.1 Cell culture and drug treatment

Human umbilical vein endothelial cells (HUVECs, pooled donor, PromoCell C-12203) were cultured in Endothelial Cell Growth Medium 2 (PromoCell C-22211) supplemented with growth factors, 10% FBS (Sigma-Aldrich, cat. no. F7524) and 1% pen/strep (PAN BIOTECH, cat. no. P06-07100). Cells were incubated at 37 °C in a humidified 5% CO_2_ atmosphere and the medium was renewed every 2 days. For the experiments, cells were used at passage 5 according to the manufacturer’s instructions.

Cells were seeded in 12-well plates (1.8 × 10^5^ cells/well). When 80% confluence was reached, cells were serum-starved in serum-free medium for 18 h to reduce serum-associated variability before drug exposure. Stock solutions of the DOACs apixaban, rivaroxaban, and edoxaban (Sigma-Aldrich, cat. nos. SML3285, SML2844, SML3263) were prepared in DMSO 0.005% v/v, while dabigatran stock solutions (Sigma-Aldrich, cat. no. SML2370) were prepared in 0.01 M HCl. HUVECs were treated for 6 h with the following concentrations of the four drugs: 60 ng/mL apixaban (130.58 nM), 100 ng/mL rivaroxaban (229.42 nM), 60 ng/mL edoxaban tosylate (81.27 nM), and 100 ng/mL dabigatran (212.08 nM). Selected concentrations were chosen with reference to previously reported plasma concentrations for standard dosing regimens of each drug (Atzemian et al., 2023; Frost et al., 2013; Almalbis et al., 2025; Parasrampuria and Truitt, 2016; Ng et al., 2022). For apixaban, 60 ng/mL falls near the lower end of the peak plasma concentration range observed with standard 5 mg twice-daily dosing (approximately 63–299 ng/mL) (Almalbis et al., 2025). For rivaroxaban, 100 ng/mL falls near the lower end of the peak plasma concentration range observed with standard 20 mg once-daily dosing (approximately 98–367 ng/mL) (Almalbis et al., 2025). For edoxaban, 60 ng/mL edoxaban tosylate corresponds to the unbound (free) fraction (fu = 0.45) calculated from a clinically relevant total plasma concentration and falls between reported trough and peak plasma concentrations across the dosing interval for standard patient exposure levels (trough range approximately 11–23 ng/mL; peak range approximately 150–309 ng/mL) validated in large-scale clinical trials (Parasrampuria and Truitt, 2016; Giugliano et al., 2013). For dabigatran, 100 ng/mL falls below the pooled peak plasma concentration reported across observational studies of standard 150 mg twice-daily dosing (133 ng/mL, 95% CI: 113–154) (Ng et al., 2022). The 6 h exposure period was selected on the basis of previous *in vitro* studies using comparable DOAC exposures (Torramade-Moix et al., 2021; Almengló et al., 2020; Ellinghaus et al., 2016). The DMSO vehicle control was prepared at DMSO concentration (0.005%). No separate HCl-matched vehicle control was included for dabigatran. Because no HCl-matched vehicle control was included, the dabigatran-versus-control comparison cannot unambiguously distinguish a dabigatran-associated effect from a solvent-associated effect. Three independent HUVEC experiments were performed on different days. In addition, all three experiments derive from a single pooled-donor HUVEC stock (one thaw, passage 5 throughout), so the three experiments are independent in timing and handling. Within each experiment, cells were seeded into three separate wells per treatment condition, yielding nine wells per condition across the three experiments. Treatment assignment and sample processing order were randomized to wells. Each biological sample was subjected to three repeated LC–MS/MS injections. Technical injections were combined within each biological sample before statistical analysis; therefore, differential abundance testing was performed using the biological sample as the independent experimental unit (n = 9 per condition).

MTT assay was employed to measure cellular metabolic activity as an indicator of cellular metabolic activity (CAYMAN cat. no. 10009365) following manufacturer’s instructions. Briefly, HUVECs were seeded in 96-well culture plates (10,000 cells/well/100 μL) in complete EGM-2 medium. After 24 h, complete medium was replaced with serum-free medium for 18 h. Then, cells were treated with the same concentrations of DOACs, and vehicle control for 6 h. MTT reagent (10 μL) was added to each well, followed by 4 h of incubation at 37 °C. Formazan crystals were dissolved by adding 100 μL of crystal dissolving solution per well, followed by measurement of the absorbance at 570 nm by a microplate reader. Viability values were normalized to vehicle-treated control cells, which were assigned a value of 100%. Data are expressed as mean ± SD. Statistical analysis was performed using one-way ANOVA followed by Dunnett’s multiple comparisons test.

### 2.2 Protein extraction and quantification

Cells were collected and subjected to phenol-based extraction using TRIzol reagent (Invitrogen/Thermo Fisher Scientific 15596026), allowing sequential fractionation of RNA, DNA, and proteins. Briefly, HUVEC samples were homogenized by repeated pipetting after the addition of 1 mL TRIzol reagent. Samples were incubated at room temperature for 5 min, followed by 200 μL chloroform addition and centrifugation at 12,000 × g for 15 min at 4 °C to achieve phase separation. The upper aqueous phase containing RNA was carefully removed. DNA was precipitated from the organic phase by adding 210 μL of 100% ethanol and removed following centrifugation at 12,000 × g for 15 min at 4 °C. Proteins were then precipitated from the remaining organic phase by adding 100% isopropanol, followed by incubation at 4 °C for 10 min and centrifugation at 12,000 × g for 10 min at 4 °C. The resulting protein pellets were washed three times with 100 μL of 0.3 M guanidine hydrochloride in 95% ethanol, with centrifugation at 7,500 × g for 5 min at 4 °C after each wash. Finally, the pellets were washed with 100% ethanol, air-dried for 5–10 min while avoiding excessive drying, and resuspended in 200 μL of 50 mM ammonium bicarbonate buffer. Samples were heated at 50 °C for 1 h to improve protein solubilization. Protein concentration was subsequently determined by UV–Vis spectrophotometry at 280 nm. Ammonium bicarbonate buffer exhibits a minimal background absorbance (A_max≤0.020), ensuring high spectrophotometric sensitivity (https://www.chemicalbook.com/ChemicalProductProperty_EN_CB9354407.htm). Protein yield was 75.47 ± 37.69 μg per well (mean ± SD).

### 2.3 Sample preparation for mass spectrometry

Proteins (50 μg per sample) were reduced with 10 mM dithiothreitol (DTT) at 60 °C for 1 h, alkylated with 20 mM iodoacetamide (IAA) at 37 °C for 1 h in the dark. Samples were digested overnight at 37 °C using a sequencing-grade modified trypsin (Promega V5111; stock solution 0.7 μg/μL in ammonium bicarbonate buffer). The first addition comprised 1.43 μL of trypsin stock, corresponding to approximately 1 μg trypsin and an initial trypsin-to-protein ratio of 1:50 (w/w). A second aliquot of 0.48 μL of the same stock, corresponding to approximately 0.34 μg trypsin, was subsequently added, followed by incubation at 37 °C for a further 3 h. The cumulative trypsin-to-protein ratio was approximately 1:37.5 (w/w). Digestion was stopped by adding trifluoroacetic acid (TFA) to a final concentration of 1%. Digestion completeness was not independently assessed by an additional biochemical assay; the missed-cleavage rate among identified peptides was inspected in Proteome Discoverer: target PSMs (n = 4,877) showed a mean of 0.39 missed cleavages (median 0, range 0–2), and target peptide groups (n = 999) showed a mean of 0.55 missed cleavages (median 0), consistent with efficient digestion. ZipTip® C18 pipette tips (bed volume 0.6 μL, tip volume 10 μL, Millipore, ZTC18S) were used for desalting and concentrating peptide samples prior to LC-MS/MS analysis. The protocol involved wetting, equilibration, binding/washing, and elution steps performed with a 10 μL pipettor setting to manage resin back pressure. Peptides were eluted with 1% TFA in 50% ACN.

### 2.4 Nano LC-MS/MS analysis

Samples were analyzed by liquid chromatography–tandem mass spectrometry (LC–MS/MS) using an Ultimate 3000 RSLCnano system coupled to an Exactive Plus Orbitrap mass spectrometer (Thermo Fisher). Peptides were separated on an Easy-Spray PepMap RSLC C18 column (Thermo Fisher Scientific) at a flow rate of 0.300 μL/min and a column temperature of 25 °C. Solvent A consisted of 0.1% formic acid in ultrapure water, while solvent B consisted of 0.1% formic acid in acetonitrile. The initial mobile phase composition was 95% A and 5% B. Peptides were eluted using an 80 min gradient, increasing solvent B from 5% to 95% between 2 and 60 min, followed by column re-equilibration to the initial conditions for 20 min. Mass spectrometric data were acquired in positive-ion mode using a full MS scan followed by data-dependent triggering of all-ion fragmentation (full MS/dd-AIF, TopN) (Geiger et al., 2010). Fragmentation was performed without precursor-specific quadrupole isolation; therefore, a precursor isolation-window setting was not applicable. Full MS scans were acquired over an m/z range of 350–2000 at a resolution setting of 70,000, with an automatic gain control (AGC) target of 5 × 10^5^ and a maximum injection time of 200 ms. AIF spectra were acquired at a resolution setting of 17,500, with an AGC target of 5 × 10^5^ and a maximum injection time of 200 ms, using higher-energy collisional dissociation (HCD) at a single absolute collision energy of 35 eV. One microscan was used for each scan type, and the loop count and TopN were both set to 5. The intensity threshold for data-dependent triggering was 3.3 × 10^5^. Charge-state exclusion was disabled, so singly charged and unassigned-charge ions were not excluded from triggering by this setting. Peptide matching was set to “preferred” isotope exclusion was enabled, and dynamic exclusion was 10 s. The spray voltage was set to 2.5 kV and the capillary temperature to 250 °C.

Grouped injection was used in the reported experiments: the three technical injections of each sample were run consecutively, and samples were injected in condition blocks in a fixed order (vehicle control, apixaban, rivaroxaban, edoxaban, dabigatran), with a blank injection following each condition block. Comparable protein-abundance profiles were obtained with the two arrangements. Based on these observations, grouped injections were used for subsequent experiments. Because injection sequences were balanced but not formally randomized, injection order is described here as balanced rather than randomized. Complete sample and injection sequences are provided in Supplementary Table S4.

Blank injections were inspected for residual peptide signals. A retrospective comparison of the available peak-area measurements showed characteristic peptide signals of approximately 85,000 in sample 15 and 300–500 in blank 3. These measurements corresponded to blank-to-sample peak-area ratios of approximately 0.35%–0.6% for the examined signals. This assessment was limited to the available sample–blank comparison and did not establish an upper carryover limit across the complete dataset. A predefined numerical carryover acceptance threshold could not be verified from the available records.

Before each week-long LC–MS/MS sequence, the Exactive plus mass spectrometer was calibrated using Thermo Scientific Pierce ESI calibration solutions in both negative-ion (Ref. 88,324) and positive-ion (Ref. 88,323) modes, as part of routine mass-accuracy verification and instrument calibration across both polarities; the negative-ion solution was not used for, and has no role in, the acquisition of the positive-ion sample data reported here. System performance was additionally evaluated using the Pierce Peptide Retention Time Calibration Mixture (Ref. 88,321), used as an analytical system-suitability control. These reference materials were used as analytical system-suitability/performance controls, rather than as biological positive controls, to assess the performance of the chromatographic and mass-spectrometric system.

### 2.5 Data processing and protein identification

Raw LC–MS/MS data were processed using Thermo Scientific Proteome Discoverer (version 2.5.0.400). Precursor-based label-free quantification was performed using the Minora Feature Detector node, which detects MS1 isotopic features and maps them to peptide-spectrum matches (PSMs) across raw files. Spectra were first searched with Sequest HT using a broader precursor mass tolerance (20 ppm) and fragment mass tolerance (0.5 Da), with static carbamidomethylation of cysteine (+57.021 Da) as the only specified modification, within the Spectrum Files RC node, to generate confident PSMs for precursor mass recalibration. Following recalibration, peptide and protein identification was performed with Sequest HT against *Homo sapiens* UniProt reference databases (sp_canonical, TaxID 9606, version 2025-06-18), and a common contaminants database (0602_Universal Contaminants.fasta, version 2022-06-02). Contaminant matches were flagged using the Protein Marker node and were excluded from biological interpretation and downstream pathway analyses. This final search used a precursor mass tolerance of 10 ppm and a fragment mass tolerance of 0.6 Da, with trypsin (Full) specified as the digestion enzyme (maximum two missed cleavage sites; peptide length 6–144 amino acids); b- and y-ion series, including neutral-loss and flanking ions, were used for spectral matching. Static carbamidomethylation of cysteine (+57.021 Da) and dynamic modifications of methionine oxidation (+15.995 Da), N-terminal acetylation (+42.011 Da), N-terminal methionine loss (−131.040 Da), and combined N-terminal methionine loss with acetylation (−89.030 Da) were specified, with a maximum of three equal and four total dynamic modifications permitted per peptide.

Peptide-spectrum matches (PSMs) validation was carried out with the Percolator node using a concatenated target–decoy database and *q*-value–based validation. Percolator was run for up to 10 iterations, and the final PSM list used for protein inference was generated from the iteration with the highest number of PSMs at *q* < 0.01. Peptide-spectrum matches were filtered to a minimum confidence of medium and a minimum peptide length of six amino acids. PSMs, peptides, and protein groups were retained for quantification and downstream analyses when they met an identification-level FDR of <0.05, based on target–decoy validation. Display filters retained one master protein per group (“Master is equal to Master”) together with leaf-level (most specific, non-redundant) annotation and pathway protein groups. Retention-time alignment across raw files was performed with the Feature Mapper node; although the configured maximum alignment window was 10 min with a 10 ppm mass tolerance, the tolerance actually applied for feature mapping, calculated from the observed chromatographic reproducibility across runs, was 2.00 min and 5.2 ppm.

### 2.6 Statistical analysis

Quantification was based on precursor ion intensities using unique and razor peptides, and protein abundances were calculated as summed peptide abundances. Data were normalized to the total peptide amount per sample and scaled so that the average abundance per protein and peptide was set to 100. Quantification was performed using “Treatment” as the grouping variable. Pairwise protein abundance ratios were calculated for each DOAC versus the common vehicle control as (apixaban/control), (rivaroxaban/control), (edoxaban/control), and (dabigatran/control) using a pairwise ratio-based approach on log-transformed, normalized abundances. Differential abundance was assessed using the background-based *t*-test, which compares each protein’s abundance ratio to the distribution of all protein ratios in the dataset rather than estimating variance from that protein’s own replicates. No imputation was applied. Proteins with excessive missing values across replicates were excluded from the differential analysis by the default filtering in Proteome Discoverer. Differentially abundant proteins were defined as Benjamini–Hochberg FDR-adjusted *P* value, *q*-value <0.05 and |log_2_FC| > 1 (corresponding to an abundance ratio >2 or <0.5).

### 2.7 Bioinformatic analyses

Venn diagrams and heatmaps showing pairwise Jaccard similarity indices were generated using “Multiple List Comparator” *Molbiotools*, (https://molbiotools.com/listcompare.php). Differentially abundant proteins (DAPs) were subjected to functional enrichment analysis to identify significantly overrepresented biological processes and pathways. Gene Ontology (GO) enrichment analysis was performed for the Biological Process (BP) category, and Kyoto Encyclopedia of Genes and Genomes (KEGG) pathway enrichment analysis was conducted using the human reference dataset to characterize the biological functions associated with the identified DAPs. Enrichment analyses were performed using the SRplot online platform, which integrates the R packages clusterProfiler and pathview. KEGG enrichment pathways and their corresponding genes were visualized using Sankey–dot plots. The Gene Ratio was calculated as the count of input DAPs mapped to a specific pathway divided by the total number of annotated background genes belonging to that pathway (Gene Ratio = Count/M). GO BP cnet plots were generated to illustrate the relationships between enriched biological processes and associated DAPs (Tang et al., 2023). For functional enrichment analyses, differentially expressed proteins were mapped to their corresponding gene identifiers. This mapping was used only for Gene Ontology and KEGG pathway enrichment; all primary data and interpretations are at the protein level. Subnetwork analysis was performed using the Gene Ontology module of Enrichr-KG (https://maayanlab.cloud/enrichr-kg), connecting gene identifiers mapped from the drug-specific DAPs to the top-ranked enriched terms from the GO_Biological_Process_2021 library. Statistical significance was assessed using Fisher’s exact test (Evangelista et al., 2023).

## 3 Results

### 3.1 Effect of direct oral anticoagulants on HUVEC viability

To determine whether the selected DOAC concentrations affected endothelial cell metabolic activity, HUVECs were exposed to apixaban, rivaroxaban, edoxaban, or dabigatran for 6 h, and cell metabolic activity was assessed by MTT assay. Overall, treatment with the tested DOACs was not associated with a statistically significant difference in MTT metabolic activity compared to vehicle-treated control cells (one-way ANOVA, F(4,25) = 2.175, *P* = 0.101). Individual comparisons are presented in Table 1.

**TABLE 1:** MTT metabolic activity after exposure to direct oral anticoagulants by MTT assay. MTT metabolic activity was normalized to control cells (100%). Data are presented as mean ± SD, P-values were calculated by one-way ANOVA with Dunnett’s multiple comparisons test.

Treatment	Drug-specific DAPs	Higher- abundance	Lower-abundance
APIXABAN	189	76	113
RIVAROXABAN	186	106	80
EDOXABAN	182	83	99
DABIGATRAN	196	90	106
Sum of drug-specific DAPs	753	355	398

### 3.2 DOAC-induced differentially abundant proteins

LFQ proteomic analysis of HUVECs treated with apixaban, rivaroxaban, edoxaban, or dabigatran identified 1,643 protein entries and 953 protein groups, together with 999 included peptide groups and 4,877 included peptide-spectrum matches (PSMs). The estimated target-decoy FDRs - 0.8% for PSMs, 1.5% for peptide groups, 1.3% for protein entries, and 1.6% for protein groups - correspond to the combined medium- and high-confidence dataset, accepted at up to the 5% relaxed FDR threshold. These identification statistics define the protein dataset used for subsequent quantitative and pathway analyses.

Protein-level differential abundance was tested in Proteome Discoverer 2.5 using a background-based t-test (Section 2.6). Differentially abundant proteins (DAPs) were summarized in Table 2. The value of 753 represents the sum of drug-specific DAP calls across the four comparisons (189 for apixaban, 186 for rivaroxaban, 182 for edoxaban, and 196 for dabigatran) and includes proteins meeting the DAP criteria in more than one comparison; it does not represent 753 unique proteins. Details on protein accession numbers by UniProt and their correspondence to gene symbols are provided in Supplementary Table S1 (Supplementary).

**TABLE 2:** Sum of comparison-specific differential-abundance calls; not unique proteins with q < 0.05 and an abundance ratio >2 for higher abundance than vehicle or <0.5 for lower abundance than vehicle. The same protein may contribute to more than one drug-specific count.

Treatment	MTT metabolic activity (% of control, mean ± SD)	*P*-value, Dunnett-adjusted
Apixaban	100.4 ± 5.5	0.9998
Rivaroxaban	91.04 ± 3.91	0.0861
Edoxaban	91.41 ± 9.02	0.1045
Dabigatran	91.58 ± 7.69	0.1145

Volcano plots were generated using Proteome Discoverer 2.5 software using the FDR-adjusted *P* value (*q*) for the y-axis; the plotted threshold (*q* < 0.05, |log_2_FC| > 1) corresponds directly to the final DAP classification criterion reported in Table 2. Proteins with higher and lower abundance meeting this criterion for each drug comparison are shown in Figure 1.

**FIGURE 1:**
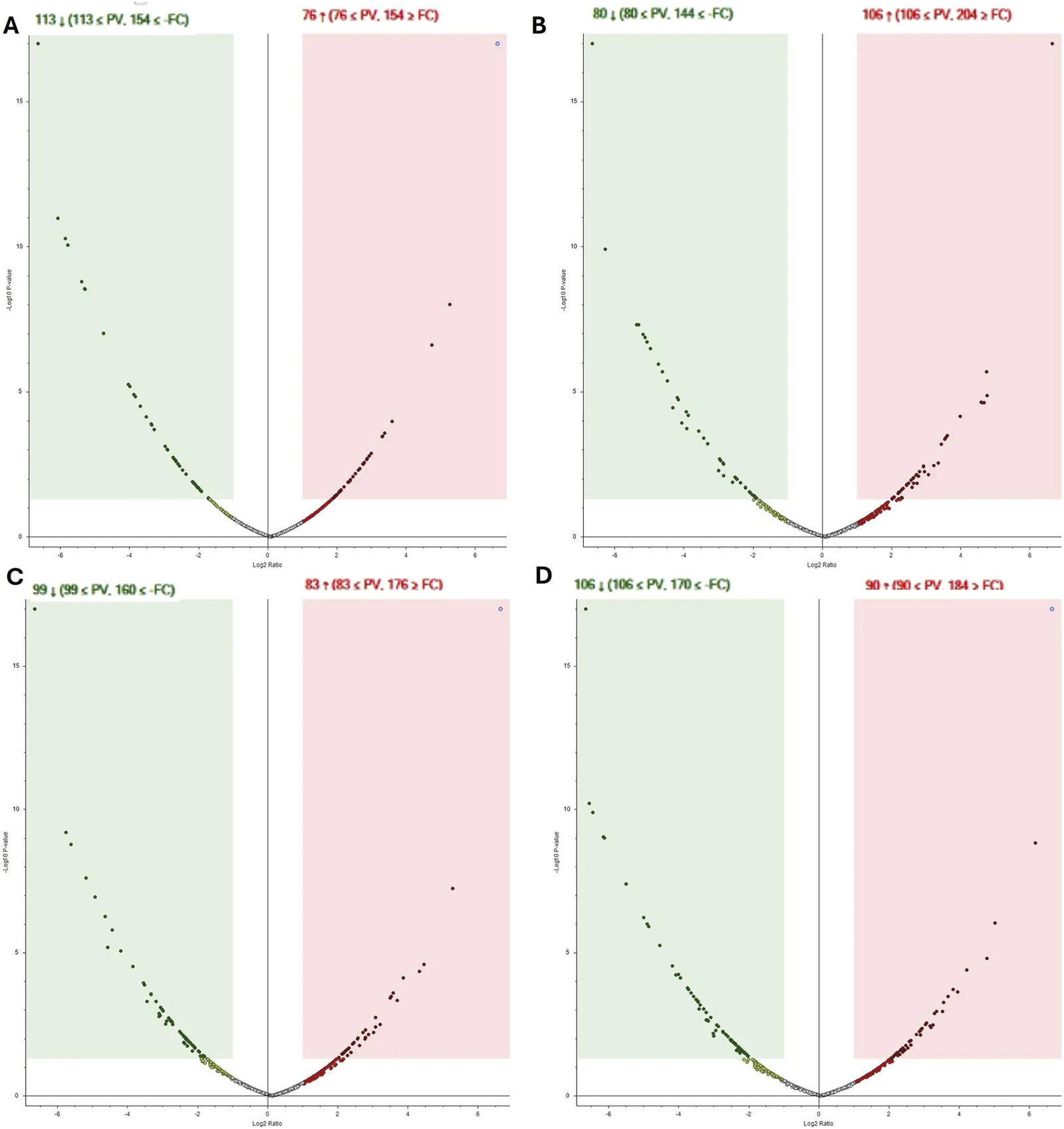
Volcano plots of protein abundance ratios versus vehicle control for **(A)** apixaban, **(B)** rivaroxaban, **(C)** edoxaban, and **(D)** dabigatran, generated in Proteome Discoverer 2.5. The y-axis displays the Benjamini–Hochberg FDR-adjusted P value; the plotted counts and shaded regions correspond directly to the final DAP classification threshold (q < 0.05, |log_2_FC| > 1) reported in Table 2.

### 3.3 Common DAPs in all four DOACs

Venn diagram analysis demonstrated extensive overlap of higher-abundance proteins across all four DOACs (Figure 2A). The analysis revealed thirty-two proteins commonly higher-abundance with apixaban, rivaroxaban, edoxaban, and dabigatran (B3EWG6, O14526-3, O15212, O43566, O75185, P07205, P0CB38, P46100, Q16555, Q16613-2, Q2TAZ0-4, Q6ZMM2, Q7Z5A7, Q7Z5Y7-3, Q8IUI8, Q8IWT0, Q8N859, Q8N878, Q8NDI1, Q8NE28-3, Q8NEX6, Q8TE54, Q8WVY7, Q96DT6, Q9BTD8, Q9H0Q0, Q9NWW7, Q9NWY4, Q9NX31, Q9Y237, Q9Y2Q3, Q9Y4R7-6). Pairwise comparisons showed quite similar overlaps with greater similarity between rivaroxaban and dabigatran relative to other drug combinations, as reflected by higher Jaccard indices in the corresponding similarity matrix (Figure 2B).

**FIGURE 2:**
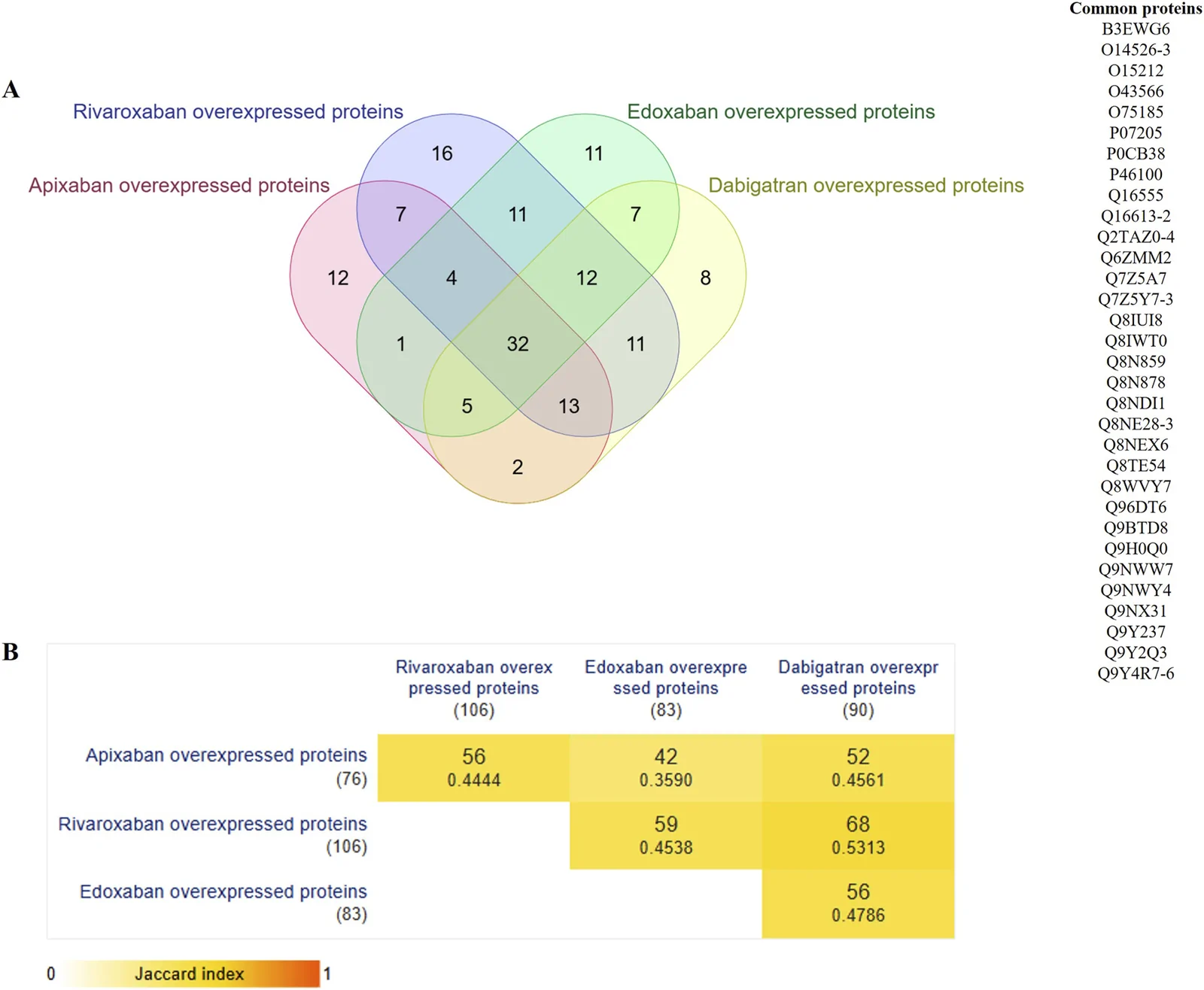
Overlap of higher-abundance proteins meeting the differential-abundance criteria in the DOAC-versus-vehicle comparisons in HUVECs. **(A)** Venn diagram illustrates common and unique higher-abundance proteins across DOAC treatments, while **(B)**. heatmap shows pairwise Jaccard similarity indices. Generated with Molbiotools.com.

A similar pattern was observed for lower-abundance proteins. Twenty-six proteins were commonly lower-abundance across all four DOACs (A9UHW6, O14490-4, O75952-6, O96028-2, P01034, P11230, P22749-2, P27037, P57729, P61371, Q02045, Q03013, Q3T906, Q5JUX0, Q5SRD1, Q6AWC2-2, Q6UWN5, Q86TL0-2, Q8NCK7, Q8WU43, Q96MU8, Q9BRS8, Q9BSQ5-2, Q9H9T3-2, Q9UIY3, Q9UJ70-2) (Figure 3A). Pairwise overlaps were quite similar, with the highest overlap observed between dabigatran and edoxaban (n = 59), whereas rivaroxaban and edoxaban shared comparatively less lower-abundance targets (n = 40), consistent with lower Jaccard similarity indices across treatments (Figure 3B).

**FIGURE 3:**
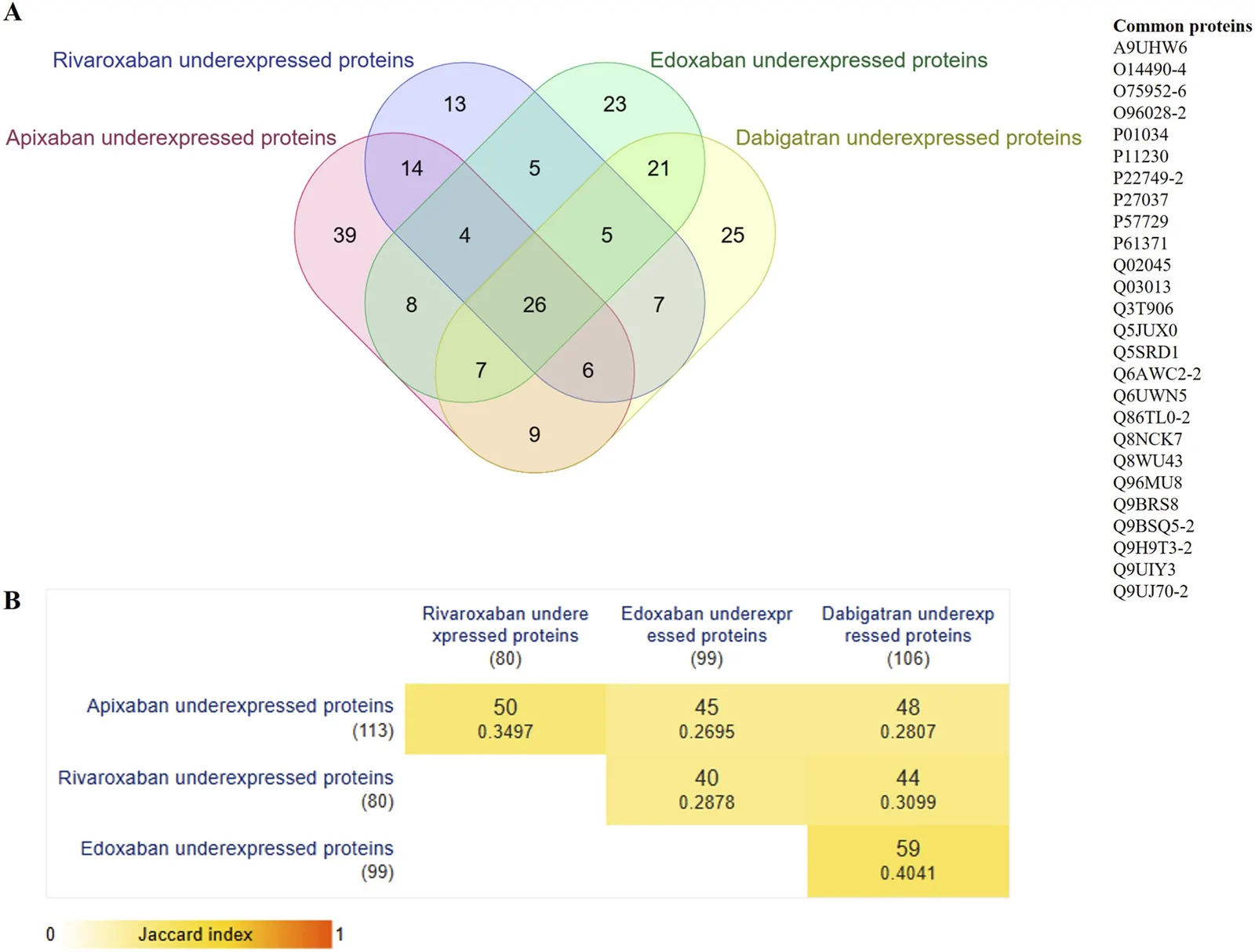
Overlap of lower-abundance proteins meeting the differential-abundance criteria in the DOAC-versus-vehicle comparisons in HUVECs. **(A)** Venn diagram illustrates common and unique lower-abundance proteins across DOAC treatments, while **(B)**. heatmap shows pairwise Jaccard similarity indices. Generated with Molbiotools.com.

Pathway and biological process enrichment analysis revealed the implicated pathways and biological processes of commonly higher or lower-abundance proteins. DAPs were mapped to their corresponding gene identifiers for enrichment pathway analyses. Among higher-abundance proteins mapped to gene identifiers for enrichment analysis, KEGG enrichment identified three significantly enriched pathways, including autophagy (hsa04136, hsa04140) and Hippo signaling (hsa04392) (Figure 4A). Autophagy was significantly enriched with the highest enrichment score (3.14) and fold enrichment (48.94). In addition, Hippo signaling pathway was also represented by the higher-abundance protein *FRMD1*, showing a high fold enrichment (27.0) with *P* = 0.03. Among lower-abundance proteins mapped to gene identifiers for enrichment analysis, KEGG analysis identified six statistically significant enriched pathways (Figure 4B). The most enriched pathway was Adherens junction (hsa04520), represented by *MYL5* and *CCM2*, with a fold enrichment of 16.84. Also, fluid shear stress and atherosclerosis (hsa05418) and signaling pathways regulating pluripotency of stem cells (hsa04550), both involving *ACVR2A* and *ISL1* gene identifiers, with fold enrichments of 11.03 and 10.88, respectively, were identified. Finally, enriched pathways included autophagy (hsa04136), represented by *ATG4D*, and metabolic pathways related to amino sugar and nucleotide sugar metabolism and amino sugar biosynthesis (hsa01250, hsa00520) represented by *NAGK*. Additional information is provided (Supplementary Table S2, Supplementary).

**FIGURE 4:**
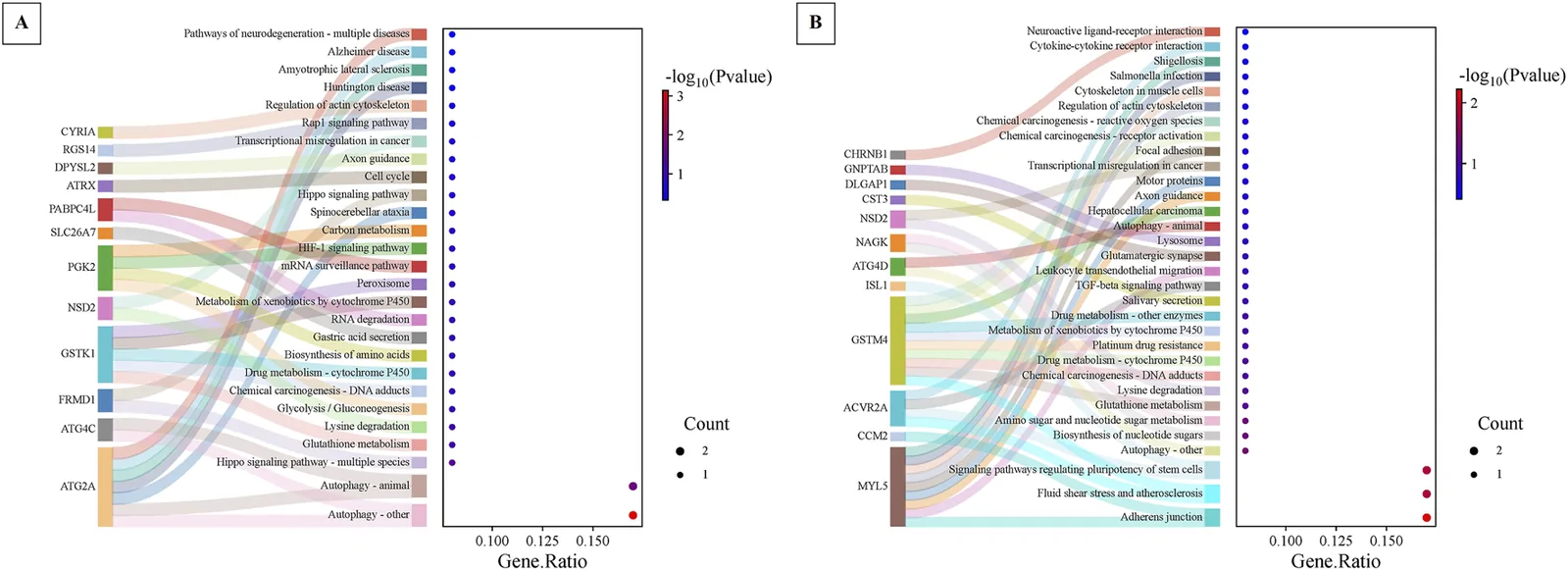
Sankey and dot plot from KEGG pathway enrichment analysis of common DAPs. DAPs were mapped to their corresponding gene identifiers for enrichment analysis using the SRplot tool. The Sankey diagram (left) illustrates the direct mapping and mapping between gene identifiers mapped from differentially abundant proteins and enriched KEGG pathway terms. The width of each connection line is proportional to the number of gene identifiers contributing to each pathway. The dot plot (right) summarizes the enrichment metrics for each pathway. The x-axis indicates the Gene Ratio (the number of input proteins mapped to the pathway divided by the total pathway size). The size of each dot corresponds to the number of mapped gene identifiers, while the color gradient represents the statistical significance based on P-value < 0.05. **(A)** corresponds to common higher-abundance proteins, and **(B)**. corresponds to common lower-abundance proteins.

Regarding the biological processes of the corresponding genes of commonly higher or lower- abundance proteins, higher-abundance proteins mapped to gene identifiers were enriched in chromatin remodeling, histone methylation, genome maintenance, RNA processing, and ion transport with key contributors including *ATRX*, *NSD2*, *ZBTB8OS*, *ATP2C2*, and *SLC26A7* (Figure 5A). For lower-abundance proteins mapped to gene identifiers, GO Biological Process terms included atrial septum and cardiac chamber development and morphogenesis, axon regeneration and response to axon injury, collagen metabolic process and carbohydrate phosphorylation, contributed by *ISL1*, *NSD2*, *LARP6*, *CST3*, *GNPTAB* and *NAGK* (Figure 5B). Most terms were supported by one or two mapped identifiers (Supplementary Table S2, Supplementary).

**FIGURE 5:**
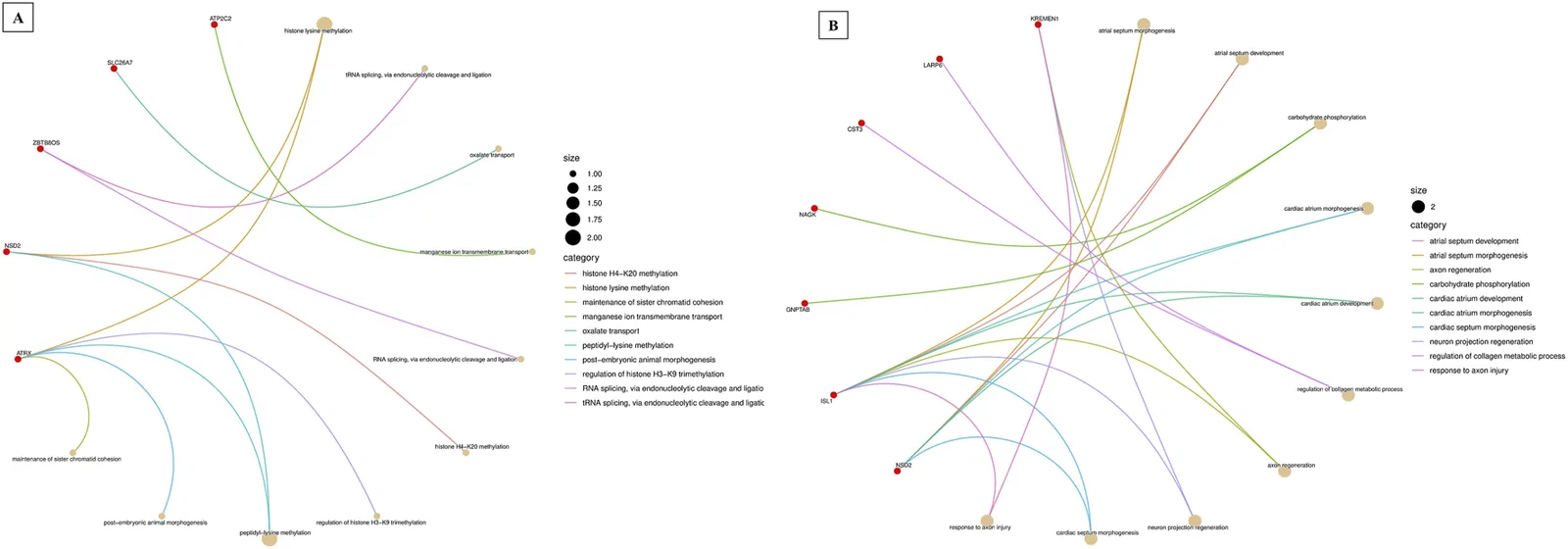
GO Biological Process (BP) cnet plot generated using SRplot (clusterProfiler-based analysis), showing associations between gene identifiers mapped from common differentially abundant proteins and enriched GO biological-process terms. DAPs were mapped to their corresponding gene identifiers for enrichment analysis **(A)**. corresponds to higher-abundance proteins, and **(B)**. corresponds to lower-abundance proteins. Red dots represent the mapped gene identifiers and beige dots the biological processes, the statistical significance based on P-value < 0.05.

### 3.4 Unique DAPs per drug

The majority of DAPs were found to be unique per drug with 51 counts for apixaban and 29, 34, 33 respectively for rivaroxaban, edoxaban and dabigatran according to Venn diagram analysis (Supplementary Table S3, Supplementary).

Drug specific DAPs were mapped to gene identifiers and analyzed with Enrichr-KG to generate drug-specific subnetworks (Figure 6). For apixaban, *PLEKHG5* was annotated to endothelial cell chemotaxis and protein signal transduction (GO:0035767, GO:0007265, GO:0007266). For rivaroxaban *SLC6A6* and *SCNN1D* were annotated to inorganic cation transmembrane transport (GO:0098662), sodium ion transport (GO:0006814), and sodium ion transmembrane transport (GO:0035725). *SLC6A6* was also annotated to positive regulation of necrotic cell death (GO:0010940). For edoxaban *YTHDC1* was annotated to regulation of alternative mRNA splicing via spliceosome (GO:0000381), regulation of RNA splicing (GO:0043484), as well as post-transcriptional regulation of gene expression (GO:0010608). Finally, for dabigatran *ZNF568* and *IGF2* were annotated to chordate and utero embryonic development (GO:0043009, GO:0001701), regulation of transcription by RNA polymerase II (GO:0006357) and positive regulation of catalytic activity (GO:0043085).

**FIGURE 6:**
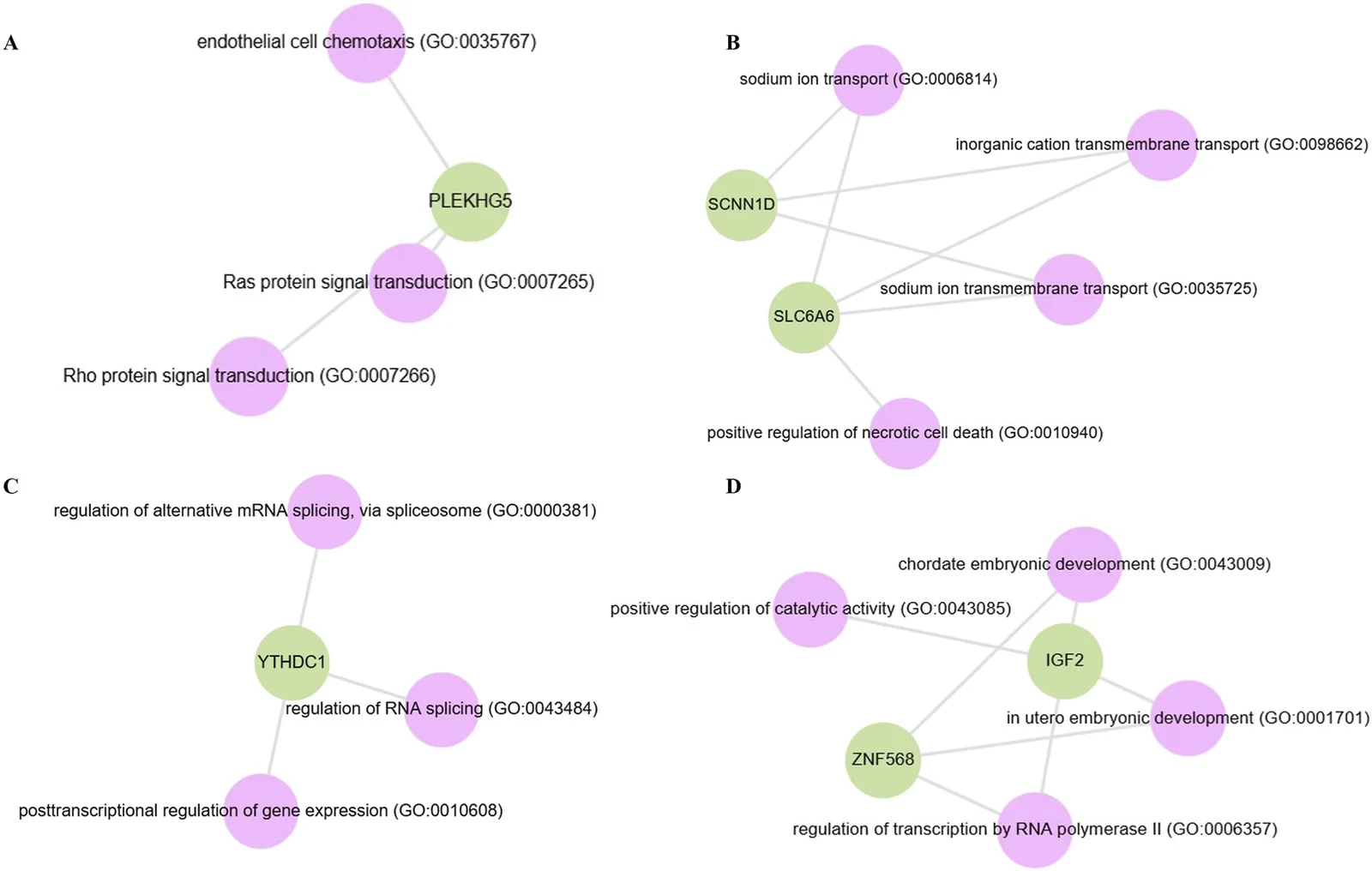
Enrichr-KG GO Biological Process subnetworks for gene identifiers mapped from the DAPs classified as specific to each treatment-versus-control comparison. Green nodes: gene-symbol nodes represent identifiers mapped from measured proteins for enrichment analysis; gene expression was not measured. Pink nodes: GO_Biological_Process_2021 terms enriched among these identifiers (nominal P < 0.05), connected by edges denoting gene-term membership; edges do not imply a demonstrated functional effect. **(A)** apixaban, **(B)** rivaroxaban, **(C)** edoxaban, **(D)** dabigatran.

### 3.5 FXa inhibitors DAPs

Restricting the analysis to the three factor Xa inhibitors (apixaban, rivaroxaban, and edoxaban), overlap of DAPs in Venn diagram analysis remained at quite similar level compared to the analysis of all four DOACs together. Thirty-five proteins were commonly higher-abundance (B3EWG6, O14526-3, O15212, O43566, O75185, P07205, P0CB38, P46100, Q16555, Q2TAZ0-4, Q32NC0-2, Q5SYB0-2, Q7Z5A7, Q7Z5Y7-3, Q8IUI8, Q8IWT0, Q8N859, Q8N878, Q8NDI1, Q8NE28-3, Q8NEX6, Q8TE54, Q8WVY7, Q96DT6, Q9BTD8, Q9H0Q0, Q9H6Z9, Q9NWW7, Q9NWY4, Q9NX31, Q9Y237, Q9Y2Q3, Q9Y4R7-6, Q16613-2, Q6ZMM2) and thirty lower-abundance proteins were common among the three factor Xa inhibitors (A9UHW6, O14490-4, O75952-6, O94827-6, O96028-2, P01034, P11230, P22749-2, P27037, P36575, P57729, P61371, Q02045, Q03013, Q3T906, Q5JUX0, Q5SRD1, Q6AWC2-2, Q6UWN5, Q86TL0-2, Q8N584-2, Q8NCK7, Q8WU43, Q96MU8, Q9BRS8, Q9BSQ5-2, Q9H172-3, Q9H9T3-2, Q9UIY3, Q9UJ70-2) (Figures 7, 8). Of these, three proteins were found to be higher-abundance (Q32NC0-2, Q5SYB0-2, Q9H6Z9) and four were lower-abundance (O94827-6, P36575, Q8N584-2, Q9H172-3) exclusively in response to FXa inhibitors, but not dabigatran. Accordingly, dabigatran versus FXa comparisons are potentially solvent confounded and should be interpreted with caution. Overall, overlap analyses and Jaccard similarity indices revealed that the three FXa inhibitors shared a more similar proteomic signature than their comparison with dabigatran.

**FIGURE 7:**
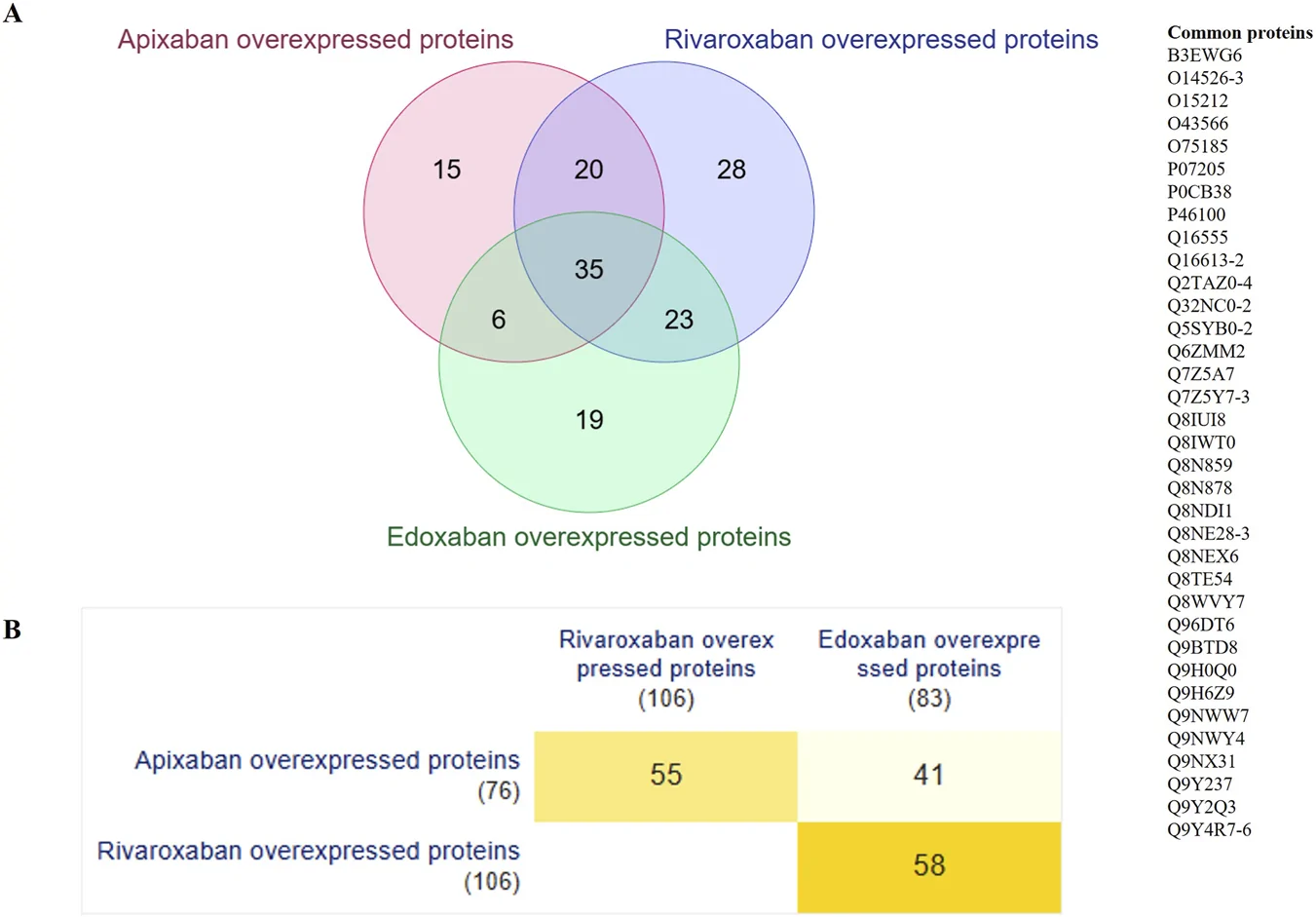
Overlap of FXa higher-abundance proteins in HUVECs. **(A)** Venn diagram illustrates common and unique proteins across FXa treatments, while **(B)**. heatmap shows pairwise Jaccard similarity indices. Generated with Molbiotools.com.

**FIGURE 8:**
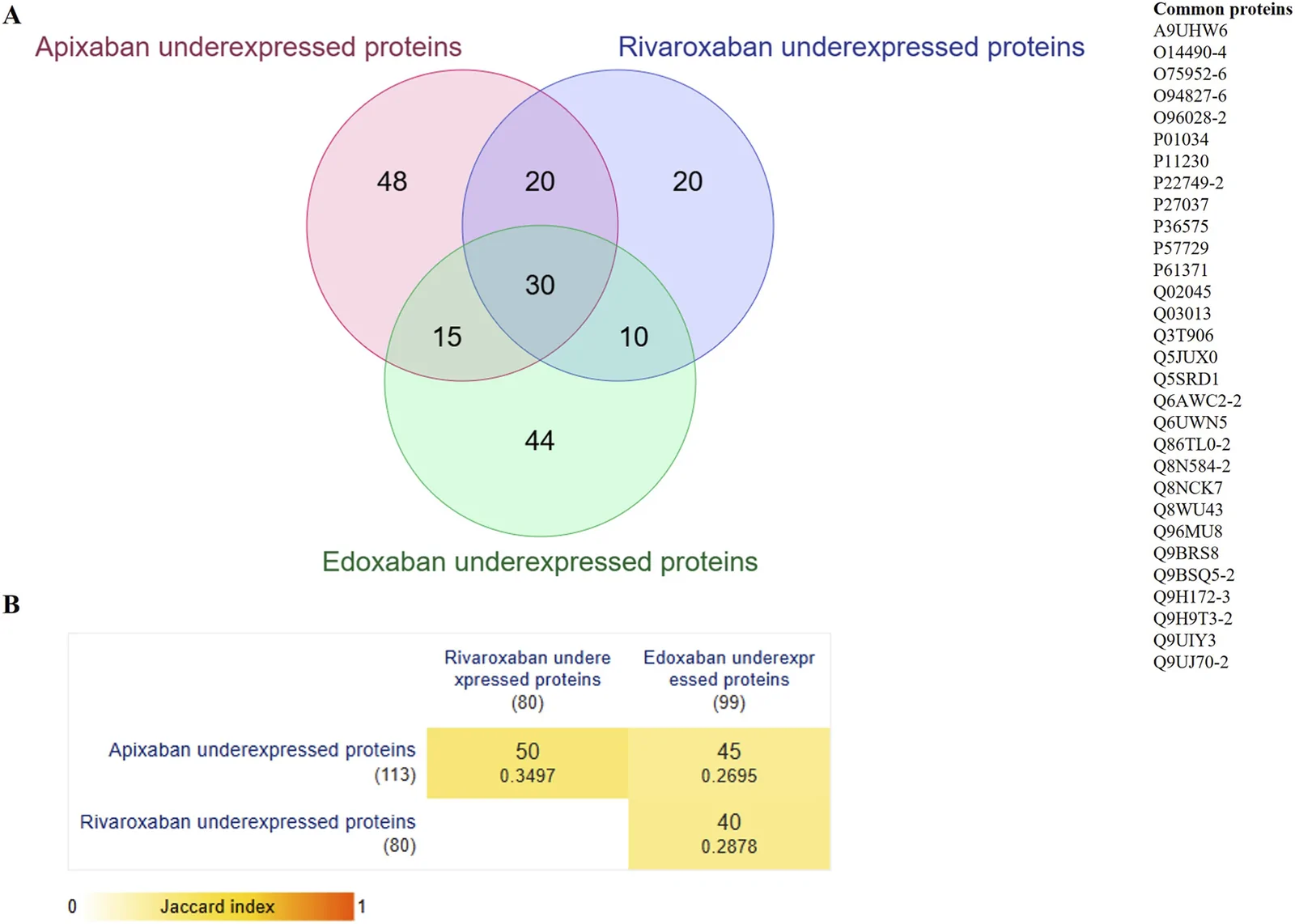
Overlap of FXa lower-abundance proteins in HUVECs. **(A)** Venn diagram illustrates common and unique proteins across FXa treatments, while **(B)**. heatmap shows pairwise Jaccard similarity indices. Generated with Molbiotools.com.

## 4 Discussion

This discovery-scale LFQ proteomic analysis of all four DOACs in unstimulated HUVECs identified DOAC-associated protein-abundance patterns and enrichment annotations linked to homeostasis (ion transport, autophagy) combined with drug-specific protein signatures, suggestive of heterogeneous proteomic responses on the vasculature. Using LFQ proteomics, we compared protein-abundance profiles following exposure to clinically relevant concentrations of direct oral anticoagulants on the protein expression profile of HUVECs, identifying 753 comparison-specific differential-abundance calls, including proteins represented in more than one treatment comparison (FDR *q* < 0.05, |log_2_FC|>1) that give some insight into their common as well as the distinct proteomic responses. While 58 DAPs overlapped across all DOACs (32 higher-abundance, 26 lower-abundance), many proteins met the differential-abundance criteria in only one treatment-versus-control comparison (e.g., 51 apixaban-unique), with Jaccard indices highlighting intra-class heterogeneity even among FXa inhibitors (apixaban-edoxaban: 0.27 lower-abundance overlap). These distinct proteomic profiles suggest potential differences, providing hypotheses that may be relevant to proposed non-anticoagulant effects within the DOAC class, although the functional relevance of these findings remains to be established.

To the authors’ knowledge, published proteomic studies involving DOAC exposure have predominantly examined rivaroxaban. A label-free quantification proteomics approach using patient plasma samples found that several proteins related to systemic inflammation and cellular stress were consistently more abundant in patients exposed to rivaroxaban (Feliu et al., 2026). Two additional studies that used comparative label-free quantitative proteomic profiling of enriched plasma extracellular vesicles from patients anticoagulated with either rivaroxaban or warfarin suggested that rivaroxaban may exert additional pleiotropic effects beyond its anticoagulant activity, including anti-inflammatory actions (Weiss et al., 2024; Weiss et al., 2021). We did not identify published studies performing proteomic profiling of apixaban, edoxaban, or dabigatran in any *in vitro* model.

Among proteins with commonly higher-abundance across all four DOACs, autophagy-related terms were enriched and were supported by the mapped identifiers *ATG2A* and *ATG4C*. In endothelial cells, autophagy plays a critical role in organelle quality control, redox balance, metabolic adaptation, and cell survival under stress conditions (Jiang, 2016). Within the cardiovascular system, autophagy and mitophagy interface tightly with redox signaling to preserve protein and organelle quality control and metabolic homeostasis across cardiomyocytes, endothelial cells, vascular smooth muscle cells, and immune cells (Yun et al., 2025). More broadly, autophagy is also a key regulator of cardiac resilience, influencing ischemia tolerance, remodeling, and cell survival in the heart (Jiang, 2016; Aljakna Khan and Sabatasso, 2025). Previous studies further support a link between DOACs and autophagy-related cardiovascular protection. Rivaroxaban has been shown to attenuate atherogenesis by inhibiting factor Xa–PAR2-mediated suppression of macrophage autophagy and by reducing inflammasome activation *in vivo* (Ito et al., 2021). The enrichment analysis returned a Hippo-related annotation represented by a single mapped identifier. In endothelial cells, including HUVECs, the Hippo/YAP/TAZ axis regulates mechanotransduction, junctional homeostasis, vasculogenesis, and barrier formation (Kim et al., 2026; Boopathy and Hong, 2019). YAP/TAZ activity is tightly linked to cell–cell interaction and VE-cadherin-dependent adhesion. Moreover, VE-cadherin signaling has been reported to promote cell migration while maintaining barrier integrity. In this context, the observed enrichment of Hippo-related terms may reflect annotation of these proteins to processes involved in endothelial adhesion and mechanosensitive signaling rather than a direct canonical effect of these drugs on the Hippo pathway (Neto et al., 2018). However, pathway level interpretations are hypothesis generating and require confirmation with orthogonal and functional approaches. In addition, proteins showing higher abundance were mapped to biological-process annotations related to epigenetic regulation, particularly histone lysine methylation (*H3K9*, *H3K36*, *H4K20*), chromatin remodeling (*ATRX*, *NSD2*, *HPF1*), ion transport (*ATP2C2*, *SLC26A7*) and autophagy (*ATG2A*) (Grigoriou et al., 2025; Epstein et al., 2015; Chen et al., 2021). Some of these proteins have previously been associated with cardiovascular adaptive mechanisms; however, such processes were not directly evaluated in the present study. These interpretations remain exploratory, as they are based on discovery proteomics and *in silico* enrichment without orthogonal or functional validation.

Pathway enrichment analysis of proteins commonly showing lower abundance across all four DOACs returned an adherens-junction annotation, with contributions from proteins mapped to *MYL5* and *CCM2*. As adherens junctions play a central role in maintaining vascular integrity and regulating communication between endothelial cells, the lower abundance of these proteins may be indicative of endothelial signaling responses to DOAC treatment (Komarova et al., 2017; Dabravolski et al., 2025). Although direct evidence linking DOACs to the adherens junction pathway is limited, available *in vitro* studies suggest that these agents may preserve endothelial barrier integrity by limiting thrombin/PAR-1–mediated junctional disruption and maintaining VE-cadherin/ZO-1-associated tightness in endothelial models (Puech et al., 2019; Gorzelak-Pabiś et al., 2022). Another enriched pathway among proteins showing lower abundance was “Fluid shear stress and atherosclerosis pathway”, which plays a central role in vascular biology and contributes to the progression of atherosclerosis (Dabravolski et al., 2025; Siasos et al., 2007). Furthermore, gene identifiers mapped from proteins showing lower abundance were enriched for GO terms related to cardiac morphogenesis, with contributions from proteins mapped to *ISL1* and *NSD2*, which have been described as regulators of cardiac progenitor specification, and developmental gene regulation (Maruyama et al., 2019; Liu et al., 2025). Consistent with clinical context, DOACs are prescribed mostly to prevent thromboembolic events in AF and in deep vein thrombosis (DVT), conditions strongly associated with endothelial dysfunction, inflammation, and vascular repair mechanisms (Atzemian et al., 2023; Black et al., 2022; Okawa et al., 2023; Mele et al., 2024). Accordingly, proteomic profiling of DOAC-treated HUVECs may provide exploratory insights into endothelial pathways that could warrant further investigation in the context of these conditions.

Collectively, the enrichment analyses identified annotations related to autophagy, endothelial junction organization, shear-stress responses, and vascular signaling among gene identifiers mapped from proteins showing shared abundance changes. These analyses are exploratory, are supported in some cases by few mapped identifiers, and do not demonstrate alteration of the corresponding pathways or cellular functions.

Descriptive differences were observed between the sets of proteins meeting the differential-abundance criteria for the FXa inhibitors and dabigatran. Three proteins commonly showed higher abundance across the three FXa inhibitors but not by dabigatran. These include proteins mapped to *C18orf21*, *FRMPD1* and *EGLN3*, which in existing databases are annotated to processes related to endothelial stress, hypoxia-related responses, and intracellular signaling. *EGLN3* has been reported to regulate cellular adaptation to hypoxia and *C18orf21* has been reported to protect pulmonary endothelium by reducing vascular permeability (Deng et al., 2025; Harrington et al., 2020). *FRMPD1* is an adaptor protein that could activate the Hippo pathway (Rong et al., 2019). Also, four proteins commonly showed lower abundance (proteins mapped to *GEF720*, *ARR3*, *TTC39C*, *ABCG4* gene identifiers) across the three FXa inhibitors. *GEF720* is related to endothelial cell adhesion in vascular homeostasis, and *ABCG4* to cholesterol homeostasis (Schumacher and Benndorf, 2017; van Buul et al., 2014). No functional roles have been reported so far for *ARR3* and *TTC39C*. Collectively, proteins meeting the differential-abundance criteria in the three FXa-inhibitor comparisons but not in the dabigatran comparison were annotated to pathways related to endothelial stress, hypoxia response, vascular permeability, and cholesterol homeostasis. Given the limited number of proteins and the vehicle-related confounding, these findings should be interpreted cautiously.

The exploratory subnetwork analysis associated mapped identifiers from comparison-specific protein sets with different ontology terms. *PLEKHG5* isoform 7, mapped from an apixaban-specific DAP, has been reported as a regulator of small GTPases, linked to endothelial cell chemotaxis, Ras and Rho signaling (Witte et al., 2020; Iwatake et al., 2017; Spindler et al., 2010). In endothelial cells, Ras/Rho pathways are known from previous studies to participate in barrier integrity, activation of inflammation and migration. These findings raise the possibility that the observed apixaban-associated protein changes may be related to endothelial chemotaxis and intracellular signaling, which are critical for vascular repair and angiogenesis (Witte et al., 2020; Iwatake et al., 2017; Spindler et al., 2010). However, this interpretation requires functional validation. *SLC6A6* and *SCNN1D*, mapped from rivaroxaban-specific DAPs, contributed to GO annotations related to sodium ion transport, inorganic cation transmembrane transport, and positive regulation of necrotic cell death, suggesting a potential effect on membrane homeostasis, ion exchange and stress responses (Hill et al., 2022; Rong et al., 2023). No measurements of membrane homeostasis, ion transport, cellular stress, or necrotic cell death were performed; therefore, these annotations should not be interpreted as functional effects. In the edoxaban analysis, the measured protein mapped to the *YTHDC1* identifier, which is annotated to RNA/mRNA binding and transcript regulation. This finding indicates that proteins associated with RNA processing and post-transcriptional regulatory functions were identified among the edoxaban-associated changes. However, whether these changes alter RNA processing or related cellular functions cannot be assessed from the present proteomic data (Kumari et al., 2022). Finally, in the dabigatran analysis, measured proteins mapped to the *IGF2* and *ZNF568* gene identifiers, which were connected to i. utero/chordate embryonic development, ii. regulation of transcription by RNA polymerase II, and iii. positive regulation of catalytic activity. These are annotation relationships only and do not demonstrate activation of growth, transcriptional regulation, or endothelial remodeling (Alders et al., 2025; Maeng et al., 2009; Han et al., 2024). These findings raise the possibility that dabigatran-associated protein changes provide hypotheses concerning these annotated processes; however, changes in gene expression or transcriptional activity were not measured (Gorzelak-Pabiś et al., 2022). Dabigatran specific differential expression and network findings may be influenced by the vehicle difference and should be interpreted with caution. Overall, the enrichment analyses associated the mapped identifiers from the four treatment comparisons with different sets of ontology terms. These unique protein signatures may generate hypotheses for DOACs’ distinct pleiotropic effects on the endothelium, even if they belong to the same anticoagulant class (Atzemian et al., 2023). However, many of these drug-specific narratives are based on relatively small unique-DAP sets (29–51 proteins) and enrichment terms supported by few contributing proteins. All pathway-level and subnetwork interpretations should therefore be considered hypothesis-generating and interpreted cautiously given the limited number of contributing proteins and the absence of orthogonal or functional validation. Confirmation using targeted proteomic or transcriptomic approaches and functional assays is required.

A strength is the inclusion of treatment conditions for all four DOACs within the same general experimental framework, combined with LFQ proteomic coverage and stringent statistical filtering. However, some limitations should also be acknowledged. First, this study used discovery-oriented label-free proteomics followed by *in silico* pathway and enrichment analyses. It therefore focuses on steady-state protein abundance and does not capture post-translational modifications or rapid signaling events. Furthermore, measured proteins were mapped to corresponding gene identifiers solely for enrichment and ontology analyses, and enrichment analyses and the primary findings concern protein abundance; neither transcriptional regulation nor pathway activity was measured. Moreover, experiments were conducted in isolated HUVECs so the observed drug-specific proteins may not fully represent the integrated vascular response in patients receiving DOAC therapy. Besides, selected treatment concentrations correspond to the therapeutic range rather than the exact peak (C_max) or trough (C_min) plasma concentrations observed in patients. In addition, in this proteomic study the analytical workflow may have provided incomplete coverage of low abundance and highly hydrophobic proteins. An additional limitation is that dabigatran was prepared in 0.01 M HCl, whereas the FXa inhibitors were dissolved in DMSO, and no HCl-matched vehicle control was included. Thus, dabigatran-versus-FXa and dabigatran-versus-control comparisons may be subject to potential solvent-related confounding. For the DMSO-dissolved FXa inhibitors, the vehicle control contained the highest DMSO concentration used among the treatment conditions. Furthermore, a pooled biological QC sample and endothelial positive-control treatment were not included. Nevertheless, instrument calibration, analytical reference materials, blank injections, and carryover assessment were performed, and comparable results were obtained with grouped and interleaved injection arrangements. Finally, the proteins and pathways identified in this discovery-oriented study require validation in independent experimental datasets, additional endothelial models, and orthogonal assays, such as western blotting or RT-PCR, to confirm their reproducibility and biological relevance.

In conclusion, under the experimental conditions used, exposure of HUVECs to the four DOAC treatment conditions was associated with common and comparison-specific protein-abundance patterns. Gene identifiers mapped from these DAPs were enriched for several pathway and ontology annotations, including autophagy, adherens junction, and cardiac developmental terms, but the corresponding cellular functions and pathway activities were not directly measured. The identified differential proteomic abundance provides exploratory, hypothesis-generating insight into the pleiotropic actions of DOACs. Finally, our findings provide a basis for future experimental and translational studies aimed at evaluating the biological significance of these observations.

## Data Availability

The data presented in the study are deposited in the PRIDE repository (Perez-Riverol et al., 2025), accession number PXD082155 (DOI: 10.6019/PXD082155).
