# Supplementary material for: Label-free comparative proteomic analysis reveals distinct profiles of direct oral anticoagulants in human endothelial cells
Source: Front Pharmacol. 2026 Oct 28;17:1913833. doi: 10.3389/fphar.2026.1913833 (PMC13752542; doi:10.3389/fphar.2026.1913833)
Supplement: Supplementary file 1 [file Table1.docx]

Supplementary Material

## 1.1 **Table S1:** List of differentially expressed proteins.

List of all the proteins that were differentially expressed in HUVECs following apixaban, rivaroxaban, edoxaban and dabigatran treatment with inclusion criteria of FDR≤5% and q-value threshold of < 0.05 combined with an abundance ratio >2 for upregulated proteins or < 0.5 for downregulated proteins (log₂ fold-change values of ≥ 1 and ≤ −1). Columns (left to right) show the UniProt accession number (green for downregulated/red for upregulated), protein description, gene symbol, abundance ratio, log2 abundance ratio determined by Proteome Discoverer 2.5 software proteomic analysis and calculated p value, where p<0.05 was statistically significant.

### A) Apixaban

| Accession | Description | Gene Symbol | Abundance Ratio | Abundance Ratio (log2) | Abundance Ratio P-Value |
| --- | --- | --- | --- | --- | --- |
| Q03013 | Glutathione S-transferase Mu 4 [OS=Homo sapiens] | GSTM4 | 0,01 | -6,64 | 7,79042E-17 |
| Q5T124-8 | Isoform 8 of UBX domain-containing protein 11 [OS=Homo sapiens] | UBXN11 | 0,01 | -6,64 | 7,79042E-17 |
| Q9UFH2 | Dynein heavy chain 17, axonemal [OS=Homo sapiens] | DNAH17 | 0,01 | -6,64 | 7,79042E-17 |
| Q5JPH6 | Probable glutamate--tRNA ligase, mitochondrial [OS=Homo sapiens] | EARS2 | 0,01 | -6,64 | 7,79042E-17 |
| Q9NR30 | Nucleolar RNA helicase 2 [OS=Homo sapiens] | DDX21 | 0,01 | -6,64 | 7,79042E-17 |
| Q9NYL5 | 24-hydroxycholesterol 7-alpha-hydroxylase [OS=Homo sapiens] | CYP39A1 | 0,01 | -6,64 | 7,79042E-17 |
| Q15648 | Mediator of RNA polymerase II transcription subunit 1 [OS=Homo sapiens] | MED1 | 0,01 | -6,64 | 7,79042E-17 |
| Q9Y3Q8 | TSC22 domain family protein 4 [OS=Homo sapiens] | TSC22D4 | 0,01 | -6,64 | 7,79042E-17 |
| P42768 | Wiskott-Aldrich syndrome protein [OS=Homo sapiens] | WAS | 0,01 | -6,64 | 7,79042E-17 |
| O15527 | N-glycosylase/DNA lyase [OS=Homo sapiens] | OGG1 | 0,01 | -6,64 | 7,79042E-17 |
| O75952-6 | Isoform 6 of Calcium-binding tyrosine phosphorylation-regulated protein [OS=Homo sapiens] | CABYR | 0,01 | -6,64 | 7,79042E-17 |
| A2RU67 | Protein FAM234B [OS=Homo sapiens] | FAM234B | 0,01 | -6,64 | 7,79042E-17 |
| Q96PE3 | Inositol polyphosphate-4-phosphatase type I A [OS=Homo sapiens] | INPP4A | 0,01 | -6,64 | 7,79042E-17 |
| P61962 | DDB1- and CUL4-associated factor 7 [OS=Homo sapiens] | DCAF7 | 0,01 | -6,64 | 7,79042E-17 |
| Q15181 | Inorganic pyrophosphatase [OS=Homo sapiens] | PPA1 | 0,01 | -6,64 | 7,79042E-17 |
| Q96N06-2 | Isoform 2 of Spermatogenesis-associated protein 33 [OS=Homo sapiens] | SPATA33 | 0,01 | -6,64 | 7,79042E-17 |
| P48507 | Glutamate--cysteine ligase regulatory subunit [OS=Homo sapiens] | GCLM | 0,01 | -6,64 | 7,79042E-17 |
| P25963 | NF-kappa-B inhibitor alpha [OS=Homo sapiens] | NFKBIA | 0,01 | -6,64 | 7,79042E-17 |
| Q5VWQ8 | Disabled homolog 2-interacting protein [OS=Homo sapiens] | DAB2IP | 0,01 | -6,64 | 7,79042E-17 |
| Q5VZ52 | MORN repeat-containing protein 5 [OS=Homo sapiens] | MORN5 | 0,01 | -6,64 | 7,79042E-17 |
| Q86VX9-5 | Isoform 5 of Vacuolar fusion protein MON1 homolog A [OS=Homo sapiens] | MON1A | 0,01 | -6,64 | 7,79042E-17 |
| Q5HYR2 | Doublesex- and mab-3-related transcription factor C1 [OS=Homo sapiens] | DMRTC1; DMRTC1B | 0,01 | -6,64 | 7,79042E-17 |
| Q8N584-2 | Isoform 2 of Tetratricopeptide repeat protein 39C [OS=Homo sapiens] | TTC39C | 0,01 | -6,64 | 7,79042E-17 |
| Q2VPA4-2 | Isoform 2 of Complement component receptor 1-like protein [OS=Homo sapiens] | CR1L | 0,01 | -6,64 | 7,79042E-17 |
| Q96SR6-2 | Isoform 2 of Zinc finger protein 382 [OS=Homo sapiens] | ZNF382 | 0,01 | -6,64 | 7,79042E-17 |
| Q5JUX0 | Spindlin-3 [OS=Homo sapiens] | SPIN3 | 0,01 | -6,64 | 7,79042E-17 |
| P61018-2 | Isoform 2 of Ras-related protein Rab-4B [OS=Homo sapiens] | RAB4B | 0,01 | -6,64 | 7,79042E-17 |
| Q8N752 | Casein kinase I isoform alpha-like [OS=Homo sapiens] | CSNK1A1L | 0,01 | -6,64 | 7,79042E-17 |
| Q9NP81 | Serine--tRNA ligase, mitochondrial [OS=Homo sapiens] | SARS2 | 0,01 | -6,64 | 7,79042E-17 |
| O96028-2 | Isoform 2 of Histone-lysine N-methyltransferase NSD2 [OS=Homo sapiens] | NSD2 | 0,01 | -6,64 | 7,79042E-17 |
| A9UHW6 | MIF4G domain-containing protein [OS=Homo sapiens] | MIF4GD | 0,01 | -6,64 | 7,79042E-17 |
| Q16890-5 | Isoform 5 of Tumor protein D53 [OS=Homo sapiens] | TPD52L1 | 0,01 | -6,64 | 7,79042E-17 |
| Q9NP73-4 | Isoform 4 of Putative bifunctional UDP-N-acetylglucosamine transferase and deubiquitinase ALG13 [OS=Homo sapiens] | ALG13 | 0,01 | -6,64 | 7,79042E-17 |
| P12643 | Bone morphogenetic protein 2 [OS=Homo sapiens] | BMP2 | 0,01 | -6,64 | 7,79042E-17 |
| Q86WX3 | Active regulator of SIRT1 [OS=Homo sapiens] | RPS19BP1 | 0,01 | -6,64 | 7,79042E-17 |
| Q15257 | Serine/threonine-protein phosphatase 2A activator [OS=Homo sapiens] | PTPA | 0,01 | -6,64 | 7,79042E-17 |
| Q8N729 | Neuropeptide W [OS=Homo sapiens] | NPW | 0,01 | -6,64 | 7,79042E-17 |
| Q96RU2 | Ubiquitin carboxyl-terminal hydrolase 28 [OS=Homo sapiens] | USP28 | 0,01 | -6,64 | 7,79042E-17 |
| P22749-2 | Isoform Short of Granulysin [OS=Homo sapiens] | GNLY | 0,01 | -6,64 | 7,79042E-17 |
| O14782 | Kinesin-like protein KIF3C [OS=Homo sapiens] | KIF3C | 0,01 | -6,64 | 7,79042E-17 |
| Q3KNS6-3 | Isoform 3 of Zinc finger protein 829 [OS=Homo sapiens] | ZNF829 | 0,01 | -6,64 | 7,79042E-17 |
| P23588 | Eukaryotic translation initiation factor 4B [OS=Homo sapiens] | EIF4B | 0,01 | -6,64 | 7,79042E-17 |
| Q8IZ41 | Ras and EF-hand domain-containing protein [OS=Homo sapiens] | RASEF | 0,01 | -6,64 | 7,79042E-17 |
| Q9UBL6 | Copine-7 [OS=Homo sapiens] | CPNE7 | 0,01 | -6,64 | 7,79042E-17 |
| Q6SA08 | Testis-specific serine/threonine-protein kinase 4 [OS=Homo sapiens] | TSSK4 | 0,01 | -6,64 | 7,79042E-17 |
| P21754 | Zona pellucida sperm-binding protein 3 [OS=Homo sapiens] | ZP3 | 0,01 | -6,64 | 7,79042E-17 |
| Q9HCC6 | Transcription factor HES-4 [OS=Homo sapiens] | HES4 | 0,01 | -6,64 | 7,79042E-17 |
| Q6N043 | Zinc finger protein 280D [OS=Homo sapiens] | ZNF280D | 0,01 | -6,64 | 7,79042E-17 |
| Q9UIY3 | RWD domain-containing protein 2A [OS=Homo sapiens] | RWDD2A | 0,01 | -6,64 | 7,79042E-17 |
| Q9NXT0-2 | Isoform 2 of Zinc finger protein 586 [OS=Homo sapiens] | ZNF586 | 0,01 | -6,64 | 7,79042E-17 |
| Q9Y2D2 | UDP-N-acetylglucosamine transporter [OS=Homo sapiens] | SLC35A3 | 0,01 | -6,64 | 7,79042E-17 |
| Q5EBL8-2 | Isoform 2 of PDZ domain-containing protein 11 [OS=Homo sapiens] | PDZD11 | 0,01 | -6,64 | 7,79042E-17 |
| P01034 | Cystatin-C [OS=Homo sapiens] | CST3 | 0,01 | -6,64 | 7,79042E-17 |
| P61371 | Insulin gene enhancer protein ISL-1 [OS=Homo sapiens] | ISL1 | 0,01 | -6,64 | 7,79042E-17 |
| Q9UPV7 | PHD finger protein 24 [OS=Homo sapiens] | PHF24 | 0,01 | -6,64 | 7,79042E-17 |
| Q9UL46 | Proteasome activator complex subunit 2 [OS=Homo sapiens] | PSME2 | 0,01 | -6,64 | 7,79042E-17 |
| Q9H172-3 | Isoform 3 of ATP-binding cassette sub-family G member 4 [OS=Homo sapiens] | ABCG4 | 0,01 | -6,64 | 7,79042E-17 |
| Q9UBN7 | Histone deacetylase 6 [OS=Homo sapiens] | HDAC6 | 0,01 | -6,64 | 7,79042E-17 |
| Q8IYP9 | Palmitoyltransferase ZDHHC23 [OS=Homo sapiens] | ZDHHC23 | 0,01 | -6,64 | 7,79042E-17 |
| A0A2R8Y7Y5 | Uncharacterized protein OS=Homo sapiens OX=9606 GN=LOC729966 PE=4 SV=1 | CIST1 | 0,01 | -6,64 | 7,79042E-17 |
| O15085 | Rho guanine nucleotide exchange factor 11 [OS=Homo sapiens] | ARHGEF11 | 0,01 | -6,64 | 7,79042E-17 |
| Q92963-2 | Isoform 2 of GTP-binding protein Rit1 [OS=Homo sapiens] | RIT1 | 0,01 | -6,64 | 7,79042E-17 |
| Q9H9P5-3 | Isoform 3 of Putative E3 ubiquitin-protein ligase UNKL [OS=Homo sapiens] | UNKL | 0,01 | -6,64 | 7,79042E-17 |
| P0C870 | Bifunctional peptidase and (3S)-lysyl hydroxylase JMJD7 [OS=Homo sapiens] | JMJD7 | 0,01 | -6,64 | 7,79042E-17 |
| Q5U5R9 | Probable E3 ubiquitin-protein ligase HECTD2 [OS=Homo sapiens] | HECTD2 | 0,01 | -6,64 | 7,79042E-17 |
| O94827-6 | Isoform 6 of Pleckstrin homology domain-containing family G member 5 [OS=Homo sapiens] | PLEKHG5 | 0,01 | -6,64 | 7,79042E-17 |
| Q5SRD1 | Mitochondrial import inner membrane translocase subunit Tim23B OS=Homo sapiens OX=9606 GN=TIMM23B PE=1 SV=3 | TIMM23B | 0,01 | -6,64 | 7,79042E-17 |
| Q6AWC2-2 | Isoform 2 of Protein WWC2 [OS=Homo sapiens] | WWC2 | 0,01 | -6,64 | 7,79042E-17 |
| Q9H4B8 | Dipeptidase 3 [OS=Homo sapiens] | DPEP3 | 0,01 | -6,64 | 7,79042E-17 |
| Q9UQ13 | Leucine-rich repeat protein SHOC-2 [OS=Homo sapiens] | SHOC2 | 0,01 | -6,64 | 7,79042E-17 |
| A6NJZ3 | Olfactory receptor 6C65 [OS=Homo sapiens] | OR6C65 | 0,01 | -6,64 | 7,79042E-17 |
| Q9BSQ5-2 | Isoform 2 of Cerebral cavernous malformations 2 protein [OS=Homo sapiens] | CCM2 | 0,015 | -6,07 | 1,06979E-11 |
| Q8NCK7 | Monocarboxylate transporter 11 [OS=Homo sapiens] | SLC16A11 | 0,017 | -5,85 | 5,26115E-11 |
| Q15256-5 | Isoform 4 of Receptor-type tyrosine-protein phosphatase R [OS=Homo sapiens] | PTPRR | 0,018 | -5,78 | 8,79745E-11 |
| O00142 | Thymidine kinase 2, mitochondrial [OS=Homo sapiens] | TK2 | 0,024 | -5,38 | 1,58571E-09 |
| Q9BRD0 | BUD13 homolog [OS=Homo sapiens] | BUD13 | 0,026 | -5,29 | 2,96153E-09 |
| P27037 | Activin receptor type-2A [OS=Homo sapiens] | ACVR2A | 0,026 | -5,28 | 2,78962E-09 |
| O95382-2 | Isoform 2 of Mitogen-activated protein kinase kinase kinase 6 [OS=Homo sapiens] | MAP3K6 | 0,037 | -4,74 | 9,61396E-08 |
| Q86TL0-2 | Isoform 2 of Cysteine protease ATG4D [OS=Homo sapiens] | ATG4D | 0,061 | -4,02 | 5,58019E-06 |
| Q02045 | Myosin light chain 5 [OS=Homo sapiens] | MYL5 | 0,063 | -3,99 | 6,62295E-06 |
| Q8WU43 | Uncharacterized protein C2orf15 [OS=Homo sapiens] | C2orf15 | 0,069 | -3,86 | 1,27094E-05 |
| O95563 | Mitochondrial pyruvate carrier 2 [OS=Homo sapiens] | MPC2 | 0,07 | -3,83 | 1,48149E-05 |
| Q3T906 | N-acetylglucosamine-1-phosphotransferase subunits alpha/beta [OS=Homo sapiens] | GNPTAB | 0,078 | -3,68 | 3,18338E-05 |
| Q9BRS8 | La-related protein 6 [OS=Homo sapiens] | LARP6 | 0,088 | -3,5 | 7,26701E-05 |
| Q8IV38 | Ankyrin repeat and MYND domain-containing protein 2 [OS=Homo sapiens] | ANKMY2 | 0,096 | -3,37 | 0,000131486 |
| Q16769 | Glutaminyl-peptide cyclotransferase [OS=Homo sapiens] | QPCT | 0,098 | -3,36 | 0,000141516 |
| Q9H9T3-2 | Isoform 2 of Elongator complex protein 3 [OS=Homo sapiens] | ELP3 | 0,103 | -3,28 | 0,000199637 |
| Q8WXJ9 | Ankyrin repeat and SOCS box protein 17 [OS=Homo sapiens] | ASB17 | 0,129 | -2,96 | 0,000767067 |
| Q6UWN5 | Ly6/PLAUR domain-containing protein 5 [OS=Homo sapiens] | LYPD5 | 0,133 | -2,91 | 0,000945876 |
| Q14005 | Pro-interleukin-16 [OS=Homo sapiens] | IL16 | 0,134 | -2,89 | 0,000987397 |
| Q9BV57-2 | Isoform 2 of 1,2-dihydroxy-3-keto-5-methylthiopentene dioxygenase [OS=Homo sapiens] | ADI1 | 0,15 | -2,74 | 0,001812283 |
| Q9UJ70-2 | Isoform 2 of N-acetyl-D-glucosamine kinase [OS=Homo sapiens] | NAGK | 0,153 | -2,7 | 0,002041905 |
| P11230 | Acetylcholine receptor subunit beta [OS=Homo sapiens] | CHRNB1 | 0,158 | -2,66 | 0,002376546 |
| Q7L1S5 | Carbohydrate sulfotransferase 9 [OS=Homo sapiens] | CHST9 | 0,159 | -2,65 | 0,002509366 |
| P57729 | Ras-related protein Rab-38 [OS=Homo sapiens] | RAB38 | 0,166 | -2,59 | 0,003097208 |
| Q8WUS8 | Short-chain dehydrogenase/reductase family 42E member 1 [OS=Homo sapiens] | SDR42E1 | 0,172 | -2,54 | 0,003686071 |
| Q9NZ63 | Telomere length and silencing protein 1 homolog [OS=Homo sapiens] | C9orf78 | 0,182 | -2,46 | 0,004971089 |
| Q96MU8 | Kremen protein 1 [OS=Homo sapiens] | KREMEN1 | 0,182 | -2,46 | 0,004924984 |
| P36575 | Arrestin-C [OS=Homo sapiens] | ARR3 | 0,195 | -2,36 | 0,006886331 |
| O43896 | Kinesin-like protein KIF1C [OS=Homo sapiens] | KIF1C | 0,222 | -2,17 | 0,012651443 |
| Q13442 | 28 kDa heat- and acid-stable phosphoprotein [OS=Homo sapiens] | PDAP1 | 0,224 | -2,16 | 0,013001301 |
| Q494R0 | Putative uncharacterized protein FBXL19-AS1 [OS=Homo sapiens] | FBXL19-AS1 | 0,227 | -2,14 | 0,013913619 |
| Q9UKV3-2 | Isoform 2 of Apoptotic chromatin condensation inducer in the nucleus [OS=Homo sapiens] | ACIN1 | 0,228 | -2,13 | 0,014141137 |
| Q5HYI8 | Rab-like protein 3 [OS=Homo sapiens] | RABL3 | 0,234 | -2,09 | 0,015971922 |
| Q6ZQQ6 | WD repeat-containing protein 87 [OS=Homo sapiens] | WDR87 | 0,242 | -2,04 | 0,018517901 |
| O14490-4 | Isoform 4 of Disks large-associated protein 1 [OS=Homo sapiens] | DLGAP1 | 0,246 | -2,02 | 0,01968648 |
| Q6P996-3 | Isoform 3 of Pyridoxal-dependent decarboxylase domain-containing protein 1 [OS=Homo sapiens] | PDXDC1 | 0,249 | -2 | 0,020828663 |
| A8MWX3 | Putative WAS protein family homolog 4 [OS=Homo sapiens] | WASH4P | 0,257 | -1,96 | 0,023697192 |
| Q96JJ6 | Junctophilin-4 [OS=Homo sapiens] | JPH4 | 0,266 | -1,91 | 0,027227819 |
| Q9BQ16-6 | Isoform 6 of Testican-3 [OS=Homo sapiens] | SPOCK3 | 0,303 | -1,72 | 0,045813118 |
| Q9UK13 | Zinc finger protein 221 [OS=Homo sapiens] | ZNF221 | 0,305 | -1,71 | 0,046954574 |
| P32019-3 | Isoform 3 of Type II inositol 1,4,5-trisphosphate 5-phosphatase [OS=Homo sapiens] | INPP5B | 0,307 | -1,7 | 0,047718515 |
| Q96B86-4 | Isoform 3 of Repulsive guidance molecule A [OS=Homo sapiens] | RGMA | 0,308 | -1,7 | 0,04875003 |
| Q6ZMM2 | ADAMTS-like protein 5 [OS=Homo sapiens] | ADAMTSL5 | 3,682 | 1,88 | 0,0475941 |
| Q9UJA3 | DNA helicase MCM8 [OS=Homo sapiens] | MCM8 | 3,743 | 1,9 | 0,044750078 |
| P43251-3 | Isoform 3 of Biotinidase [OS=Homo sapiens] | BTD | 3,811 | 1,93 | 0,04178036 |
| Q14CB8-4 | Isoform 4 of Rho GTPase-activating protein 19 [OS=Homo sapiens] | ARHGAP19 | 3,832 | 1,94 | 0,040905641 |
| Q15072 | Zinc finger protein OZF [OS=Homo sapiens] | ZNF146 | 3,839 | 1,94 | 0,040597637 |
| Q16613-2 | Isoform 2 of Serotonin N-acetyltransferase [OS=Homo sapiens] | AANAT | 3,898 | 1,96 | 0,038288365 |
| Q9NYP8 | Uncharacterized protein C21orf62 [OS=Homo sapiens] | C21orf62 | 3,905 | 1,97 | 0,038008314 |
| Q13439 | Golgin subfamily A member 4 [OS=Homo sapiens] | GOLGA4 | 3,92 | 1,97 | 0,037447144 |
| Q06889-2 | Isoform 2 of Early growth response protein 3 [OS=Homo sapiens] | EGR3 | 3,929 | 1,97 | 0,037129985 |
| Q4G0J3 | La-related protein 7 [OS=Homo sapiens] | LARP7 | 3,981 | 1,99 | 0,035265905 |
| Q5JUR7 | Testis-expressed protein 30 [OS=Homo sapiens] | TEX30 | 4,023 | 2,01 | 0,033832908 |
| Q7Z5A7 | Chemokine-like protein TAFA-5 [OS=Homo sapiens] | TAFA5 | 4,176 | 2,06 | 0,029131441 |
| Q32NC0-2 | Isoform 2 of UPF0711 protein C18orf21 [OS=Homo sapiens] | C18orf21 | 4,204 | 2,07 | 0,02835747 |
| Q6UWP2 | Dehydrogenase/reductase SDR family member 11 [OS=Homo sapiens] | DHRS11 | 4,262 | 2,09 | 0,026822106 |
| Q9H6Z9 | Prolyl hydroxylase EGLN3 [OS=Homo sapiens] | EGLN3 | 4,287 | 2,1 | 0,026170903 |
| Q9NPC1 | Leukotriene B4 receptor 2 [OS=Homo sapiens] | LTB4R2 | 4,343 | 2,12 | 0,024816535 |
| O60809 | PRAME family member 10 [OS=Homo sapiens] | PRAMEF10 | 4,36 | 2,12 | 0,024427999 |
| Q5U651 | Ras-interacting protein 1 [OS=Homo sapiens] | RASIP1 | 4,367 | 2,13 | 0,024269674 |
| Q8NDI1 | EH domain-binding protein 1 [OS=Homo sapiens] | EHBP1 | 4,371 | 2,13 | 0,024163656 |
| Q01543-2 | Isoform 2 of Friend leukemia integration 1 transcription factor [OS=Homo sapiens] | FLI1 | 4,375 | 2,13 | 0,024073979 |
| Q969R2 | Oxysterol-binding protein 2 [OS=Homo sapiens] | OSBP2 | 4,628 | 2,21 | 0,019004068 |
| Q92771 | Putative ATP-dependent RNA helicase DDX12 [OS=Homo sapiens] | DDX12P | 5,017 | 2,33 | 0,013362832 |
| Q9P107 | GEM-interacting protein [OS=Homo sapiens] | GMIP | 5,099 | 2,35 | 0,012416649 |
| A6NJY4 | Putative transmembrane protein LOC100289255 [OS=Homo sapiens] | TMEM238L | 5,189 | 2,38 | 0,011481507 |
| O75185 | Calcium-transporting ATPase type 2C member 2 [OS=Homo sapiens] | ATP2C2 | 5,265 | 2,4 | 0,010737028 |
| P0CB38 | Polyadenylate-binding protein 4-like [OS=Homo sapiens] | PABPC4L | 5,287 | 2,4 | 0,010541828 |
| A0A0G2JND7 | Uncharacterized protein OS=Homo sapiens OX=9606 PE=4 SV=1 |  | 5,523 | 2,47 | 0,008605038 |
| P49765 | Vascular endothelial growth factor B [OS=Homo sapiens] | VEGFB | 5,524 | 2,47 | 0,008599632 |
| O94827-7 | Isoform 7 of Pleckstrin homology domain-containing family G member 5 [OS=Homo sapiens] | PLEKHG5 | 5,827 | 2,54 | 0,006675559 |
| P46100 | Transcriptional regulator ATRX [OS=Homo sapiens] | ATRX | 6,163 | 2,62 | 0,005080297 |
| Q9HB75-6 | Isoform 6 of p53-induced death domain-containing protein 1 [OS=Homo sapiens] | PIDD1 | 6,35 | 2,67 | 0,004376715 |
| P27695 | DNA-(apurinic or apyrimidinic site) endonuclease [OS=Homo sapiens] | APEX1 | 6,784 | 2,76 | 0,003127741 |
| Q96FC9-3 | Isoform 3 of ATP-dependent DNA helicase DDX11 [OS=Homo sapiens] | DDX11 | 6,82 | 2,77 | 0,003042436 |
| Q9BZQ4 | Nicotinamide/nicotinic acid mononucleotide adenylyltransferase 2 [OS=Homo sapiens] | NMNAT2 | 6,957 | 2,8 | 0,002742856 |
| Q8TAZ6 | CKLF-like MARVEL transmembrane domain-containing protein 2 [OS=Homo sapiens] | CMTM2 | 7,279 | 2,86 | 0,00215984 |
| P07205 | Phosphoglycerate kinase 2 [OS=Homo sapiens] | PGK2 | 7,397 | 2,89 | 0,00198252 |
| Q9Y2Q3 | Glutathione S-transferase kappa 1 [OS=Homo sapiens] | GSTK1 | 7,658 | 2,94 | 0,001643259 |
| Q8NFJ9-2 | Isoform 3 of Bardet-Biedl syndrome 1 protein [OS=Homo sapiens] | BBS1 | 7,985 | 3 | 0,001305312 |
| Q96PQ0 | VPS10 domain-containing receptor SorCS2 [OS=Homo sapiens] | SORCS2 | 9,989 | 3,32 | 0,000354706 |
| Q14978 | Nucleolar and coiled-body phosphoprotein 1 [OS=Homo sapiens] | NOLC1 | 10,088 | 3,33 | 0,000334037 |
| Q96EZ8-3 | Isoform 3 of Microspherule protein 1 [OS=Homo sapiens] | MCRS1 | 10,477 | 3,39 | 0,000264685 |
| Q8N878 | FERM domain-containing protein 1 [OS=Homo sapiens] | FRMD1 | 12,131 | 3,6 | 0,000103897 |
| Q9NWW7 | Uncharacterized protein C2orf42 [OS=Homo sapiens] | C2orf42 | 27,063 | 4,76 | 2,46728E-07 |
| A8MTZ0 | BBSome-interacting protein 1 [OS=Homo sapiens] | BBIP1 | 38,794 | 5,28 | 9,80459E-09 |
| P49796-2 | Isoform 2 of Regulator of G-protein signaling 3 [OS=Homo sapiens] | RGS3 | 100 | 6,64 | 1E-17 |
| Q9Y4R7-6 | Isoform 4 of Tubulin monoglycylase TTLL3 [OS=Homo sapiens] | TTLL3 | 100 | 6,64 | 1E-17 |
| Q8N859 | Zinc finger protein 713 [OS=Homo sapiens] | ZNF713 | 100 | 6,64 | 1E-17 |
| Q96K58-2 | Isoform 2 of Zinc finger protein 668 [OS=Homo sapiens] | TP53 | 100 | 6,64 | 1E-17 |
| Q5SYB0-2 | Isoform 2 of FERM and PDZ domain-containing protein 1 [OS=Homo sapiens] | FRMPD1 | 100 | 6,64 | 1E-17 |
| Q9NX31 | Oxidative stress-responsive serine-rich protein 1 [OS=Homo sapiens] | OSER1 | 100 | 6,64 | 1E-17 |
| Q8NEX6 | Protein WFDC11 [OS=Homo sapiens] | WFDC11 | 100 | 6,64 | 1E-17 |
| Q16555 | Dihydropyrimidinase-related protein 2 [OS=Homo sapiens] | DPYSL2 | 100 | 6,64 | 1E-17 |
| Q96DT6 | Cysteine protease ATG4C [OS=Homo sapiens] | ATG4C | 100 | 6,64 | 1E-17 |
| O75323 | Protein NipSnap homolog 2 [OS=Homo sapiens] | NIPSNAP2 | 100 | 6,64 | 1E-17 |
| O43566 | Regulator of G-protein signaling 14 [OS=Homo sapiens] | RGS14 | 100 | 6,64 | 1E-17 |
| A9YTQ3-3 | Isoform 3 of Aryl hydrocarbon receptor repressor [OS=Homo sapiens] | AHRR | 100 | 6,64 | 1E-17 |
| Q9NWY4 | Histone PARylation factor 1 [OS=Homo sapiens] | HPF1 | 100 | 6,64 | 1E-17 |
| P09104 | Gamma-enolase [OS=Homo sapiens] | ENO2 | 100 | 6,64 | 1E-17 |
| P51172-2 | Isoform 2 of Amiloride-sensitive sodium channel subunit delta [OS=Homo sapiens] | SCNN1D | 100 | 6,64 | 1E-17 |
| Q8TE54 | Anion exchange transporter [OS=Homo sapiens] | SLC26A7 | 100 | 6,64 | 1E-17 |
| P36776-3 | Isoform 3 of Lon protease homolog, mitochondrial [OS=Homo sapiens] | LONP1 | 100 | 6,64 | 1E-17 |
| Q9H0Q0 | CYFIP-related Rac1 interactor A [OS=Homo sapiens] | CYRIA | 100 | 6,64 | 1E-17 |
| Q9BPX1 | 17-beta-hydroxysteroid dehydrogenase 14 [OS=Homo sapiens] | HSD17B14 | 100 | 6,64 | 1E-17 |
| Q8IWT0 | Protein archease [OS=Homo sapiens] | ZBTB8OS | 100 | 6,64 | 1E-17 |
| Q8NE28-3 | Isoform 3 of Serine/threonine kinase-like domain-containing protein STKLD1 [OS=Homo sapiens] | STKLD1 | 100 | 6,64 | 1E-17 |
| Q9Y219 | Protein jagged-2 [OS=Homo sapiens] | JAG2 | 100 | 6,64 | 1E-17 |
| O14526-3 | Isoform 3 of F-BAR domain only protein 1 [OS=Homo sapiens] | FCHO1 | 100 | 6,64 | 1E-17 |
| P00167 | Cytochrome b5 [OS=Homo sapiens] | CYB5A | 100 | 6,64 | 1E-17 |
| Q7Z5Y7-3 | Isoform 3 of BTB/POZ domain-containing protein KCTD20 [OS=Homo sapiens] | KCTD20 | 100 | 6,64 | 1E-17 |
| Q9BTD8 | RNA-binding protein 42 [OS=Homo sapiens] | RBM42 | 100 | 6,64 | 1E-17 |
| B3EWG6 | Protein FAM25G [OS=Homo sapiens] | FAM25G | 100 | 6,64 | 1E-17 |
| Q8IUI8 | Cytokine receptor-like factor 3 [OS=Homo sapiens] | CRLF3 | 100 | 6,64 | 1E-17 |
| Q9Y237 | Peptidyl-prolyl cis-trans isomerase NIMA-interacting 4 [OS=Homo sapiens] | PIN4 | 100 | 6,64 | 1E-17 |
| Q8WVY7 | Ubiquitin-like domain-containing CTD phosphatase 1 [OS=Homo sapiens] | UBLCP1 | 100 | 6,64 | 1E-17 |
| O15212 | Prefoldin subunit 6 [OS=Homo sapiens] | PFDN6 | 100 | 6,64 | 1E-17 |
| Q2TAZ0-4 | Isoform 3 of Autophagy-related protein 2 homolog A [OS=Homo sapiens] | ATG2A | 100 | 6,64 | 1E-17 |

### B) Rivaroxaban

| Accession | Description | Gene Symbol | Abundance Ratio | Abundance Ratio (log2) | Abundance Ratio P-Value |
| --- | --- | --- | --- | --- | --- |
| P54845 | Neural retina-specific leucine zipper protein [OS=Homo sapiens] | NRL | 0,01 | -6,64 | 1E-17 |
| Q5T7W7 | Thiosulfate sulfurtransferase/rhodanese-like domain-containing protein 2 [OS=Homo sapiens] | TSTD2 | 0,01 | -6,64 | 1E-17 |
| O75952-6 | Isoform 6 of Calcium-binding tyrosine phosphorylation-regulated protein [OS=Homo sapiens] | CABYR | 0,01 | -6,64 | 1E-17 |
| A2RU67 | Protein FAM234B [OS=Homo sapiens] | FAM234B | 0,01 | -6,64 | 1E-17 |
| Q13405 | 39S ribosomal protein L49, mitochondrial [OS=Homo sapiens] | MRPL49 | 0,01 | -6,64 | 1E-17 |
| P22090 | 40S ribosomal protein S4, Y isoform 1 [OS=Homo sapiens] | RPS4Y1 | 0,01 | -6,64 | 1E-17 |
| Q5VWQ8 | Disabled homolog 2-interacting protein [OS=Homo sapiens] | DAB2IP | 0,01 | -6,64 | 1E-17 |
| Q9H2C1 | LIM/homeobox protein Lhx5 [OS=Homo sapiens] | LHX5 | 0,01 | -6,64 | 1E-17 |
| Q8N584-2 | Isoform 2 of Tetratricopeptide repeat protein 39C [OS=Homo sapiens] | TTC39C | 0,01 | -6,64 | 1E-17 |
| O96028-2 | Isoform 2 of Histone-lysine N-methyltransferase NSD2 [OS=Homo sapiens] | NSD2 | 0,01 | -6,64 | 1E-17 |
| P31415 | Calsequestrin-1 [OS=Homo sapiens] | CASQ1 | 0,01 | -6,64 | 1E-17 |
| A9UHW6 | MIF4G domain-containing protein [OS=Homo sapiens] | MIF4GD | 0,01 | -6,64 | 1E-17 |
| Q15257 | Serine/threonine-protein phosphatase 2A activator [OS=Homo sapiens] | PTPA | 0,01 | -6,64 | 1E-17 |
| Q8N729 | Neuropeptide W [OS=Homo sapiens] | NPW | 0,01 | -6,64 | 1E-17 |
| P22749-2 | Isoform Short of Granulysin [OS=Homo sapiens] | GNLY | 0,01 | -6,64 | 1E-17 |
| Q9NSK0-3 | Isoform 3 of Kinesin light chain 4 [OS=Homo sapiens] | KLC4 | 0,01 | -6,64 | 1E-17 |
| Q9UBL6 | Copine-7 [OS=Homo sapiens] | CPNE7 | 0,01 | -6,64 | 1E-17 |
| Q5XG92-6 | Isoform 5 of Carboxylesterase 4A [OS=Homo sapiens] | CES4A | 0,01 | -6,64 | 1E-17 |
| P21754 | Zona pellucida sperm-binding protein 3 [OS=Homo sapiens] | ZP3 | 0,01 | -6,64 | 1E-17 |
| Q9UJY5-3 | Isoform 3 of ADP-ribosylation factor-binding protein GGA1 [OS=Homo sapiens] | GGA1 | 0,01 | -6,64 | 1E-17 |
| O95563 | Mitochondrial pyruvate carrier 2 [OS=Homo sapiens] | MPC2 | 0,01 | -6,64 | 1E-17 |
| Q16585 | Beta-sarcoglycan [OS=Homo sapiens] | SGCB | 0,01 | -6,64 | 1E-17 |
| Q8TC92-2 | Isoform 2 of Ecto-NOX disulfide-thiol exchanger 1 [OS=Homo sapiens] | ENOX1 | 0,01 | -6,64 | 1E-17 |
| Q9UIY3 | RWD domain-containing protein 2A [OS=Homo sapiens] | RWDD2A | 0,01 | -6,64 | 1E-17 |
| Q9H825-2 | Isoform 2 of mRNA N(3)-methylcytidine methyltransferase METTL8 [OS=Homo sapiens] | METTL8 | 0,01 | -6,64 | 1E-17 |
| P51172-1 | Isoform 1 of Amiloride-sensitive sodium channel subunit delta [OS=Homo sapiens] | SCNN1D | 0,01 | -6,64 | 1E-17 |
| Q5EBL8-2 | Isoform 2 of PDZ domain-containing protein 11 [OS=Homo sapiens] | PDZD11 | 0,01 | -6,64 | 1E-17 |
| P15692-14 | Isoform L-VEGF206 of Vascular endothelial growth factor A [OS=Homo sapiens] | VEGFA | 0,01 | -6,64 | 1E-17 |
| P61371 | Insulin gene enhancer protein ISL-1 [OS=Homo sapiens] | ISL1 | 0,01 | -6,64 | 1E-17 |
| Q9H172-3 | Isoform 3 of ATP-binding cassette sub-family G member 4 [OS=Homo sapiens] | ABCG4 | 0,01 | -6,64 | 1E-17 |
| Q5JQC9-2 | Isoform 2 of A-kinase anchor protein 4 [OS=Homo sapiens] | AKAP4 | 0,01 | -6,64 | 1E-17 |
| Q02045 | Myosin light chain 5 [OS=Homo sapiens] | MYL5 | 0,01 | -6,64 | 1E-17 |
| O14490-4 | Isoform 4 of Disks large-associated protein 1 [OS=Homo sapiens] | DLGAP1 | 0,01 | -6,64 | 1E-17 |
| Q03989 | AT-rich interactive domain-containing protein 5A [OS=Homo sapiens] | ARID5A | 0,01 | -6,64 | 1E-17 |
| O94827-6 | Isoform 6 of Pleckstrin homology domain-containing family G member 5 [OS=Homo sapiens] | PLEKHG5 | 0,01 | -6,64 | 1E-17 |
| Q9UNX9 | ATP-sensitive inward rectifier potassium channel 14 [OS=Homo sapiens] | KCNJ14 | 0,01 | -6,64 | 1E-17 |
| Q13938-4 | Isoform 3 of Calcyphosin [OS=Homo sapiens] | CAPS | 0,01 | -6,64 | 1E-17 |
| A6NJZ3 | Olfactory receptor 6C65 [OS=Homo sapiens] | OR6C65 | 0,01 | -6,64 | 1E-17 |
| Q8IYP9 | Palmitoyltransferase ZDHHC23 [OS=Homo sapiens] | ZDHHC23 | 0,013 | -6,28 | 1,22041E-10 |
| Q6AWC2-2 | Isoform 2 of Protein WWC2 [OS=Homo sapiens] | WWC2 | 0,024 | -5,36 | 4,83003E-08 |
| Q96Q15-3 | Isoform 3 of Serine/threonine-protein kinase SMG1 [OS=Homo sapiens] | SMG1 | 0,025 | -5,31 | 4,87604E-08 |
| Q8NFM7-2 | Isoform 2 of Interleukin-17 receptor D [OS=Homo sapiens] | IL17RD | 0,028 | -5,18 | 1,03586E-07 |
| Q8NCK7 | Monocarboxylate transporter 11 [OS=Homo sapiens] | SLC16A11 | 0,029 | -5,13 | 1,3566E-07 |
| O75177 | Calcium-responsive transactivator [OS=Homo sapiens] | SS18L1 | 0,03 | -5,07 | 1,90659E-07 |
| O00142 | Thymidine kinase 2, mitochondrial [OS=Homo sapiens] | TK2 | 0,032 | -4,97 | 3,28494E-07 |
| Q5JUX0 | Spindlin-3 [OS=Homo sapiens] | SPIN3 | 0,038 | -4,73 | 1,10727E-06 |
| Q9BSQ5-2 | Isoform 2 of Cerebral cavernous malformations 2 protein [OS=Homo sapiens] | CCM2 | 0,041 | -4,61 | 2,06896E-06 |
| P27037 | Activin receptor type-2A [OS=Homo sapiens] | ACVR2A | 0,045 | -4,47 | 4,19711E-06 |
| P48507 | Glutamate--cysteine ligase regulatory subunit [OS=Homo sapiens] | GCLM | 0,05 | -4,31 | 3,61905E-05 |
| Q719H9 | BTB/POZ domain-containing protein KCTD1 [OS=Homo sapiens] | KCTD1 | 0,055 | -4,19 | 1,56888E-05 |
| Q9BRS8 | La-related protein 6 [OS=Homo sapiens] | LARP6 | 0,056 | -4,15 | 1,83847E-05 |
| Q6P996-3 | Isoform 3 of Pyridoxal-dependent decarboxylase domain-containing protein 1 [OS=Homo sapiens] | PDXDC1 | 0,06 | -4,06 | 0,000119259 |
| Q96PG2 | Membrane-spanning 4-domains subfamily A member 10 [OS=Homo sapiens] | MS4A10 | 0,066 | -3,93 | 4,98947E-05 |
| P01034 | Cystatin-C [OS=Homo sapiens] | CST3 | 0,066 | -3,92 | 0,000188617 |
| Q9H9T3-2 | Isoform 2 of Elongator complex protein 3 [OS=Homo sapiens] | ELP3 | 0,068 | -3,87 | 6,42483E-05 |
| O95382-2 | Isoform 2 of Mitogen-activated protein kinase kinase kinase 6 [OS=Homo sapiens] | MAP3K6 | 0,084 | -3,57 | 0,000225919 |
| Q86Y07-3 | Isoform 3 of Serine/threonine-protein kinase VRK2 [OS=Homo sapiens] | VRK2 | 0,094 | -3,42 | 0,000404319 |
| P32019-3 | Isoform 3 of Type II inositol 1,4,5-trisphosphate 5-phosphatase [OS=Homo sapiens] | INPP5B | 0,101 | -3,31 | 0,0006113 |
| Q86TL0-2 | Isoform 2 of Cysteine protease ATG4D [OS=Homo sapiens] | ATG4D | 0,126 | -2,99 | 0,005233515 |
| Q6UWN5 | Ly6/PLAUR domain-containing protein 5 [OS=Homo sapiens] | LYPD5 | 0,128 | -2,96 | 0,002086099 |
| Q03013 | Glutathione S-transferase Mu 4 [OS=Homo sapiens] | GSTM4 | 0,131 | -2,93 | 0,002333596 |
| Q5SRD1 | Mitochondrial import inner membrane translocase subunit Tim23B OS=Homo sapiens OX=9606 GN=TIMM23B PE=1 SV=3 | TIMM23B | 0,137 | -2,87 | 0,002897523 |
| Q8WU43 | Uncharacterized protein C2orf15 [OS=Homo sapiens] | C2orf15 | 0,138 | -2,85 | 0,003026098 |
| Q14005 | Pro-interleukin-16 [OS=Homo sapiens] | IL16 | 0,139 | -2,84 | 0,007808262 |
| P57729 | Ras-related protein Rab-38 [OS=Homo sapiens] | RAB38 | 0,165 | -2,6 | 0,013281744 |
| O00305-3 | Isoform 3 of Voltage-dependent L-type calcium channel subunit beta-4 [OS=Homo sapiens] | CACNB4 | 0,175 | -2,52 | 0,008729988 |
| Q3T906 | N-acetylglucosamine-1-phosphotransferase subunits alpha/beta [OS=Homo sapiens] | GNPTAB | 0,18 | -2,47 | 0,009889577 |
| Q69YI7 | Nuclear apoptosis-inducing factor 1 [OS=Homo sapiens] | NAIF1 | 0,182 | -2,46 | 0,010263803 |
| P11230 | Acetylcholine receptor subunit beta [OS=Homo sapiens] | CHRNB1 | 0,194 | -2,37 | 0,013391358 |
| Q9UJ70-2 | Isoform 2 of N-acetyl-D-glucosamine kinase [OS=Homo sapiens] | NAGK | 0,214 | -2,22 | 0,019958411 |
| Q9BV57-2 | Isoform 2 of 1,2-dihydroxy-3-keto-5-methylthiopentene dioxygenase [OS=Homo sapiens] | ADI1 | 0,215 | -2,22 | 0,020183209 |
| Q96MU8 | Kremen protein 1 [OS=Homo sapiens] | KREMEN1 | 0,234 | -2,1 | 0,027965554 |
| Q5HYR2 | Doublesex- and mab-3-related transcription factor C1 [OS=Homo sapiens] | DMRTC1; DMRTC1B | 0,238 | -2,07 | 0,03009243 |
| Q9UKV3-2 | Isoform 2 of Apoptotic chromatin condensation inducer in the nucleus [OS=Homo sapiens] | ACIN1 | 0,247 | -2,02 | 0,035365698 |
| P36575 | Arrestin-C [OS=Homo sapiens] | ARR3 | 0,256 | -1,96 | 0,039178392 |
| O95990-4 | Isoform 4 of Actin-associated protein FAM107A [OS=Homo sapiens] | FAM107A | 0,257 | -1,96 | 0,039625812 |
| Q96PM5-6 | Isoform 6 of RING finger and CHY zinc finger domain-containing protein 1 [OS=Homo sapiens] | RCHY1 | 0,257 | -1,96 | 0,039460652 |
| Q9NZM6 | Polycystic kidney disease 2-like 2 protein [OS=Homo sapiens] | PKD2L2 | 0,259 | -1,95 | 0,042852782 |
| Q96SR6-2 | Isoform 2 of Zinc finger protein 382 [OS=Homo sapiens] | ZNF382 | 0,263 | -1,93 | 0,045886453 |
| Q9NU63 | Zinc finger protein 57 homolog [OS=Homo sapiens] | ZFP57 | 0,268 | -1,9 | 0,045753386 |
| O60809 | PRAME family member 10 [OS=Homo sapiens] | PRAMEF10 | 4,048 | 2,02 | 0,048490285 |
| P45877 | Peptidyl-prolyl cis-trans isomerase C [OS=Homo sapiens] | PPIC | 4,076 | 2,03 | 0,047362081 |
| P11137 | Microtubule-associated protein 2 [OS=Homo sapiens] | MAP2 | 4,176 | 2,06 | 0,043509426 |
| Q96JE7 | Protein transport protein Sec16B [OS=Homo sapiens] | SEC16B | 4,177 | 2,06 | 0,043474336 |
| Q8IXM2 | Chromatin complexes subunit BAP18 [OS=Homo sapiens] | BACC1 | 4,226 | 2,08 | 0,041751792 |
| Q86VX9-5 | Isoform 5 of Vacuolar fusion protein MON1 homolog A [OS=Homo sapiens] | MON1A | 4,582 | 2,2 | 0,031199604 |
| A6NJY4 | Putative transmembrane protein LOC100289255 [OS=Homo sapiens] | TMEM238L | 4,628 | 2,21 | 0,049004701 |
| Q8IXL7-2 | Isoform 2 of Methionine-R-sulfoxide reductase B3 [OS=Homo sapiens] | MSRB3 | 4,64 | 2,21 | 0,029808031 |
| Q8N3J6-5 | Isoform 6 of Cell adhesion molecule 2 [OS=Homo sapiens] | CADM2 | 4,653 | 2,22 | 0,049129439 |
| Q5U5R9 | Probable E3 ubiquitin-protein ligase HECTD2 [OS=Homo sapiens] | HECTD2 | 4,658 | 2,22 | 0,029377581 |
| A0A0G2JND7 | Uncharacterized protein OS=Homo sapiens OX=9606 PE=4 SV=1 |  | 4,674 | 2,22 | 0,029002425 |
| Q9NPC1 | Leukotriene B4 receptor 2 [OS=Homo sapiens] | LTB4R2 | 4,705 | 2,23 | 0,028301014 |
| Q9H6Z9 | Prolyl hydroxylase EGLN3 [OS=Homo sapiens] | EGLN3 | 4,892 | 2,29 | 0,043367651 |
| Q969R2 | Oxysterol-binding protein 2 [OS=Homo sapiens] | OSBP2 | 4,906 | 2,29 | 0,024170013 |
| Q9NYP8 | Uncharacterized protein C21orf62 [OS=Homo sapiens] | C21orf62 | 4,922 | 2,3 | 0,023872717 |
| Q8NG35 | Beta-defensin 105 [OS=Homo sapiens] | DEFB105A; DEFB105B | 4,956 | 2,31 | 0,045817965 |
| Q6PJT7-4 | Isoform 4 of Zinc finger CCCH domain-containing protein 14 [OS=Homo sapiens] | ZC3H14 | 5,063 | 2,34 | 0,021429335 |
| Q16613-2 | Isoform 2 of Serotonin N-acetyltransferase [OS=Homo sapiens] | AANAT | 5,075 | 2,34 | 0,021224436 |
| Q14978 | Nucleolar and coiled-body phosphoprotein 1 [OS=Homo sapiens] | NOLC1 | 5,081 | 2,35 | 0,021128448 |
| Q32NC0-2 | Isoform 2 of UPF0711 protein C18orf21 [OS=Homo sapiens] | C18orf21 | 5,403 | 2,43 | 0,016594762 |
| Q01543-2 | Isoform 2 of Friend leukemia integration 1 transcription factor [OS=Homo sapiens] | FLI1 | 5,44 | 2,44 | 0,016151149 |
| Q9P107 | GEM-interacting protein [OS=Homo sapiens] | GMIP | 5,493 | 2,46 | 0,015537989 |
| Q7Z5A7 | Chemokine-like protein TAFA-5 [OS=Homo sapiens] | TAFA5 | 5,635 | 2,49 | 0,014009063 |
| Q9HB75-6 | Isoform 6 of p53-induced death domain-containing protein 1 [OS=Homo sapiens] | PIDD1 | 5,926 | 2,57 | 0,011388608 |
| P0CB38 | Polyadenylate-binding protein 4-like [OS=Homo sapiens] | PABPC4L | 6,029 | 2,59 | 0,019942254 |
| Q96DZ9 | CKLF-like MARVEL transmembrane domain-containing protein 5 [OS=Homo sapiens] | CMTM5 | 6,193 | 2,63 | 0,009456707 |
| Q96FC9-3 | Isoform 3 of ATP-dependent DNA helicase DDX11 [OS=Homo sapiens] | DDX11 | 6,252 | 2,64 | 0,014248254 |
| Q6UWP2 | Dehydrogenase/reductase SDR family member 11 [OS=Homo sapiens] | DHRS11 | 6,261 | 2,65 | 0,00902548 |
| P46100 | Transcriptional regulator ATRX [OS=Homo sapiens] | ATRX | 6,531 | 2,71 | 0,007523474 |
| O75185 | Calcium-transporting ATPase type 2C member 2 [OS=Homo sapiens] | ATP2C2 | 6,596 | 2,72 | 0,007203727 |
| Q8N878 | FERM domain-containing protein 1 [OS=Homo sapiens] | FRMD1 | 6,695 | 2,74 | 0,014095962 |
| O43155 | Leucine-rich repeat transmembrane protein FLRT2 [OS=Homo sapiens] | FLRT2 | 6,901 | 2,79 | 0,00815059 |
| P07205 | Phosphoglycerate kinase 2 [OS=Homo sapiens] | PGK2 | 6,994 | 2,81 | 0,005555233 |
| Q96N06-2 | Isoform 2 of Spermatogenesis-associated protein 33 [OS=Homo sapiens] | SPATA33 | 7,591 | 2,92 | 0,003819486 |
| O75157 | TSC22 domain family protein 2 [OS=Homo sapiens] | TSC22D2 | 7,644 | 2,93 | 0,003696176 |
| Q9UFH2 | Dynein heavy chain 17, axonemal [OS=Homo sapiens] | DNAH17 | 7,778 | 2,96 | 0,005539305 |
| Q96B26 | Exosome complex component RRP43 [OS=Homo sapiens] | EXOSC8 | 8,446 | 3,08 | 0,007100759 |
| Q9Y2Q3 | Glutathione S-transferase kappa 1 [OS=Homo sapiens] | GSTK1 | 9,356 | 3,23 | 0,003537666 |
| Q6ZMM2 | ADAMTS-like protein 5 [OS=Homo sapiens] | ADAMTSL5 | 10,311 | 3,37 | 0,002896858 |
| Q96EZ8-3 | Isoform 3 of Microspherule protein 1 [OS=Homo sapiens] | MCRS1 | 10,855 | 3,44 | 0,000635543 |
| Q8NDI1 | EH domain-binding protein 1 [OS=Homo sapiens] | EHBP1 | 11,67 | 3,54 | 0,000428707 |
| Q9H425 | Uncharacterized protein C1orf198 [OS=Homo sapiens] | C1orf198 | 11,943 | 3,58 | 0,000377038 |
| Q96PQ0 | VPS10 domain-containing receptor SorCS2 [OS=Homo sapiens] | SORCS2 | 12,289 | 3,62 | 0,000321465 |
| Q8NFF5 | FAD synthase [OS=Homo sapiens] | FLAD1 | 15,928 | 3,99 | 6,98433E-05 |
| Q13439 | Golgin subfamily A member 4 [OS=Homo sapiens] | GOLGA4 | 24,154 | 4,59 | 2,27409E-05 |
| Q9BZF9 | Uveal autoantigen with coiled-coil domains and ankyrin repeats [OS=Homo sapiens] | UACA | 24,708 | 4,63 | 2,42185E-05 |
| Q8NFJ9-2 | Isoform 3 of Bardet-Biedl syndrome 1 protein [OS=Homo sapiens] | BBS1 | 25,597 | 4,68 | 2,39133E-05 |
| Q9NWW7 | Uncharacterized protein C2orf42 [OS=Homo sapiens] | C2orf42 | 26,997 | 4,75 | 2,04881E-06 |
| O43300 | Leucine-rich repeat transmembrane neuronal protein 2 [OS=Homo sapiens] | LRRTM2 | 27,217 | 4,77 | 1,37827E-05 |
| P49796-2 | Isoform 2 of Regulator of G-protein signaling 3 [OS=Homo sapiens] | RGS3 | 100 | 6,64 | 1E-17 |
| Q9Y4R7-6 | Isoform 4 of Tubulin monoglycylase TTLL3 [OS=Homo sapiens] | TTLL3 | 100 | 6,64 | 1E-17 |
| Q9H0T7 | Ras-related protein Rab-17 [OS=Homo sapiens] | RAB17 | 100 | 6,64 | 1E-17 |
| Q9H0E9 | Bromodomain-containing protein 8 [OS=Homo sapiens] | BRD8 | 100 | 6,64 | 1E-17 |
| P48544 | G protein-activated inward rectifier potassium channel 4 [OS=Homo sapiens] | KCNJ5 | 100 | 6,64 | 1E-17 |
| Q8N859 | Zinc finger protein 713 [OS=Homo sapiens] | ZNF713 | 100 | 6,64 | 1E-17 |
| O75896 | Tumor suppressor candidate 2 [OS=Homo sapiens] | TUSC2 | 100 | 6,64 | 1E-17 |
| Q53GS9 | U4/U6.U5 tri-snRNP-associated protein 2 [OS=Homo sapiens] | USP39 | 100 | 6,64 | 1E-17 |
| Q96K58-2 | Isoform 2 of Zinc finger protein 668 [OS=Homo sapiens] | TP53 | 100 | 6,64 | 1E-17 |
| Q5SYB0-2 | Isoform 2 of FERM and PDZ domain-containing protein 1 [OS=Homo sapiens] | FRMPD1 | 100 | 6,64 | 1E-17 |
| Q9NX31 | Oxidative stress-responsive serine-rich protein 1 [OS=Homo sapiens] | OSER1 | 100 | 6,64 | 1E-17 |
| Q8NEX6 | Protein WFDC11 [OS=Homo sapiens] | WFDC11 | 100 | 6,64 | 1E-17 |
| A0A590UK24 | Uncharacterized protein OS=Homo sapiens OX=9606 GN=LOC122319696 PE=4 SV=1 | LOC122319696 | 100 | 6,64 | 1E-17 |
| P60153-2 | Isoform 2 of Inactive ribonuclease-like protein 9 [OS=Homo sapiens] | RNASE9 | 100 | 6,64 | 1E-17 |
| Q6ZYL4 | General transcription factor IIH subunit 5 [OS=Homo sapiens] | GTF2H5 | 100 | 6,64 | 1E-17 |
| O95922 | Probable tubulin polyglutamylase TTLL1 [OS=Homo sapiens] | TTLL1 | 100 | 6,64 | 1E-17 |
| O75052 | Carboxyl-terminal PDZ ligand of neuronal nitric oxide synthase protein [OS=Homo sapiens] | NOS1AP | 100 | 6,64 | 1E-17 |
| Q16555 | Dihydropyrimidinase-related protein 2 [OS=Homo sapiens] | DPYSL2 | 100 | 6,64 | 1E-17 |
| Q5VT52 | Regulation of nuclear pre-mRNA domain-containing protein 2 [OS=Homo sapiens] | RPRD2 | 100 | 6,64 | 1E-17 |
| P43246 | DNA mismatch repair protein Msh2 [OS=Homo sapiens] | MSH2 | 100 | 6,64 | 1E-17 |
| Q7L2E3-2 | Isoform 2 of ATP-dependent RNA helicase DHX30 [OS=Homo sapiens] | DHX30 | 100 | 6,64 | 1E-17 |
| Q96DT6 | Cysteine protease ATG4C [OS=Homo sapiens] | ATG4C | 100 | 6,64 | 1E-17 |
| O75323 | Protein NipSnap homolog 2 [OS=Homo sapiens] | NIPSNAP2 | 100 | 6,64 | 1E-17 |
| Q8N335 | Glycerol-3-phosphate dehydrogenase 1-like protein [OS=Homo sapiens] | GPD1L | 100 | 6,64 | 1E-17 |
| O00584 | Ribonuclease T2 [OS=Homo sapiens] | RNASET2 | 100 | 6,64 | 1E-17 |
| O75533 | Splicing factor 3B subunit 1 [OS=Homo sapiens] | SF3B1 | 100 | 6,64 | 1E-17 |
| O43566 | Regulator of G-protein signaling 14 [OS=Homo sapiens] | RGS14 | 100 | 6,64 | 1E-17 |
| Q8TDN6 | Ribosome biogenesis protein BRX1 homolog [OS=Homo sapiens] | BRIX1 | 100 | 6,64 | 1E-17 |
| Q9NWY4 | Histone PARylation factor 1 [OS=Homo sapiens] | HPF1 | 100 | 6,64 | 1E-17 |
| Q8TE54 | Anion exchange transporter [OS=Homo sapiens] | SLC26A7 | 100 | 6,64 | 1E-17 |
| P07954-2 | Isoform Cytoplasmic of Fumarate hydratase, mitochondrial [OS=Homo sapiens] | FH | 100 | 6,64 | 1E-17 |
| P36776-3 | Isoform 3 of Lon protease homolog, mitochondrial [OS=Homo sapiens] | LONP1 | 100 | 6,64 | 1E-17 |
| O14782 | Kinesin-like protein KIF3C [OS=Homo sapiens] | KIF3C | 100 | 6,64 | 1E-17 |
| Q9H0Q0 | CYFIP-related Rac1 interactor A [OS=Homo sapiens] | CYRIA | 100 | 6,64 | 1E-17 |
| Q8NGI7 | Olfactory receptor 10V1 [OS=Homo sapiens] | OR10V1 | 100 | 6,64 | 1E-17 |
| Q8IWT0 | Protein archease [OS=Homo sapiens] | ZBTB8OS | 100 | 6,64 | 1E-17 |
| P17858 | ATP-dependent 6-phosphofructokinase, liver type [OS=Homo sapiens] | PFKL | 100 | 6,64 | 1E-17 |
| Q8NE28-3 | Isoform 3 of Serine/threonine kinase-like domain-containing protein STKLD1 [OS=Homo sapiens] | STKLD1 | 100 | 6,64 | 1E-17 |
| Q02241 | Kinesin-like protein KIF23 [OS=Homo sapiens] | KIF23 | 100 | 6,64 | 1E-17 |
| O14526-3 | Isoform 3 of F-BAR domain only protein 1 [OS=Homo sapiens] | FCHO1 | 100 | 6,64 | 1E-17 |
| P00167 | Cytochrome b5 [OS=Homo sapiens] | CYB5A | 100 | 6,64 | 1E-17 |
| Q8N4G2 | ADP-ribosylation factor-like protein 14 [OS=Homo sapiens] | ARL14 | 100 | 6,64 | 1E-17 |
| Q7Z5Y7-3 | Isoform 3 of BTB/POZ domain-containing protein KCTD20 [OS=Homo sapiens] | KCTD20 | 100 | 6,64 | 1E-17 |
| P06850 | Corticoliberin [OS=Homo sapiens] | CRH | 100 | 6,64 | 1E-17 |
| Q9BTD8 | RNA-binding protein 42 [OS=Homo sapiens] | RBM42 | 100 | 6,64 | 1E-17 |
| Q9HB58-4 | Isoform 4 of Sp110 nuclear body protein [OS=Homo sapiens] | SP110 | 100 | 6,64 | 1E-17 |
| B3EWG6 | Protein FAM25G [OS=Homo sapiens] | FAM25G | 100 | 6,64 | 1E-17 |
| P31641 | Sodium- and chloride-dependent taurine transporter [OS=Homo sapiens] | SLC6A6 | 100 | 6,64 | 1E-17 |
| Q9NV64 | Transmembrane protein 39A [OS=Homo sapiens] | TMEM39A | 100 | 6,64 | 1E-17 |
| Q15596 | Nuclear receptor coactivator 2 [OS=Homo sapiens] | NCOA2 | 100 | 6,64 | 1E-17 |
| Q8IUI8 | Cytokine receptor-like factor 3 [OS=Homo sapiens] | CRLF3 | 100 | 6,64 | 1E-17 |
| Q9Y237 | Peptidyl-prolyl cis-trans isomerase NIMA-interacting 4 [OS=Homo sapiens] | PIN4 | 100 | 6,64 | 1E-17 |
| Q8WVY7 | Ubiquitin-like domain-containing CTD phosphatase 1 [OS=Homo sapiens] | UBLCP1 | 100 | 6,64 | 1E-17 |
| O15212 | Prefoldin subunit 6 [OS=Homo sapiens] | PFDN6 | 100 | 6,64 | 1E-17 |
| O76027 | Annexin A9 [OS=Homo sapiens] | ANXA9 | 100 | 6,64 | 1E-17 |
| Q2TAZ0-4 | Isoform 3 of Autophagy-related protein 2 homolog A [OS=Homo sapiens] | ATG2A | 100 | 6,64 | 1E-17 |
| P60604 | Ubiquitin-conjugating enzyme E2 G2 [OS=Homo sapiens] | UBE2G2 | 100 | 6,64 | 1E-17 |

### C) Edoxaban

| Accession | Description | Gene Symbol | Abundance Ratio | Abundance Ratio (log2) | Abundance Ratio P-Value |
| --- | --- | --- | --- | --- | --- |
| Q5T124-8 | Isoform 8 of UBX domain-containing protein 11 [OS=Homo sapiens] | UBXN11 | 0,01 | -6,64 | 1E-17 |
| Q05682-3 | Isoform 3 of Caldesmon [OS=Homo sapiens] | CALD1 | 0,01 | -6,64 | 1E-17 |
| Q15648 | Mediator of RNA polymerase II transcription subunit 1 [OS=Homo sapiens] | MED1 | 0,01 | -6,64 | 1E-17 |
| O15527 | N-glycosylase/DNA lyase [OS=Homo sapiens] | OGG1 | 0,01 | -6,64 | 1E-17 |
| O75952-6 | Isoform 6 of Calcium-binding tyrosine phosphorylation-regulated protein [OS=Homo sapiens] | CABYR | 0,01 | -6,64 | 1E-17 |
| A6NEH8 | Putative uncharacterized protein encoded by ZNF503-AS2 [OS=Homo sapiens] | ZNF503-AS2 | 0,01 | -6,64 | 1E-17 |
| Q96PE3 | Inositol polyphosphate-4-phosphatase type I A [OS=Homo sapiens] | INPP4A | 0,01 | -6,64 | 1E-17 |
| Q12765 | Secernin-1 [OS=Homo sapiens] | SCRN1 | 0,01 | -6,64 | 1E-17 |
| Q8NB12 | Histone-lysine N-methyltransferase SMYD1 [OS=Homo sapiens] | SMYD1 | 0,01 | -6,64 | 1E-17 |
| Q5VZ52 | MORN repeat-containing protein 5 [OS=Homo sapiens] | MORN5 | 0,01 | -6,64 | 1E-17 |
| Q8N584-2 | Isoform 2 of Tetratricopeptide repeat protein 39C [OS=Homo sapiens] | TTC39C | 0,01 | -6,64 | 1E-17 |
| Q2VPA4-2 | Isoform 2 of Complement component receptor 1-like protein [OS=Homo sapiens] | CR1L | 0,01 | -6,64 | 1E-17 |
| P31415 | Calsequestrin-1 [OS=Homo sapiens] | CASQ1 | 0,01 | -6,64 | 1E-17 |
| A9UHW6 | MIF4G domain-containing protein [OS=Homo sapiens] | MIF4GD | 0,01 | -6,64 | 1E-17 |
| Q9NP73-4 | Isoform 4 of Putative bifunctional UDP-N-acetylglucosamine transferase and deubiquitinase ALG13 [OS=Homo sapiens] | ALG13 | 0,01 | -6,64 | 1E-17 |
| Q13439 | Golgin subfamily A member 4 [OS=Homo sapiens] | GOLGA4 | 0,01 | -6,64 | 1E-17 |
| P22749-2 | Isoform Short of Granulysin [OS=Homo sapiens] | GNLY | 0,01 | -6,64 | 1E-17 |
| Q9Y5X1 | Sorting nexin-9 [OS=Homo sapiens] | SNX9 | 0,01 | -6,64 | 1E-17 |
| Q14978 | Nucleolar and coiled-body phosphoprotein 1 [OS=Homo sapiens] | NOLC1 | 0,01 | -6,64 | 1E-17 |
| Q16769 | Glutaminyl-peptide cyclotransferase [OS=Homo sapiens] | QPCT | 0,01 | -6,64 | 1E-17 |
| Q5VZF2 | Muscleblind-like protein 2 [OS=Homo sapiens] | MBNL2 | 0,01 | -6,64 | 1E-17 |
| P22681 | E3 ubiquitin-protein ligase CBL [OS=Homo sapiens] | CBL | 0,01 | -6,64 | 1E-17 |
| Q9H9J2 | 39S ribosomal protein L44, mitochondrial [OS=Homo sapiens] | MRPL44 | 0,01 | -6,64 | 1E-17 |
| Q96MU7 | YTH domain-containing protein 1 [OS=Homo sapiens] | YTHDC1 | 0,01 | -6,64 | 1E-17 |
| Q9UIY3 | RWD domain-containing protein 2A [OS=Homo sapiens] | RWDD2A | 0,01 | -6,64 | 1E-17 |
| Q9H825-2 | Isoform 2 of mRNA N(3)-methylcytidine methyltransferase METTL8 [OS=Homo sapiens] | METTL8 | 0,01 | -6,64 | 1E-17 |
| P61371 | Insulin gene enhancer protein ISL-1 [OS=Homo sapiens] | ISL1 | 0,01 | -6,64 | 1E-17 |
| Q9H172-3 | Isoform 3 of ATP-binding cassette sub-family G member 4 [OS=Homo sapiens] | ABCG4 | 0,01 | -6,64 | 1E-17 |
| P29374 | AT-rich interactive domain-containing protein 4A [OS=Homo sapiens] | ARID4A | 0,01 | -6,64 | 1E-17 |
| Q5VV43 | Dyslexia-associated protein KIAA0319 [OS=Homo sapiens] | KIAA0319 | 0,01 | -6,64 | 1E-17 |
| Q02045 | Myosin light chain 5 [OS=Homo sapiens] | MYL5 | 0,01 | -6,64 | 1E-17 |
| Q03989 | AT-rich interactive domain-containing protein 5A [OS=Homo sapiens] | ARID5A | 0,01 | -6,64 | 1E-17 |
| O94827-6 | Isoform 6 of Pleckstrin homology domain-containing family G member 5 [OS=Homo sapiens] | PLEKHG5 | 0,01 | -6,64 | 1E-17 |
| Q8N7R7 | Cyclin-Y-like protein 1 [OS=Homo sapiens] | CCNYL1 | 0,01 | -6,64 | 1E-17 |
| Q13938-4 | Isoform 3 of Calcyphosin [OS=Homo sapiens] | CAPS | 0,01 | -6,64 | 1E-17 |
| Q6AWC2-2 | Isoform 2 of Protein WWC2 [OS=Homo sapiens] | WWC2 | 0,01 | -6,64 | 1E-17 |
| Q9UQ13 | Leucine-rich repeat protein SHOC-2 [OS=Homo sapiens] | SHOC2 | 0,01 | -6,64 | 1E-17 |
| Q8NCK7 | Monocarboxylate transporter 11 [OS=Homo sapiens] | SLC16A11 | 0,019 | -5,75 | 6,33984E-10 |
| Q9BSQ5-2 | Isoform 2 of Cerebral cavernous malformations 2 protein [OS=Homo sapiens] | CCM2 | 0,021 | -5,6 | 1,67239E-09 |
| O14490-4 | Isoform 4 of Disks large-associated protein 1 [OS=Homo sapiens] | DLGAP1 | 0,028 | -5,18 | 2,42777E-08 |
| Q8NFM7-2 | Isoform 2 of Interleukin-17 receptor D [OS=Homo sapiens] | IL17RD | 0,033 | -4,92 | 1,13944E-07 |
| P35813-3 | Isoform 3 of Protein phosphatase 1A [OS=Homo sapiens] | PPM1A | 0,04 | -4,64 | 5,47727E-07 |
| O96028-2 | Isoform 2 of Histone-lysine N-methyltransferase NSD2 [OS=Homo sapiens] | NSD2 | 0,042 | -4,56 | 6,65542E-06 |
| Q9UK13 | Zinc finger protein 221 [OS=Homo sapiens] | ZNF221 | 0,046 | -4,43 | 1,63864E-06 |
| Q96B86-4 | Isoform 3 of Repulsive guidance molecule A [OS=Homo sapiens] | RGMA | 0,055 | -4,18 | 8,74811E-06 |
| Q86Y07-3 | Isoform 3 of Serine/threonine-protein kinase VRK2 [OS=Homo sapiens] | VRK2 | 0,07 | -3,84 | 3,00184E-05 |
| P11230 | Acetylcholine receptor subunit beta [OS=Homo sapiens] | CHRNB1 | 0,086 | -3,54 | 0,000113841 |
| Q5SRD1 | Mitochondrial import inner membrane translocase subunit Tim23B OS=Homo sapiens OX=9606 GN=TIMM23B PE=1 SV=3 | TIMM23B | 0,088 | -3,51 | 0,000131892 |
| P01034 | Cystatin-C [OS=Homo sapiens] | CST3 | 0,092 | -3,44 | 0,000499769 |
| Q8WU43 | Uncharacterized protein C2orf15 [OS=Homo sapiens] | C2orf15 | 0,099 | -3,33 | 0,000277594 |
| Q9H9T3-2 | Isoform 2 of Elongator complex protein 3 [OS=Homo sapiens] | ELP3 | 0,1 | -3,32 | 0,0002902 |
| Q6UWS5 | Protein PET117 homolog, mitochondrial [OS=Homo sapiens] | PET117 | 0,111 | -3,18 | 0,000513874 |
| Q86TL0-2 | Isoform 2 of Cysteine protease ATG4D [OS=Homo sapiens] | ATG4D | 0,117 | -3,1 | 0,001718181 |
| P57729 | Ras-related protein Rab-38 [OS=Homo sapiens] | RAB38 | 0,117 | -3,09 | 0,001312959 |
| Q9BQ16-6 | Isoform 6 of Testican-3 [OS=Homo sapiens] | SPOCK3 | 0,119 | -3,07 | 0,001540628 |
| Q9BRS8 | La-related protein 6 [OS=Homo sapiens] | LARP6 | 0,12 | -3,06 | 0,000814307 |
| O00422-2 | Isoform 2 of Histone deacetylase complex subunit SAP18 [OS=Homo sapiens] | SAP18 | 0,124 | -3,02 | 0,000955165 |
| Q96DN2 | von Willebrand factor C and EGF domain-containing protein [OS=Homo sapiens] | VWCE | 0,126 | -2,98 | 0,001077074 |
| Q03013 | Glutathione S-transferase Mu 4 [OS=Homo sapiens] | GSTM4 | 0,127 | -2,98 | 0,001080363 |
| Q96HW7 | Integrator complex subunit 4 [OS=Homo sapiens] | INTS4 | 0,134 | -2,9 | 0,003050855 |
| Q9UK00 | Uncharacterized protein C3orf18 [OS=Homo sapiens] | C3orf18 | 0,135 | -2,88 | 0,002403492 |
| Q9UJ70-2 | Isoform 2 of N-acetyl-D-glucosamine kinase [OS=Homo sapiens] | NAGK | 0,141 | -2,82 | 0,001928818 |
| P27037 | Activin receptor type-2A [OS=Homo sapiens] | ACVR2A | 0,145 | -2,78 | 0,002221533 |
| A6NML5 | Transmembrane protein 212 [OS=Homo sapiens] | TMEM212 | 0,148 | -2,76 | 0,002425536 |
| Q14CB8-4 | Isoform 4 of Rho GTPase-activating protein 19 [OS=Homo sapiens] | ARHGAP19 | 0,15 | -2,74 | 0,002573337 |
| Q569H4 | Protein Largen [OS=Homo sapiens] | PRR16 | 0,152 | -2,72 | 0,002741761 |
| Q9NZM6 | Polycystic kidney disease 2-like 2 protein [OS=Homo sapiens] | PKD2L2 | 0,154 | -2,7 | 0,003173194 |
| P31271 | Homeobox protein Hox-A13 [OS=Homo sapiens] | HOXA13 | 0,176 | -2,51 | 0,005578214 |
| Q6UWN5 | Ly6/PLAUR domain-containing protein 5 [OS=Homo sapiens] | LYPD5 | 0,182 | -2,46 | 0,006596569 |
| Q7Z3Z2 | Protein RD3 [OS=Homo sapiens] | RD3 | 0,189 | -2,41 | 0,007754362 |
| O14904 | Protein Wnt-9a [OS=Homo sapiens] | WNT9A | 0,19 | -2,39 | 0,013547695 |
| Q5JUX0 | Spindlin-3 [OS=Homo sapiens] | SPIN3 | 0,191 | -2,38 | 0,008289664 |
| Q9NZ63 | Telomere length and silencing protein 1 homolog [OS=Homo sapiens] | C9orf78 | 0,194 | -2,37 | 0,014578507 |
| O75953-5 | Isoform 3 of DnaJ homolog subfamily B member 5 [OS=Homo sapiens] | DNAJB5 | 0,196 | -2,35 | 0,009123699 |
| Q96MU8 | Kremen protein 1 [OS=Homo sapiens] | KREMEN1 | 0,197 | -2,35 | 0,009313974 |
| P54845 | Neural retina-specific leucine zipper protein [OS=Homo sapiens] | NRL | 0,198 | -2,34 | 0,010177507 |
| Q1RMZ1 | S-adenosylmethionine sensor upstream of mTORC1 [OS=Homo sapiens] | SAMTOR | 0,2 | -2,32 | 0,009960382 |
| Q96N16 | Janus kinase and microtubule-interacting protein 1 [OS=Homo sapiens] | JAKMIP1 | 0,201 | -2,32 | 0,010198465 |
| Q14117 | Dihydropyrimidinase [OS=Homo sapiens] | DPYS | 0,205 | -2,28 | 0,018309906 |
| Q8NCF5-3 | Isoform 3 of NFATC2-interacting protein [OS=Homo sapiens] | NFATC2IP | 0,205 | -2,29 | 0,011188169 |
| Q9Y4J8 | Dystrobrevin alpha [OS=Homo sapiens] | DTNA | 0,206 | -2,28 | 0,011436704 |
| Q69YI7 | Nuclear apoptosis-inducing factor 1 [OS=Homo sapiens] | NAIF1 | 0,207 | -2,27 | 0,011769062 |
| O95831-6 | Isoform 6 of Apoptosis-inducing factor 1, mitochondrial [OS=Homo sapiens] | AIFM1 | 0,209 | -2,26 | 0,012248566 |
| Q3T906 | N-acetylglucosamine-1-phosphotransferase subunits alpha/beta [OS=Homo sapiens] | GNPTAB | 0,21 | -2,25 | 0,012534307 |
| Q8WXJ9 | Ankyrin repeat and SOCS box protein 17 [OS=Homo sapiens] | ASB17 | 0,218 | -2,2 | 0,014458188 |
| Q9H9P5-3 | Isoform 3 of Putative E3 ubiquitin-protein ligase UNKL [OS=Homo sapiens] | UNKL | 0,225 | -2,15 | 0,016601605 |
| Q16778 | Histone H2B type 2-E [OS=Homo sapiens] | H2BC21 | 0,227 | -2,14 | 0,027239385 |
| P15692-14 | Isoform L-VEGF206 of Vascular endothelial growth factor A [OS=Homo sapiens] | VEGFA | 0,227 | -2,14 | 0,017326563 |
| A0A1B0GVQ0 | Small regulatory polypeptide of amino acid response [OS=Homo sapiens] | SPAAR | 0,235 | -2,09 | 0,019904814 |
| Q6UXD7 | Solute carrier family 49 member A3 [OS=Homo sapiens] | SLC49A3 | 0,239 | -2,07 | 0,021084657 |
| Q6P1J6-2 | Isoform 2 of Phospholipase B1, membrane-associated [OS=Homo sapiens] | PLB1 | 0,239 | -2,07 | 0,021084657 |
| P36575 | Arrestin-C [OS=Homo sapiens] | ARR3 | 0,254 | -1,98 | 0,026873737 |
| Q4G0Z9-4 | Isoform 4 of Minichromosome maintenance domain-containing protein 2 [OS=Homo sapiens] | MCMDC2 | 0,259 | -1,95 | 0,029202702 |
| P10415 | Apoptosis regulator Bcl-2 [OS=Homo sapiens] | BCL2 | 0,266 | -1,91 | 0,04093134 |
| Q5JTV8 | Torsin-1A-interacting protein 1 [OS=Homo sapiens] | TOR1AIP1 | 0,279 | -1,84 | 0,038516421 |
| Q15717-2 | Isoform 2 of ELAV-like protein 1 [OS=Homo sapiens] | ELAVL1 | 0,279 | -1,84 | 0,038516421 |
| O43295 | SLIT-ROBO Rho GTPase-activating protein 3 [OS=Homo sapiens] | SRGAP3 | 0,283 | -1,82 | 0,040604865 |
| Q8N9Z0 | Zinc finger protein 610 [OS=Homo sapiens] | ZNF610 | 0,287 | -1,8 | 0,04270729 |
| Q9NPJ4 | Proline-rich nuclear receptor coactivator 2 [OS=Homo sapiens] | PNRC2 | 0,287 | -1,8 | 0,042882155 |
| Q6PJT7-4 | Isoform 4 of Zinc finger CCCH domain-containing protein 14 [OS=Homo sapiens] | ZC3H14 | 4,089 | 2,03 | 0,044691702 |
| Q9H0B3 | IQ domain-containing protein N [OS=Homo sapiens] | IQCN | 4,374 | 2,13 | 0,034893267 |
| Q16585 | Beta-sarcoglycan [OS=Homo sapiens] | SGCB | 4,386 | 2,13 | 0,034526264 |
| P46100 | Transcriptional regulator ATRX [OS=Homo sapiens] | ATRX | 4,449 | 2,15 | 0,032730942 |
| Q7Z5A7 | Chemokine-like protein TAFA-5 [OS=Homo sapiens] | TAFA5 | 4,487 | 2,17 | 0,031692668 |
| Q5U651 | Ras-interacting protein 1 [OS=Homo sapiens] | RASIP1 | 4,525 | 2,18 | 0,03066839 |
| Q32NC0-2 | Isoform 2 of UPF0711 protein C18orf21 [OS=Homo sapiens] | C18orf21 | 4,64 | 2,21 | 0,027858973 |
| Q8IWB1 | Inositol 1,4,5-trisphosphate receptor-interacting protein [OS=Homo sapiens] | ITPRIP | 4,76 | 2,25 | 0,02521782 |
| Q9H6Z9 | Prolyl hydroxylase EGLN3 [OS=Homo sapiens] | EGLN3 | 4,863 | 2,28 | 0,038571571 |
| Q9UBL6 | Copine-7 [OS=Homo sapiens] | CPNE7 | 4,888 | 2,29 | 0,022698152 |
| Q5U5R9 | Probable E3 ubiquitin-protein ligase HECTD2 [OS=Homo sapiens] | HECTD2 | 4,992 | 2,32 | 0,020877816 |
| Q9H063 | Repressor of RNA polymerase III transcription MAF1 homolog [OS=Homo sapiens] | MAF1 | 5,223 | 2,38 | 0,03096111 |
| Q9P2N2-3 | Isoform 3 of Rho GTPase-activating protein 28 [OS=Homo sapiens] | ARHGAP28 | 5,397 | 2,43 | 0,015151979 |
| P51161 | Gastrotropin [OS=Homo sapiens] | FABP6 | 5,457 | 2,45 | 0,014460659 |
| Q16613-2 | Isoform 2 of Serotonin N-acetyltransferase [OS=Homo sapiens] | AANAT | 5,535 | 2,47 | 0,013623404 |
| P07205 | Phosphoglycerate kinase 2 [OS=Homo sapiens] | PGK2 | 5,932 | 2,57 | 0,010107015 |
| O75185 | Calcium-transporting ATPase type 2C member 2 [OS=Homo sapiens] | ATP2C2 | 6,017 | 2,59 | 0,00948726 |
| Q8N729 | Neuropeptide W [OS=Homo sapiens] | NPW | 6,172 | 2,63 | 0,017565021 |
| P19429 | Troponin I, cardiac muscle [OS=Homo sapiens] | TNNI3 | 6,622 | 2,73 | 0,006155705 |
| P0CB38 | Polyadenylate-binding protein 4-like [OS=Homo sapiens] | PABPC4L | 6,913 | 2,79 | 0,010293196 |
| O75157 | TSC22 domain family protein 2 [OS=Homo sapiens] | TSC22D2 | 6,953 | 2,8 | 0,004902426 |
| Q9Y2Q3 | Glutathione S-transferase kappa 1 [OS=Homo sapiens] | GSTK1 | 6,979 | 2,8 | 0,009065685 |
| Q6ZMM2 | ADAMTS-like protein 5 [OS=Homo sapiens] | ADAMTSL5 | 7,401 | 2,89 | 0,007088731 |
| Q5VWJ9 | Sorting nexin-30 [OS=Homo sapiens] | SNX30 | 8,133 | 3,02 | 0,006080606 |
| Q96PQ0 | VPS10 domain-containing receptor SorCS2 [OS=Homo sapiens] | SORCS2 | 8,464 | 3,08 | 0,001858366 |
| Q8N3J6-5 | Isoform 6 of Cell adhesion molecule 2 [OS=Homo sapiens] | CADM2 | 8,525 | 3,09 | 0,003964494 |
| A0A2R8Y7Y5 | Uncharacterized protein OS=Homo sapiens OX=9606 GN=LOC729966 PE=4 SV=1 | CIST1 | 9,298 | 3,22 | 0,003179174 |
| Q5XG92-6 | Isoform 5 of Carboxylesterase 4A [OS=Homo sapiens] | CES4A | 11,318 | 3,5 | 0,000380725 |
| Q8NFF5 | FAD synthase [OS=Homo sapiens] | FLAD1 | 11,572 | 3,53 | 0,000334785 |
| Q9NWW7 | Uncharacterized protein C2orf42 [OS=Homo sapiens] | C2orf42 | 12,081 | 3,59 | 0,000260068 |
| Q8N878 | FERM domain-containing protein 1 [OS=Homo sapiens] | FRMD1 | 13,03 | 3,7 | 0,000469482 |
| Q8NDI1 | EH domain-binding protein 1 [OS=Homo sapiens] | EHBP1 | 14,724 | 3,88 | 7,73519E-05 |
| O14782 | Kinesin-like protein KIF3C [OS=Homo sapiens] | KIF3C | 20,242 | 4,34 | 4,48364E-05 |
| O43300 | Leucine-rich repeat transmembrane neuronal protein 2 [OS=Homo sapiens] | LRRTM2 | 22,165 | 4,47 | 2,56539E-05 |
| A8MTZ0 | BBSome-interacting protein 1 [OS=Homo sapiens] | BBIP1 | 38,926 | 5,28 | 5,68639E-08 |
| Q9Y4R7-6 | Isoform 4 of Tubulin monoglycylase TTLL3 [OS=Homo sapiens] | TTLL3 | 100 | 6,64 | 1E-17 |
| P48544 | G protein-activated inward rectifier potassium channel 4 [OS=Homo sapiens] | KCNJ5 | 100 | 6,64 | 1E-17 |
| Q8N859 | Zinc finger protein 713 [OS=Homo sapiens] | ZNF713 | 100 | 6,64 | 1E-17 |
| Q5SYB0-2 | Isoform 2 of FERM and PDZ domain-containing protein 1 [OS=Homo sapiens] | FRMPD1 | 100 | 6,64 | 1E-17 |
| Q9NX31 | Oxidative stress-responsive serine-rich protein 1 [OS=Homo sapiens] | OSER1 | 100 | 6,64 | 1E-17 |
| Q8NEX6 | Protein WFDC11 [OS=Homo sapiens] | WFDC11 | 100 | 6,64 | 1E-17 |
| Q6ZYL4 | General transcription factor IIH subunit 5 [OS=Homo sapiens] | GTF2H5 | 100 | 6,64 | 1E-17 |
| O95922 | Probable tubulin polyglutamylase TTLL1 [OS=Homo sapiens] | TTLL1 | 100 | 6,64 | 1E-17 |
| Q9BQ51 | Programmed cell death 1 ligand 2 [OS=Homo sapiens] | PDCD1LG2 | 100 | 6,64 | 1E-17 |
| O75052 | Carboxyl-terminal PDZ ligand of neuronal nitric oxide synthase protein [OS=Homo sapiens] | NOS1AP | 100 | 6,64 | 1E-17 |
| Q16555 | Dihydropyrimidinase-related protein 2 [OS=Homo sapiens] | DPYSL2 | 100 | 6,64 | 1E-17 |
| P43246 | DNA mismatch repair protein Msh2 [OS=Homo sapiens] | MSH2 | 100 | 6,64 | 1E-17 |
| Q7L2E3-2 | Isoform 2 of ATP-dependent RNA helicase DHX30 [OS=Homo sapiens] | DHX30 | 100 | 6,64 | 1E-17 |
| Q96DT6 | Cysteine protease ATG4C [OS=Homo sapiens] | ATG4C | 100 | 6,64 | 1E-17 |
| Q8N335 | Glycerol-3-phosphate dehydrogenase 1-like protein [OS=Homo sapiens] | GPD1L | 100 | 6,64 | 1E-17 |
| O00584 | Ribonuclease T2 [OS=Homo sapiens] | RNASET2 | 100 | 6,64 | 1E-17 |
| O43566 | Regulator of G-protein signaling 14 [OS=Homo sapiens] | RGS14 | 100 | 6,64 | 1E-17 |
| Q8TDN6 | Ribosome biogenesis protein BRX1 homolog [OS=Homo sapiens] | BRIX1 | 100 | 6,64 | 1E-17 |
| A9YTQ3-3 | Isoform 3 of Aryl hydrocarbon receptor repressor [OS=Homo sapiens] | AHRR | 100 | 6,64 | 1E-17 |
| Q9NWY4 | Histone PARylation factor 1 [OS=Homo sapiens] | HPF1 | 100 | 6,64 | 1E-17 |
| P09104 | Gamma-enolase [OS=Homo sapiens] | ENO2 | 100 | 6,64 | 1E-17 |
| P51172-2 | Isoform 2 of Amiloride-sensitive sodium channel subunit delta [OS=Homo sapiens] | SCNN1D | 100 | 6,64 | 1E-17 |
| Q8TE54 | Anion exchange transporter [OS=Homo sapiens] | SLC26A7 | 100 | 6,64 | 1E-17 |
| P07954-2 | Isoform Cytoplasmic of Fumarate hydratase, mitochondrial [OS=Homo sapiens] | FH | 100 | 6,64 | 1E-17 |
| Q8N7X4 | Melanoma-associated antigen B6 [OS=Homo sapiens] | MAGEB6 | 100 | 6,64 | 1E-17 |
| P0DMW5 | Small integral membrane protein 10-like protein 2B [OS=Homo sapiens] | SMIM10L2B | 100 | 6,64 | 1E-17 |
| Q9H0Q0 | CYFIP-related Rac1 interactor A [OS=Homo sapiens] | CYRIA | 100 | 6,64 | 1E-17 |
| Q8NGI7 | Olfactory receptor 10V1 [OS=Homo sapiens] | OR10V1 | 100 | 6,64 | 1E-17 |
| Q8IWT0 | Protein archease [OS=Homo sapiens] | ZBTB8OS | 100 | 6,64 | 1E-17 |
| P17858 | ATP-dependent 6-phosphofructokinase, liver type [OS=Homo sapiens] | PFKL | 100 | 6,64 | 1E-17 |
| Q8NE28-3 | Isoform 3 of Serine/threonine kinase-like domain-containing protein STKLD1 [OS=Homo sapiens] | STKLD1 | 100 | 6,64 | 1E-17 |
| Q9Y219 | Protein jagged-2 [OS=Homo sapiens] | JAG2 | 100 | 6,64 | 1E-17 |
| O14526-3 | Isoform 3 of F-BAR domain only protein 1 [OS=Homo sapiens] | FCHO1 | 100 | 6,64 | 1E-17 |
| Q7Z5Y7-3 | Isoform 3 of BTB/POZ domain-containing protein KCTD20 [OS=Homo sapiens] | KCTD20 | 100 | 6,64 | 1E-17 |
| P06850 | Corticoliberin [OS=Homo sapiens] | CRH | 100 | 6,64 | 1E-17 |
| Q9BTD8 | RNA-binding protein 42 [OS=Homo sapiens] | RBM42 | 100 | 6,64 | 1E-17 |
| Q9UHV5 | Rap guanine nucleotide exchange factor-like 1 [OS=Homo sapiens] | RAPGEFL1 | 100 | 6,64 | 1E-17 |
| Q9H0F7 | ADP-ribosylation factor-like protein 6 [OS=Homo sapiens] | ARL6 | 100 | 6,64 | 1E-17 |
| B3EWG6 | Protein FAM25G [OS=Homo sapiens] | FAM25G | 100 | 6,64 | 1E-17 |
| Q9NV64 | Transmembrane protein 39A [OS=Homo sapiens] | TMEM39A | 100 | 6,64 | 1E-17 |
| Q15596 | Nuclear receptor coactivator 2 [OS=Homo sapiens] | NCOA2 | 100 | 6,64 | 1E-17 |
| Q8IUI8 | Cytokine receptor-like factor 3 [OS=Homo sapiens] | CRLF3 | 100 | 6,64 | 1E-17 |
| Q9Y237 | Peptidyl-prolyl cis-trans isomerase NIMA-interacting 4 [OS=Homo sapiens] | PIN4 | 100 | 6,64 | 1E-17 |
| Q8WVY7 | Ubiquitin-like domain-containing CTD phosphatase 1 [OS=Homo sapiens] | UBLCP1 | 100 | 6,64 | 1E-17 |
| O15212 | Prefoldin subunit 6 [OS=Homo sapiens] | PFDN6 | 100 | 6,64 | 1E-17 |
| Q2TAZ0-4 | Isoform 3 of Autophagy-related protein 2 homolog A [OS=Homo sapiens] | ATG2A | 100 | 6,64 | 1E-17 |
| P60604 | Ubiquitin-conjugating enzyme E2 G2 [OS=Homo sapiens] | UBE2G2 | 100 | 6,64 | 1E-17 |
| A6NJZ3 | Olfactory receptor 6C65 [OS=Homo sapiens] | OR6C65 | 100 | 6,64 | 1E-17 |

### D) Dabigatran

| Accession | Description | Gene Symbol | Abundance Ratio | Abundance Ratio (log2) | Abundance Ratio  P-Value |
| --- | --- | --- | --- | --- | --- |
| Q03013 | Glutathione S-transferase Mu 4 [OS=Homo sapiens] | GSTM4 | 0,01 | -6,64 | 1E-17 |
| P54845 | Neural retina-specific leucine zipper protein [OS=Homo sapiens] | NRL | 0,01 | -6,64 | 1E-17 |
| Q5T7W7 | Thiosulfate sulfurtransferase/rhodanese-like domain-containing protein 2 [OS=Homo sapiens] | TSTD2 | 0,01 | -6,64 | 1E-17 |
| O75952-6 | Isoform 6 of Calcium-binding tyrosine phosphorylation-regulated protein [OS=Homo sapiens] | CABYR | 0,01 | -6,64 | 1E-17 |
| Q96PE3 | Inositol polyphosphate-4-phosphatase type I A [OS=Homo sapiens] | INPP4A | 0,01 | -6,64 | 1E-17 |
| Q12765 | Secernin-1 [OS=Homo sapiens] | SCRN1 | 0,01 | -6,64 | 1E-17 |
| P29372 | DNA-3-methyladenine glycosylase [OS=Homo sapiens] | MPG | 0,01 | -6,64 | 1E-17 |
| Q5JUX0 | Spindlin-3 [OS=Homo sapiens] | SPIN3 | 0,01 | -6,64 | 1E-17 |
| Q4G0J3 | La-related protein 7 [OS=Homo sapiens] | LARP7 | 0,01 | -6,64 | 1E-17 |
| O96028-2 | Isoform 2 of Histone-lysine N-methyltransferase NSD2 [OS=Homo sapiens] | NSD2 | 0,01 | -6,64 | 1E-17 |
| A9UHW6 | MIF4G domain-containing protein [OS=Homo sapiens] | MIF4GD | 0,01 | -6,64 | 1E-17 |
| Q8WU43 | Uncharacterized protein C2orf15 [OS=Homo sapiens] | C2orf15 | 0,01 | -6,64 | 1E-17 |
| Q8N729 | Neuropeptide W [OS=Homo sapiens] | NPW | 0,01 | -6,64 | 1E-17 |
| P22749-2 | Isoform Short of Granulysin [OS=Homo sapiens] | GNLY | 0,01 | -6,64 | 1E-17 |
| Q9Y5X1 | Sorting nexin-9 [OS=Homo sapiens] | SNX9 | 0,01 | -6,64 | 1E-17 |
| Q3KNS6-3 | Isoform 3 of Zinc finger protein 829 [OS=Homo sapiens] | ZNF829 | 0,01 | -6,64 | 1E-17 |
| Q9NU63 | Zinc finger protein 57 homolog [OS=Homo sapiens] | ZFP57 | 0,01 | -6,64 | 1E-17 |
| Q9UBL6 | Copine-7 [OS=Homo sapiens] | CPNE7 | 0,01 | -6,64 | 1E-17 |
| Q8N2W9 | E3 SUMO-protein ligase PIAS4 [OS=Homo sapiens] | PIAS4 | 0,01 | -6,64 | 1E-17 |
| Q16831 | Uridine phosphorylase 1 [OS=Homo sapiens] | UPP1 | 0,01 | -6,64 | 1E-17 |
| Q9H9J2 | 39S ribosomal protein L44, mitochondrial [OS=Homo sapiens] | MRPL44 | 0,01 | -6,64 | 1E-17 |
| Q9UIY3 | RWD domain-containing protein 2A [OS=Homo sapiens] | RWDD2A | 0,01 | -6,64 | 1E-17 |
| Q9NXT0-2 | Isoform 2 of Zinc finger protein 586 [OS=Homo sapiens] | ZNF586 | 0,01 | -6,64 | 1E-17 |
| Q4G0Z9-4 | Isoform 4 of Minichromosome maintenance domain-containing protein 2 [OS=Homo sapiens] | MCMDC2 | 0,01 | -6,64 | 1E-17 |
| Q5EBL8-2 | Isoform 2 of PDZ domain-containing protein 11 [OS=Homo sapiens] | PDZD11 | 0,01 | -6,64 | 1E-17 |
| Q9UPV7 | PHD finger protein 24 [OS=Homo sapiens] | PHF24 | 0,01 | -6,64 | 1E-17 |
| P29374 | AT-rich interactive domain-containing protein 4A [OS=Homo sapiens] | ARID4A | 0,01 | -6,64 | 1E-17 |
| Q9NZM6 | Polycystic kidney disease 2-like 2 protein [OS=Homo sapiens] | PKD2L2 | 0,01 | -6,64 | 1E-17 |
| P56545 | C-terminal-binding protein 2 [OS=Homo sapiens] | CTBP2 | 0,01 | -6,64 | 1E-17 |
| Q7Z7A1-2 | Isoform 2 of Centriolin [OS=Homo sapiens] | CNTRL | 0,01 | -6,64 | 1E-17 |
| Q02045 | Myosin light chain 5 [OS=Homo sapiens] | MYL5 | 0,01 | -6,64 | 1E-17 |
| P57729 | Ras-related protein Rab-38 [OS=Homo sapiens] | RAB38 | 0,01 | -6,64 | 1E-17 |
| Q9UK00 | Uncharacterized protein C3orf18 [OS=Homo sapiens] | C3orf18 | 0,01 | -6,64 | 1E-17 |
| Q03989 | AT-rich interactive domain-containing protein 5A [OS=Homo sapiens] | ARID5A | 0,01 | -6,64 | 1E-17 |
| Q7L1S5 | Carbohydrate sulfotransferase 9 [OS=Homo sapiens] | CHST9 | 0,01 | -6,64 | 1E-17 |
| Q8N7R7 | Cyclin-Y-like protein 1 [OS=Homo sapiens] | CCNYL1 | 0,01 | -6,64 | 1E-17 |
| Q3ZCX4-2 | Isoform 2 of Zinc finger protein 568 [OS=Homo sapiens] | ZNF568 | 0,01 | -6,64 | 1E-17 |
| Q6UWN5 | Ly6/PLAUR domain-containing protein 5 [OS=Homo sapiens] | LYPD5 | 0,01 | -6,64 | 1E-17 |
| Q6AWC2-2 | Isoform 2 of Protein WWC2 [OS=Homo sapiens] | WWC2 | 0,01 | -6,64 | 1E-17 |
| O75177 | Calcium-responsive transactivator [OS=Homo sapiens] | SS18L1 | 0,011 | -6,55 | 6,28346E-11 |
| Q8IYP9 | Palmitoyltransferase ZDHHC23 [OS=Homo sapiens] | ZDHHC23 | 0,011 | -6,44 | 1,26466E-10 |
| Q8NCK7 | Monocarboxylate transporter 11 [OS=Homo sapiens] | SLC16A11 | 0,014 | -6,13 | 9,02409E-10 |
| P27037 | Activin receptor type-2A [OS=Homo sapiens] | ACVR2A | 0,014 | -6,12 | 1,00487E-09 |
| Q15256-5 | Isoform 4 of Receptor-type tyrosine-protein phosphatase R [OS=Homo sapiens] | PTPRR | 0,022 | -5,49 | 3,93328E-08 |
| Q9BSQ5-2 | Isoform 2 of Cerebral cavernous malformations 2 protein [OS=Homo sapiens] | CCM2 | 0,032 | -4,99 | 5,99485E-07 |
| P35813-3 | Isoform 3 of Protein phosphatase 1A [OS=Homo sapiens] | PPM1A | 0,034 | -4,88 | 1,00744E-06 |
| O00422-2 | Isoform 2 of Histone deacetylase complex subunit SAP18 [OS=Homo sapiens] | SAP18 | 0,035 | -4,84 | 1,24045E-06 |
| P61018-2 | Isoform 2 of Ras-related protein Rab-4B [OS=Homo sapiens] | RAB4B | 0,043 | -4,53 | 5,63315E-06 |
| P22681 | E3 ubiquitin-protein ligase CBL [OS=Homo sapiens] | CBL | 0,056 | -4,17 | 2,86469E-05 |
| Q96MU8 | Kremen protein 1 [OS=Homo sapiens] | KREMEN1 | 0,059 | -4,08 | 5,91791E-05 |
| O00305-3 | Isoform 3 of Voltage-dependent L-type calcium channel subunit beta-4 [OS=Homo sapiens] | CACNB4 | 0,062 | -4,01 | 5,77552E-05 |
| Q9C0C2-2 | Isoform 2 of 182 kDa tankyrase-1-binding protein [OS=Homo sapiens] | TNKS1BP1 | 0,065 | -3,94 | 7,53975E-05 |
| Q86Y07-3 | Isoform 3 of Serine/threonine-protein kinase VRK2 [OS=Homo sapiens] | VRK2 | 0,075 | -3,74 | 0,000171261 |
| Q14CB8-4 | Isoform 4 of Rho GTPase-activating protein 19 [OS=Homo sapiens] | ARHGAP19 | 0,077 | -3,71 | 0,00019338 |
| Q9H9T3-2 | Isoform 2 of Elongator complex protein 3 [OS=Homo sapiens] | ELP3 | 0,08 | -3,64 | 0,000253809 |
| Q9BRS8 | La-related protein 6 [OS=Homo sapiens] | LARP6 | 0,085 | -3,56 | 0,000335208 |
| Q5SRD1 | Mitochondrial import inner membrane translocase subunit Tim23B OS=Homo sapiens OX=9606 GN=TIMM23B PE=1 SV=3 | TIMM23B | 0,088 | -3,5 | 0,00042312 |
| O95831-6 | Isoform 6 of Apoptosis-inducing factor 1, mitochondrial [OS=Homo sapiens] | AIFM1 | 0,091 | -3,46 | 0,000495936 |
| Q9Y4J8 | Dystrobrevin alpha [OS=Homo sapiens] | DTNA | 0,092 | -3,44 | 0,000537457 |
| Q9BQ16-6 | Isoform 6 of Testican-3 [OS=Homo sapiens] | SPOCK3 | 0,094 | -3,41 | 0,00091634 |
| A0A1B0GVQ0 | Small regulatory polypeptide of amino acid response [OS=Homo sapiens] | SPAAR | 0,096 | -3,38 | 0,000671063 |
| O00142 | Thymidine kinase 2, mitochondrial [OS=Homo sapiens] | TK2 | 0,102 | -3,29 | 0,000924036 |
| Q9NR64 | Kelch-like protein 1 [OS=Homo sapiens] | KLHL1 | 0,108 | -3,21 | 0,001228092 |
| Q9H6X5 | Uncharacterized protein C19orf44 [OS=Homo sapiens] | C19orf44 | 0,108 | -3,21 | 0,002280393 |
| Q569H4 | Protein Largen [OS=Homo sapiens] | PRR16 | 0,108 | -3,21 | 0,00120573 |
| P09564 | T-cell antigen CD7 [OS=Homo sapiens] | CD7 | 0,11 | -3,19 | 0,002215643 |
| Q86TL0-2 | Isoform 2 of Cysteine protease ATG4D [OS=Homo sapiens] | ATG4D | 0,113 | -3,15 | 0,002429177 |
| O43295 | SLIT-ROBO Rho GTPase-activating protein 3 [OS=Homo sapiens] | SRGAP3 | 0,118 | -3,09 | 0,001853077 |
| P01034 | Cystatin-C [OS=Homo sapiens] | CST3 | 0,125 | -3 | 0,006546916 |
| Q8WUS8 | Short-chain dehydrogenase/reductase family 42E member 1 [OS=Homo sapiens] | SDR42E1 | 0,126 | -2,99 | 0,008138374 |
| Q06889-2 | Isoform 2 of Early growth response protein 3 [OS=Homo sapiens] | EGR3 | 0,131 | -2,93 | 0,005275403 |
| Q5HYR2 | Doublesex- and mab-3-related transcription factor C1 [OS=Homo sapiens] | DMRTC1; DMRTC1B | 0,133 | -2,91 | 0,00331683 |
| Q9H503 | Barrier-to-autointegration factor-like protein [OS=Homo sapiens] | BANF2 | 0,134 | -2,9 | 0,003371646 |
| Q6ZU67-3 | Isoform 3 of BEN domain-containing protein 4 [OS=Homo sapiens] | BEND4 | 0,138 | -2,86 | 0,003835827 |
| Q8WXJ9 | Ankyrin repeat and SOCS box protein 17 [OS=Homo sapiens] | ASB17 | 0,15 | -2,74 | 0,005586489 |
| Q5T124-8 | Isoform 8 of UBX domain-containing protein 11 [OS=Homo sapiens] | UBXN11 | 0,152 | -2,72 | 0,006022656 |
| P11230 | Acetylcholine receptor subunit beta [OS=Homo sapiens] | CHRNB1 | 0,159 | -2,65 | 0,00726046 |
| P25963 | NF-kappa-B inhibitor alpha [OS=Homo sapiens] | NFKBIA | 0,165 | -2,6 | 0,008547769 |
| Q9UJ70-2 | Isoform 2 of N-acetyl-D-glucosamine kinase [OS=Homo sapiens] | NAGK | 0,175 | -2,51 | 0,010876124 |
| A8MWX3 | Putative WAS protein family homolog 4 [OS=Homo sapiens] | WASH4P | 0,176 | -2,51 | 0,011417989 |
| Q16769 | Glutaminyl-peptide cyclotransferase [OS=Homo sapiens] | QPCT | 0,177 | -2,5 | 0,011417836 |
| Q96DN2 | von Willebrand factor C and EGF domain-containing protein [OS=Homo sapiens] | VWCE | 0,177 | -2,5 | 0,011380897 |
| Q96N16 | Janus kinase and microtubule-interacting protein 1 [OS=Homo sapiens] | JAKMIP1 | 0,178 | -2,49 | 0,011534014 |
| Q69YI7 | Nuclear apoptosis-inducing factor 1 [OS=Homo sapiens] | NAIF1 | 0,178 | -2,49 | 0,011703891 |
| Q8NB12 | Histone-lysine N-methyltransferase SMYD1 [OS=Homo sapiens] | SMYD1 | 0,18 | -2,47 | 0,012192104 |
| Q96PM5-6 | Isoform 6 of RING finger and CHY zinc finger domain-containing protein 1 [OS=Homo sapiens] | RCHY1 | 0,183 | -2,45 | 0,01288293 |
| Q658Y4 | Protein FAM91A1 [OS=Homo sapiens] | FAM91A1 | 0,185 | -2,44 | 0,013495656 |
| P01344 | Insulin-like growth factor II [OS=Homo sapiens] | IGF2 | 0,19 | -2,39 | 0,015089354 |
| Q06730-3 | Isoform 3 of Zinc finger protein 33A [OS=Homo sapiens] | ZNF33A | 0,192 | -2,38 | 0,015691462 |
| Q3T906 | N-acetylglucosamine-1-phosphotransferase subunits alpha/beta [OS=Homo sapiens] | GNPTAB | 0,193 | -2,38 | 0,015883326 |
| Q9NZ63 | Telomere length and silencing protein 1 homolog [OS=Homo sapiens] | C9orf78 | 0,196 | -2,35 | 0,025773134 |
| Q9Y3T9 | Nucleolar complex protein 2 homolog [OS=Homo sapiens] | NOC2L | 0,201 | -2,32 | 0,02390909 |
| Q02413-2 | Isoform 2 of Desmoglein-1 [OS=Homo sapiens] | DSG1 | 0,202 | -2,31 | 0,018986718 |
| P61371 | Insulin gene enhancer protein ISL-1 [OS=Homo sapiens] | ISL1 | 0,206 | -2,28 | 0,020579774 |
| O15527 | N-glycosylase/DNA lyase [OS=Homo sapiens] | OGG1 | 0,208 | -2,27 | 0,028823505 |
| Q99598 | Translin-associated protein X [OS=Homo sapiens] | TSNAX | 0,21 | -2,25 | 0,021996663 |
| P22090 | 40S ribosomal protein S4, Y isoform 1 [OS=Homo sapiens] | RPS4Y1 | 0,211 | -2,24 | 0,031740718 |
| P82933 | 28S ribosomal protein S9, mitochondrial [OS=Homo sapiens] | MRPS9 | 0,213 | -2,23 | 0,023128679 |
| O75953-5 | Isoform 3 of DnaJ homolog subfamily B member 5 [OS=Homo sapiens] | DNAJB5 | 0,214 | -2,22 | 0,023747203 |
| Q9H310 | Ammonium transporter Rh type B OS=Homo sapiens OX=9606 GN=RHBG PE=1 SV=3 | RHBG | 0,22 | -2,18 | 0,026291036 |
| P26006 | Integrin alpha-3 [OS=Homo sapiens] | ITGA3 | 0,224 | -2,16 | 0,028015237 |
| O14490-4 | Isoform 4 of Disks large-associated protein 1 [OS=Homo sapiens] | DLGAP1 | 0,23 | -2,12 | 0,030734921 |
| Q9BZE4-3 | Isoform 3 of Nucleolar GTP-binding protein 1 [OS=Homo sapiens] | GTPBP4 | 0,231 | -2,11 | 0,03129647 |
| Q9NYK5 | 39S ribosomal protein L39, mitochondrial [OS=Homo sapiens] | MRPL39 | 0,238 | -2,07 | 0,035009781 |
| Q1RMZ1 | S-adenosylmethionine sensor upstream of mTORC1 [OS=Homo sapiens] | SAMTOR | 0,245 | -2,03 | 0,038512322 |
| Q96PG2 | Membrane-spanning 4-domains subfamily A member 10 [OS=Homo sapiens] | MS4A10 | 0,248 | -2,01 | 0,040396251 |
| P45877 | Peptidyl-prolyl cis-trans isomerase C [OS=Homo sapiens] | PPIC | 4,194 | 2,07 | 0,047608515 |
| Q53RT3 | Retroviral-like aspartic protease 1 [OS=Homo sapiens] | ASPRV1 | 4,258 | 2,09 | 0,047079109 |
| Q9NYP8 | Uncharacterized protein C21orf62 [OS=Homo sapiens] | C21orf62 | 4,38 | 2,13 | 0,041083714 |
| Q92771 | Putative ATP-dependent RNA helicase DDX12 [OS=Homo sapiens] | DDX12P | 4,535 | 2,18 | 0,03643147 |
| P46100 | Transcriptional regulator ATRX [OS=Homo sapiens] | ATRX | 4,691 | 2,23 | 0,032318759 |
| Q5U5R9 | Probable E3 ubiquitin-protein ligase HECTD2 [OS=Homo sapiens] | HECTD2 | 4,784 | 2,26 | 0,030130463 |
| P17025 | Zinc finger protein 182 [OS=Homo sapiens] | ZNF182 | 4,789 | 2,26 | 0,038900437 |
| O43155 | Leucine-rich repeat transmembrane protein FLRT2 [OS=Homo sapiens] | FLRT2 | 4,981 | 2,32 | 0,036028051 |
| Q16613-2 | Isoform 2 of Serotonin N-acetyltransferase [OS=Homo sapiens] | AANAT | 4,996 | 2,32 | 0,025732573 |
| Q01543-2 | Isoform 2 of Friend leukemia integration 1 transcription factor [OS=Homo sapiens] | FLI1 | 5,088 | 2,35 | 0,024058922 |
| Q96FC9-3 | Isoform 3 of ATP-dependent DNA helicase DDX11 [OS=Homo sapiens] | DDX11 | 5,258 | 2,39 | 0,027913771 |
| Q9H6U6 | Breast carcinoma-amplified sequence 3 [OS=Homo sapiens] | BCAS3 | 5,281 | 2,4 | 0,028770568 |
| Q7Z5A7 | Chemokine-like protein TAFA-5 [OS=Homo sapiens] | TAFA5 | 5,284 | 2,4 | 0,020889938 |
| Q8NA61-2 | Isoform 2 of Protein chibby homolog 2 [OS=Homo sapiens] | CBY2 | 5,308 | 2,41 | 0,027379988 |
| A6NJY4 | Putative transmembrane protein LOC100289255 [OS=Homo sapiens] | TMEM238L | 5,497 | 2,46 | 0,024779411 |
| Q969R2 | Oxysterol-binding protein 2 [OS=Homo sapiens] | OSBP2 | 5,569 | 2,48 | 0,017085241 |
| Q6ZMM2 | ADAMTS-like protein 5 [OS=Homo sapiens] | ADAMTSL5 | 5,796 | 2,54 | 0,0216371 |
| Q9HB75-6 | Isoform 6 of p53-induced death domain-containing protein 1 [OS=Homo sapiens] | PIDD1 | 5,986 | 2,58 | 0,012845675 |
| O43300 | Leucine-rich repeat transmembrane neuronal protein 2 [OS=Homo sapiens] | LRRTM2 | 6,153 | 2,62 | 0,016504727 |
| Q6PJT7-4 | Isoform 4 of Zinc finger CCCH domain-containing protein 14 [OS=Homo sapiens] | ZC3H14 | 6,163 | 2,62 | 0,011425816 |
| P07205 | Phosphoglycerate kinase 2 [OS=Homo sapiens] | PGK2 | 6,903 | 2,79 | 0,00711943 |
| Q96DZ9 | CKLF-like MARVEL transmembrane domain-containing protein 5 [OS=Homo sapiens] | CMTM5 | 7,306 | 2,87 | 0,005569981 |
| Q9Y2Q3 | Glutathione S-transferase kappa 1 [OS=Homo sapiens] | GSTK1 | 7,433 | 2,89 | 0,00733962 |
| Q8NDI1 | EH domain-binding protein 1 [OS=Homo sapiens] | EHBP1 | 7,435 | 2,89 | 0,005158057 |
| O14530 | Thioredoxin domain-containing protein 9 [OS=Homo sapiens] | TXNDC9 | 7,624 | 2,93 | 0,004614635 |
| O75185 | Calcium-transporting ATPase type 2C member 2 [OS=Homo sapiens] | ATP2C2 | 7,674 | 2,94 | 0,004482364 |
| Q96EZ8-3 | Isoform 3 of Microspherule protein 1 [OS=Homo sapiens] | MCRS1 | 8,248 | 3,04 | 0,003229742 |
| O75157 | TSC22 domain family protein 2 [OS=Homo sapiens] | TSC22D2 | 8,441 | 3,08 | 0,002902447 |
| P0CB38 | Polyadenylate-binding protein 4-like [OS=Homo sapiens] | PABPC4L | 8,984 | 3,17 | 0,003599977 |
| Q8N3J6-5 | Isoform 6 of Cell adhesion molecule 2 [OS=Homo sapiens] | CADM2 | 9,212 | 3,2 | 0,00403304 |
| Q8N878 | FERM domain-containing protein 1 [OS=Homo sapiens] | FRMD1 | 9,551 | 3,26 | 0,003295177 |
| Q8NFF5 | FAD synthase [OS=Homo sapiens] | FLAD1 | 9,908 | 3,31 | 0,001343723 |
| Q14978 | Nucleolar and coiled-body phosphoprotein 1 [OS=Homo sapiens] | NOLC1 | 10,27 | 3,36 | 0,001123717 |
| A0A2R8Y7Y5 | Uncharacterized protein OS=Homo sapiens OX=9606 GN=LOC729966 PE=4 SV=1 | CIST1 | 11,583 | 3,53 | 0,001144573 |
| Q9UFH2 | Dynein heavy chain 17, axonemal [OS=Homo sapiens] | DNAH17 | 11,779 | 3,56 | 0,000554375 |
| Q8N584-2 | Isoform 2 of Tetratricopeptide repeat protein 39C [OS=Homo sapiens] | TTC39C | 12,936 | 3,69 | 0,000334946 |
| Q5XG92-6 | Isoform 5 of Carboxylesterase 4A [OS=Homo sapiens] | CES4A | 14,326 | 3,84 | 0,000189846 |
| Q155Q3-2 | Isoform 2 of Dixin [OS=Homo sapiens] | DIXDC1 | 15,569 | 3,96 | 0,000236371 |
| Q9NWW7 | Uncharacterized protein C2orf42 [OS=Homo sapiens] | C2orf42 | 18,624 | 4,22 | 4,02186E-05 |
| Q13439 | Golgin subfamily A member 4 [OS=Homo sapiens] | GOLGA4 | 27,837 | 4,8 | 1,57833E-05 |
| A8MTZ0 | BBSome-interacting protein 1 [OS=Homo sapiens] | BBIP1 | 32,674 | 5,03 | 9,2141E-07 |
| A6NJZ3 | Olfactory receptor 6C65 [OS=Homo sapiens] | OR6C65 | 72,54 | 6,18 | 1,49554E-09 |
| P49796-2 | Isoform 2 of Regulator of G-protein signaling 3 [OS=Homo sapiens] | RGS3 | 100 | 6,64 | 1E-17 |
| Q9Y4R7-6 | Isoform 4 of Tubulin monoglycylase TTLL3 [OS=Homo sapiens] | TTLL3 | 100 | 6,64 | 1E-17 |
| Q9H0E9 | Bromodomain-containing protein 8 [OS=Homo sapiens] | BRD8 | 100 | 6,64 | 1E-17 |
| P48544 | G protein-activated inward rectifier potassium channel 4 [OS=Homo sapiens] | KCNJ5 | 100 | 6,64 | 1E-17 |
| Q8N859 | Zinc finger protein 713 [OS=Homo sapiens] | ZNF713 | 100 | 6,64 | 1E-17 |
| O75896 | Tumor suppressor candidate 2 [OS=Homo sapiens] | TUSC2 | 100 | 6,64 | 1E-17 |
| Q53GS9 | U4/U6.U5 tri-snRNP-associated protein 2 [OS=Homo sapiens] | USP39 | 100 | 6,64 | 1E-17 |
| Q96K58-2 | Isoform 2 of Zinc finger protein 668 [OS=Homo sapiens] | TP53 | 100 | 6,64 | 1E-17 |
| Q9NX31 | Oxidative stress-responsive serine-rich protein 1 [OS=Homo sapiens] | OSER1 | 100 | 6,64 | 1E-17 |
| Q8NEX6 | Protein WFDC11 [OS=Homo sapiens] | WFDC11 | 100 | 6,64 | 1E-17 |
| P60153-2 | Isoform 2 of Inactive ribonuclease-like protein 9 [OS=Homo sapiens] | RNASE9 | 100 | 6,64 | 1E-17 |
| O95922 | Probable tubulin polyglutamylase TTLL1 [OS=Homo sapiens] | TTLL1 | 100 | 6,64 | 1E-17 |
| Q9BQ51 | Programmed cell death 1 ligand 2 [OS=Homo sapiens] | PDCD1LG2 | 100 | 6,64 | 1E-17 |
| Q16555 | Dihydropyrimidinase-related protein 2 [OS=Homo sapiens] | DPYSL2 | 100 | 6,64 | 1E-17 |
| Q5VT52 | Regulation of nuclear pre-mRNA domain-containing protein 2 [OS=Homo sapiens] | RPRD2 | 100 | 6,64 | 1E-17 |
| P43246 | DNA mismatch repair protein Msh2 [OS=Homo sapiens] | MSH2 | 100 | 6,64 | 1E-17 |
| Q96DT6 | Cysteine protease ATG4C [OS=Homo sapiens] | ATG4C | 100 | 6,64 | 1E-17 |
| O75323 | Protein NipSnap homolog 2 [OS=Homo sapiens] | NIPSNAP2 | 100 | 6,64 | 1E-17 |
| O43566 | Regulator of G-protein signaling 14 [OS=Homo sapiens] | RGS14 | 100 | 6,64 | 1E-17 |
| Q8TDN6 | Ribosome biogenesis protein BRX1 homolog [OS=Homo sapiens] | BRIX1 | 100 | 6,64 | 1E-17 |
| A9YTQ3-3 | Isoform 3 of Aryl hydrocarbon receptor repressor [OS=Homo sapiens] | AHRR | 100 | 6,64 | 1E-17 |
| Q9NWY4 | Histone PARylation factor 1 [OS=Homo sapiens] | HPF1 | 100 | 6,64 | 1E-17 |
| P09104 | Gamma-enolase [OS=Homo sapiens] | ENO2 | 100 | 6,64 | 1E-17 |
| P51172-2 | Isoform 2 of Amiloride-sensitive sodium channel subunit delta [OS=Homo sapiens] | SCNN1D | 100 | 6,64 | 1E-17 |
| Q8TE54 | Anion exchange transporter [OS=Homo sapiens] | SLC26A7 | 100 | 6,64 | 1E-17 |
| P0DMW5 | Small integral membrane protein 10-like protein 2B [OS=Homo sapiens] | SMIM10L2B | 100 | 6,64 | 1E-17 |
| Q9H0Q0 | CYFIP-related Rac1 interactor A [OS=Homo sapiens] | CYRIA | 100 | 6,64 | 1E-17 |
| Q9BPX1 | 17-beta-hydroxysteroid dehydrogenase 14 [OS=Homo sapiens] | HSD17B14 | 100 | 6,64 | 1E-17 |
| Q8IWT0 | Protein archease [OS=Homo sapiens] | ZBTB8OS | 100 | 6,64 | 1E-17 |
| P17858 | ATP-dependent 6-phosphofructokinase, liver type [OS=Homo sapiens] | PFKL | 100 | 6,64 | 1E-17 |
| Q8NE28-3 | Isoform 3 of Serine/threonine kinase-like domain-containing protein STKLD1 [OS=Homo sapiens] | STKLD1 | 100 | 6,64 | 1E-17 |
| Q9Y219 | Protein jagged-2 [OS=Homo sapiens] | JAG2 | 100 | 6,64 | 1E-17 |
| O14526-3 | Isoform 3 of F-BAR domain only protein 1 [OS=Homo sapiens] | FCHO1 | 100 | 6,64 | 1E-17 |
| P00167 | Cytochrome b5 [OS=Homo sapiens] | CYB5A | 100 | 6,64 | 1E-17 |
| Q7Z5Y7-3 | Isoform 3 of BTB/POZ domain-containing protein KCTD20 [OS=Homo sapiens] | KCTD20 | 100 | 6,64 | 1E-17 |
| Q9BTD8 | RNA-binding protein 42 [OS=Homo sapiens] | RBM42 | 100 | 6,64 | 1E-17 |
| Q9UHV5 | Rap guanine nucleotide exchange factor-like 1 [OS=Homo sapiens] | RAPGEFL1 | 100 | 6,64 | 1E-17 |
| Q9HB58-4 | Isoform 4 of Sp110 nuclear body protein [OS=Homo sapiens] | SP110 | 100 | 6,64 | 1E-17 |
| Q9H0F7 | ADP-ribosylation factor-like protein 6 [OS=Homo sapiens] | ARL6 | 100 | 6,64 | 1E-17 |
| Q14524 | Sodium channel protein type 5 subunit alpha [OS=Homo sapiens] | SCN5A | 100 | 6,64 | 1E-17 |
| B3EWG6 | Protein FAM25G [OS=Homo sapiens] | FAM25G | 100 | 6,64 | 1E-17 |
| Q9NV64 | Transmembrane protein 39A [OS=Homo sapiens] | TMEM39A | 100 | 6,64 | 1E-17 |
| Q8IUI8 | Cytokine receptor-like factor 3 [OS=Homo sapiens] | CRLF3 | 100 | 6,64 | 1E-17 |
| Q9Y237 | Peptidyl-prolyl cis-trans isomerase NIMA-interacting 4 [OS=Homo sapiens] | PIN4 | 100 | 6,64 | 1E-17 |
| Q8WVY7 | Ubiquitin-like domain-containing CTD phosphatase 1 [OS=Homo sapiens] | UBLCP1 | 100 | 6,64 | 1E-17 |
| O15212 | Prefoldin subunit 6 [OS=Homo sapiens] | PFDN6 | 100 | 6,64 | 1E-17 |
| O76027 | Annexin A9 [OS=Homo sapiens] | ANXA9 | 100 | 6,64 | 1E-17 |
| Q2TAZ0-4 | Isoform 3 of Autophagy-related protein 2 homolog A [OS=Homo sapiens] | ATG2A | 100 | 6,64 | 1E-17 |

## 1.2 Table S2. KEGG pathway enrichment analysis of common genes generated using SRplot tool.

Description: Column A: Term ID.

Column B: Description, the description of the term.

Column C: GeneRatio, the number of genes enriched in certain term / the number of input genes.

Column D: BgRatio, the number of genes within certain term / the number of background genes.

Column E: pvalue, the pvalue of certain enriched term.

Column F: p.adjust, the adjusted pvalue.

Column G: qvalue, the qvalue of certain enriched term.

Column H: geneID, the gene symbols of certain enriched term overlapped with the input genes

Column I: Count, the gene counts of certain enriched term overlapped with the input genes.

Column J: Enrichment Score, A statistical measure ( -log10(p-value) ) that quantifies the significance of gene set enrichment in the term.

Column K: Fold Enrichment, the ratio of the proportion of genes in a GO term within a gene set to their proportion in the entire genome."

## A) Upregulated

| **ID** | **Description** | **GeneRatio** | **BgRatio** | **pvalue** | **p.adjust** | **qvalue** | **geneID** | **Count** | **Enrichment.Score** | **Fold.Enrichment** |
| --- | --- | --- | --- | --- | --- | --- | --- | --- | --- | --- |
| hsa04136 | Autophagy - other | 2/12 | 32/9396 | 0,000726052 | 0,019603397 | 0,01834236 | ATG2A/ATG4C | 2 | 3,139032434 | 48,9375 |
| hsa04140 | Autophagy - animal | 2/12 | 169/9396 | 0,018856343 | 0,209888591 | 0,196386985 | ATG2A/ATG4C | 2 | 1,724542538 | 9,266272189 |
| hsa04392 | Hippo signaling pathway - multiple species | 1/12 | 29/9396 | 0,036435717 | 0,209888591 | 0,196386985 | FRMD1 | 1 | 1,438472675 | 27 |
| hsa00480 | Glutathione metabolism | 1/12 | 59/9396 | 0,07284378 | 0,209888591 | 0,196386985 | GSTK1 | 1 | 1,137607524 | 13,27118644 |
| hsa00310 | Lysine degradation | 1/12 | 63/9396 | 0,077601722 | 0,209888591 | 0,196386985 | NSD2 | 1 | 1,110128642 | 12,42857143 |
| hsa00010 | Glycolysis / Gluconeogenesis | 1/12 | 67/9396 | 0,082337272 | 0,209888591 | 0,196386985 | PGK2 | 1 | 1,084403525 | 11,68656716 |
| hsa05204 | Chemical carcinogenesis - DNA adducts | 1/12 | 71/9396 | 0,087050527 | 0,209888591 | 0,196386985 | GSTK1 | 1 | 1,060228597 | 11,02816901 |
| hsa00982 | Drug metabolism - cytochrome P450 | 1/12 | 73/9396 | 0,089398823 | 0,209888591 | 0,196386985 | GSTK1 | 1 | 1,048668199 | 10,7260274 |
| hsa01230 | Biosynthesis of amino acids | 1/12 | 75/9396 | 0,091741581 | 0,209888591 | 0,196386985 | PGK2 | 1 | 1,037433779 | 10,44 |
| hsa04971 | Gastric acid secretion | 1/12 | 76/9396 | 0,092910887 | 0,209888591 | 0,196386985 | SLC26A7 | 1 | 1,031933393 | 10,30263158 |
| hsa03018 | RNA degradation | 1/12 | 78/9396 | 0,09524536 | 0,209888591 | 0,196386985 | PABPC4L | 1 | 1,021156171 | 10,03846154 |
| hsa00980 | Metabolism of xenobiotics by cytochrome P450 | 1/12 | 79/9396 | 0,096410531 | 0,209888591 | 0,196386985 | GSTK1 | 1 | 1,015875527 | 9,911392405 |
| hsa04146 | Peroxisome | 1/12 | 83/9396 | 0,10105747 | 0,209888591 | 0,196386985 | GSTK1 | 1 | 0,995431581 | 9,43373494 |
| hsa03015 | mRNA surveillance pathway | 1/12 | 103/9396 | 0,1239653 | 0,235711727 | 0,220548985 | PABPC4L | 1 | 0,906699865 | 7,601941748 |
| hsa04066 | HIF-1 signaling pathway | 1/12 | 110/9396 | 0,13185576 | 0,235711727 | 0,220548985 | PGK2 | 1 | 0,879900895 | 7,118181818 |
| hsa01200 | Carbon metabolism | 1/12 | 117/9396 | 0,139681024 | 0,235711727 | 0,220548985 | PGK2 | 1 | 0,854862591 | 6,692307692 |
| hsa05017 | Spinocerebellar ataxia | 1/12 | 144/9396 | 0,16926239 | 0,261801996 | 0,244960932 | ATG2A | 1 | 0,771439531 | 5,4375 |
| hsa04390 | Hippo signaling pathway | 1/12 | 157/9396 | 0,183170101 | 0,261801996 | 0,244960932 | FRMD1 | 1 | 0,737145414 | 4,987261146 |
| hsa04110 | Cell cycle | 1/12 | 158/9396 | 0,184231034 | 0,261801996 | 0,244960932 | ATRX | 1 | 0,73463721 | 4,955696203 |
| hsa04360 | Axon guidance | 1/12 | 184/9396 | 0,211375852 | 0,2853574 | 0,267001076 | DPYSL2 | 1 | 0,67494463 | 4,255434783 |
| hsa05202 | Transcriptional misregulation in cancer | 1/12 | 201/9396 | 0,22867401 | 0,294009441 | 0,275096553 | NSD2 | 1 | 0,640783193 | 3,895522388 |
| hsa04015 | Rap1 signaling pathway | 1/12 | 212/9396 | 0,239680855 | 0,294153776 | 0,275231604 | RGS14 | 1 | 0,620366655 | 3,693396226 |
| hsa04810 | Regulation of actin cytoskeleton | 1/12 | 232/9396 | 0,25932519 | 0,304425223 | 0,284842314 | CYRIA | 1 | 0,586155295 | 3,375 |
| hsa05016 | Huntington disease | 1/12 | 311/9396 | 0,332458044 | 0,3740153 | 0,349955836 | ATG2A | 1 | 0,478263154 | 2,517684887 |
| hsa05014 | Amyotrophic lateral sclerosis | 1/12 | 371/9396 | 0,383511583 | 0,41419251 | 0,387548547 | ATG2A | 1 | 0,416221514 | 2,110512129 |
| hsa05010 | Alzheimer disease | 1/12 | 391/9396 | 0,399717144 | 0,41509088 | 0,388389128 | ATG2A | 1 | 0,398247224 | 2,002557545 |
| hsa05022 | Pathways of neurodegeneration - multiple diseases | 1/12 | 483/9396 | 0,46935347 | 0,46935347 | 0,439161141 | ATG2A | 1 | 0,328499967 | 1,621118012 |

## B) Downregulated

| **ID** | **Description** | **GeneRatio** | **BgRatio** | **pvalue** | **p.adjust** | **qvalue** | **geneID** | **Count** | **Enrichment.Score** | **Fold.Enrichment** |
| --- | --- | --- | --- | --- | --- | --- | --- | --- | --- | --- |
| hsa04520 | Adherens junction | 2/12 | 93/9396 | 0,005996954 | 0,148485121 | 0,126993854 | MYL5/CCM2 | 2 | 2,222069317 | 16,83870968 |
| hsa05418 | Fluid shear stress and atherosclerosis | 2/12 | 142/9396 | 0,013554353 | 0,148485121 | 0,126993854 | ACVR2A/GSTM4 | 2 | 1,867921204 | 11,02816901 |
| hsa04550 | Signaling pathways regulating pluripotency of stem cells | 2/12 | 144/9396 | 0,01392048 | 0,148485121 | 0,126993854 | ACVR2A/ISL1 | 2 | 1,856345785 | 10,875 |
| hsa04136 | Autophagy - other | 1/12 | 32/9396 | 0,040134617 | 0,243044296 | 0,207866832 | ATG4D | 1 | 1,396480881 | 24,46875 |
| hsa01250 | Biosynthesis of nucleotide sugars | 1/12 | 37/9396 | 0,046270536 | 0,243044296 | 0,207866832 | NAGK | 1 | 1,334695474 | 21,16216216 |
| hsa00520 | Amino sugar and nucleotide sugar metabolism | 1/12 | 38/9396 | 0,047493396 | 0,243044296 | 0,207866832 | NAGK | 1 | 1,323366772 | 20,60526316 |
| hsa00480 | Glutathione metabolism | 1/12 | 59/9396 | 0,07284378 | 0,243044296 | 0,207866832 | GSTM4 | 1 | 1,137607524 | 13,27118644 |
| hsa00310 | Lysine degradation | 1/12 | 63/9396 | 0,077601722 | 0,243044296 | 0,207866832 | NSD2 | 1 | 1,110128642 | 12,42857143 |
| hsa05204 | Chemical carcinogenesis - DNA adducts | 1/12 | 71/9396 | 0,087050527 | 0,243044296 | 0,207866832 | GSTM4 | 1 | 1,060228597 | 11,02816901 |
| hsa00982 | Drug metabolism - cytochrome P450 | 1/12 | 73/9396 | 0,089398823 | 0,243044296 | 0,207866832 | GSTM4 | 1 | 1,048668199 | 10,7260274 |
| hsa01524 | Platinum drug resistance | 1/12 | 75/9396 | 0,091741581 | 0,243044296 | 0,207866832 | GSTM4 | 1 | 1,037433779 | 10,44 |
| hsa00980 | Metabolism of xenobiotics by cytochrome P450 | 1/12 | 79/9396 | 0,096410531 | 0,243044296 | 0,207866832 | GSTM4 | 1 | 1,015875527 | 9,911392405 |
| hsa00983 | Drug metabolism - other enzymes | 1/12 | 81/9396 | 0,098736745 | 0,243044296 | 0,207866832 | GSTM4 | 1 | 1,005521192 | 9,666666667 |
| hsa04970 | Salivary secretion | 1/12 | 97/9396 | 0,117149784 | 0,260832191 | 0,223080164 | CST3 | 1 | 0,931258508 | 8,072164948 |
| hsa04350 | TGF-beta signaling pathway | 1/12 | 110/9396 | 0,13185576 | 0,260832191 | 0,223080164 | ACVR2A | 1 | 0,879900895 | 7,118181818 |
| hsa04670 | Leukocyte transendothelial migration | 1/12 | 116/9396 | 0,138567102 | 0,260832191 | 0,223080164 | MYL5 | 1 | 0,858339867 | 6,75 |
| hsa04724 | Glutamatergic synapse | 1/12 | 116/9396 | 0,138567102 | 0,260832191 | 0,223080164 | DLGAP1 | 1 | 0,858339867 | 6,75 |
| hsa04142 | Lysosome | 1/12 | 133/9396 | 0,157325147 | 0,279689149 | 0,239207825 | GNPTAB | 1 | 0,803201855 | 5,887218045 |
| hsa04140 | Autophagy - animal | 1/12 | 169/9396 | 0,195818219 | 0,296371646 | 0,25347575 | ATG4D | 1 | 0,708146904 | 4,633136095 |
| hsa05225 | Hepatocellular carcinoma | 1/12 | 170/9396 | 0,196864082 | 0,296371646 | 0,25347575 | GSTM4 | 1 | 0,705833513 | 4,605882353 |
| hsa04360 | Axon guidance | 1/12 | 184/9396 | 0,211375852 | 0,296371646 | 0,25347575 | MYL5 | 1 | 0,67494463 | 4,255434783 |
| hsa04814 | Motor proteins | 1/12 | 197/9396 | 0,224635442 | 0,296371646 | 0,25347575 | MYL5 | 1 | 0,648521721 | 3,974619289 |
| hsa05202 | Transcriptional misregulation in cancer | 1/12 | 201/9396 | 0,22867401 | 0,296371646 | 0,25347575 | NSD2 | 1 | 0,640783193 | 3,895522388 |
| hsa04510 | Focal adhesion | 1/12 | 203/9396 | 0,230686054 | 0,296371646 | 0,25347575 | MYL5 | 1 | 0,636978659 | 3,857142857 |
| hsa05207 | Chemical carcinogenesis - receptor activation | 1/12 | 217/9396 | 0,244636209 | 0,296371646 | 0,25347575 | GSTM4 | 1 | 0,611479261 | 3,608294931 |
| hsa05208 | Chemical carcinogenesis - reactive oxygen species | 1/12 | 227/9396 | 0,254458216 | 0,296371646 | 0,25347575 | GSTM4 | 1 | 0,594383521 | 3,449339207 |
| hsa04810 | Regulation of actin cytoskeleton | 1/12 | 232/9396 | 0,25932519 | 0,296371646 | 0,25347575 | MYL5 | 1 | 0,586155295 | 3,375 |
| hsa04820 | Cytoskeleton in muscle cells | 1/12 | 232/9396 | 0,25932519 | 0,296371646 | 0,25347575 | MYL5 | 1 | 0,586155295 | 3,375 |
| hsa05132 | Salmonella infection | 1/12 | 251/9396 | 0,277555278 | 0,298080119 | 0,254936943 | MYL5 | 1 | 0,556650509 | 3,119521912 |
| hsa05131 | Shigellosis | 1/12 | 253/9396 | 0,279450111 | 0,298080119 | 0,254936943 | MYL5 | 1 | 0,553695713 | 3,09486166 |
| hsa04060 | Cytokine-cytokine receptor interaction | 1/12 | 298/9396 | 0,320897876 | 0,33124942 | 0,283305425 | ACVR2A | 1 | 0,493633158 | 2,627516779 |
| hsa04080 | Neuroactive ligand-receptor interaction | 1/12 | 370/9396 | 0,382690875 | 0,382690875 | 0,327301407 | CHRNB1 | 1 | 0,417151893 | 2,116216216 |

## 1.3 Table S3. Differentially expressed proteins uniquely regulated by each DOAC.

| A. Apixaban | | | | | |
| --- | --- | --- | --- | --- | --- |
| Overexpressed (n=12) | | | Underexpessed (n=39) | | |
| UniProt ID | Gene Symbol | Log2 Abudance ratio | UniProt ID | Gene Symbol | Log2 Abudance ratio |
| Q5JUR7 | TEX30 | 2,01 | Q96SR6-2 | ZNF382 | -1,93 |
| P49765 | VEGFB | 2,47 | Q6N043 | ZNF280D | -6,64 |
| O94827-7 | PLEKHG5 | 2,54 | Q9NYL5 | CYP39A1 | -6,64 |
| P27695 | APEX1 | 2,76 | Q9NR30 | DDX21 | -6,64 |
| Q9BZQ4 | NMNAT2 | 2,8 | P0C870 | JMJD7 | -6,64 |
| Q8TAZ6 | CMTM2 | 2,86 | Q8IZ41 | RASEF | -6,64 |
| Q9UJA3 | MCM8 | 1,9 | P42768 | WAS | -6,64 |
| P43251-3 | BTD | 1,93 | Q9H4B8 | DPEP3 | -6,64 |
| Q14CB8-4 | ARHGAP19 | 1,94 | Q9HCC6 | HES4 | -6,64 |
| Q15072 | ZNF146 | 1,94 | Q9UBN7 | HDAC6 | -6,64 |
| Q06889-2 | EGR3 | 1,97 | Q9UL46 | PSME2 | -6,64 |
| Q4G0J3 | LARP7 | 1,99 | P23588 | EIF4B | -6,64 |
|  |  |  | Q96RU2 | USP28 | -6,64 |
|  |  |  | Q86WX3 | RPS19BP1 | -6,64 |
|  |  |  | Q9NP81 | SARS2 | -6,64 |
|  |  |  | Q15181 | PPA1 | -6,64 |
|  |  |  | P12643 | BMP2 | -6,64 |
|  |  |  | O15085 | ARHGEF11 | -6,64 |
|  |  |  | Q9Y2D2 | SLC35A3 | -6,64 |
|  |  |  | Q9Y3Q8 | TSC22D4 | -6,64 |
|  |  |  | A0A2R8Y7Y5 | CIST1 | -6,64 |
|  |  |  | Q92963-2 | RIT1 | -6,64 |
|  |  |  | Q8N752 | CSNK1A1L | -6,64 |
|  |  |  | P61962 | DCAF7 | -6,64 |
|  |  |  | Q16890-5 | TPD52L1 | -6,64 |
|  |  |  | Q6SA08 | TSSK4 | -6,64 |
|  |  |  | Q5JPH6 | EARS2 | -6,64 |
|  |  |  | Q86VX9-5 | MON1A | -6,64 |
|  |  |  | Q5U5R9 | HECTD2 | -6,64 |
|  |  |  | Q96N06-2 | SPATA33 | -6,64 |
|  |  |  | Q9UFH2 | DNAH17 | -6,64 |
|  |  |  | O14782 | KIF3C | -6,64 |
|  |  |  | Q9BRD0 | BUD13 | -5,29 |
|  |  |  | Q8IV38 | ANKMY2 | -3,37 |
|  |  |  | O43896 | KIF1C | -2,17 |
|  |  |  | Q13442 | PDAP1 | -2,16 |
|  |  |  | Q494R0 | FBXL19-AS1 | -2,14 |
|  |  |  | Q5HYI8 | RABL3 | -2,09 |
|  |  |  | Q6ZQQ6 | WDR87 | -2,04 |

| B. Rivaroxaban | | | | | | | | | | | | | | | |  |  |
| --- | --- | --- | --- | --- | --- | --- | --- | --- | --- | --- | --- | --- | --- | --- | --- | --- | --- |
| Overexpressed (n=16) | | | | | | | Underexpessed (n=13) | | | | | | | | |  |  |
| UniProt ID | Gene Symbol | | | Log2 Abudance ratio | | | UniProt ID | | | Gene Symbol | | | Log2 Abudance ratio | | |  |  |
| Q96JE7 | SEC16B | | | 2,06 | | | Q13405 | | | MRPL49 | | | -6,64 | | |  |  |
| P11137 | MAP2 | | | 2,06 | | | Q9H2C1 | | | LHX5 | | | -6,64 | | |  |  |
| Q8IXM2 | BACC1 | | | 2,08 | | | Q9NSK0-3 | | | KLC4 | | | -6,64 | | |  |  |
| Q86VX9-5 | MON1A | | | 2,2 | | | Q5XG92-6 | | | CES4A | | | -6,64 | | |  |  |
| Q8IXL7-2 | MSRB3 | | | 2,21 | | | Q9UJY5-3 | | | GGA1 | | | -6,64 | | |  |  |
| Q8NG35 | DEFB105A; DEFB105B | | | 2,31 | | | Q16585 | | | SGCB | | | -6,64 | | |  |  |
| Q96N06-2 | SPATA33 | | | 2,92 | | | Q8TC92-2 | | | ENOX1 | | | -6,64 | | |  |  |
| Q96B26 | EXOSC8 | | | 3,08 | | | P51172-1 | | | SCNN1D | | | -6,64 | | |  |  |
| Q9H425 | C1orf198 | | | 3,58 | | | Q5JQC9-2 | | | AKAP4 | | | -6,64 | | |  |  |
| Q9BZF9 | UACA | | | 4,63 | | | Q9UNX9 | | | KCNJ14 | | | -6,64 | | |  |  |
| Q9H0T7 | RAB17 | | | 6,64 | | | Q96Q15-3 | | | SMG1 | | | -5,31 | | |  |  |
| A0A590UK24 | LOC122319696 | | | 6,64 | | | Q719H9 | | | KCTD1 | | | -4,19 | | |  |  |
| O75533 | SF3B1 | | | 6,64 | | | P32019-3 | | | INPP5B | | | -3,31 | | |  |  |
| Q02241 | KIF23 | | | 6,64 | | |  | | |  | | |  | | |  |  |
| Q8N4G2 | ARL14 | | | 6,64 | | |  | | |  | | |  | | |  |  |
| P31641 | SLC6A6 | | | 6,64 | | |  | | |  | | |  | | |  |  |
| C. **Edoxaban** | | | | | | | | | | | | | | | | |  |
| Overexpressed (n=11) | | | | | | | | Underexpessed (n=23) | | | | | | | | |  |
| UniProt ID | | Gene Symbol | | | Log2 Abudance ratio | | | UniProt ID | | | Gene Symbol | | | Log2 Abudance ratio | | |  |
| Q9H0B3 | | IQCN | | | 2,13 | | | Q05682-3 | | | CALD1 | | | -6,64 | | |  |
| Q16585 | | SGCB | | | 2,13 | | | A6NEH8 | | | ZNF503-AS2 | | | -6,64 | | |  |
| Q8IWB1 | | ITPRIP | | | 2,25 | | | Q13439 | | | GOLGA4 | | | -6,64 | | |  |
| Q9UBL6 | | CPNE7 | | | 2,29 | | | Q14978 | | | NOLC1 | | | -6,64 | | |  |
| Q9H063 | | MAF1 | | | 2,38 | | | Q5VZF2 | | | MBNL2 | | | -6,64 | | |  |
| Q9P2N2-3 | | ARHGAP28 | | | 2,43 | | | Q96MU7 | | | YTHDC1 | | | -6,64 | | |  |
| P51161 | | FABP6 | | | 2,45 | | | Q5VV43 | | | KIAA0319 | | | -6,64 | | |  |
| Q8N729 | | NPW | | | 2,63 | | | Q9UK13 | | | ZNF221 | | | -4,43 | | |  |
| P19429 | | TNNI3 | | | 2,73 | | | Q96B86-4 | | | RGMA | | | -4,18 | | |  |
| Q5VWJ9 | | SNX30 | | | 3,02 | | | Q6UWS5 | | | PET117 | | | -3,18 | | |  |
| Q8N7X4 | | MAGEB6 | | | 6,64 | | | Q96HW7 | | | INTS4 | | | -2,9 | | |  |
|  | |  | | |  | | | A6NML5 | | | TMEM212 | | | -2,76 | | |  |
|  | |  | | |  | | | P31271 | | | HOXA13 | | | -2,51 | | |  |
|  | |  | | |  | | | Q7Z3Z2 | | | RD3 | | | -2,41 | | |  |
|  | |  | | |  | | | O14904 | | | WNT9A | | | -2,39 | | |  |
|  | |  | | |  | | | Q8NCF5-3 | | | NFATC2IP | | | -2,29 | | |  |
|  | |  | | |  | | | Q14117 | | | DPYS | | | -2,28 | | |  |
|  | |  | | |  | | | Q16778 | | | H2BC21 | | | -2,14 | | |  |
|  | |  | | |  | | | Q6UXD7 | | | SLC49A3 | | | -2,07 | | |  |
|  | |  | | |  | | | Q6P1J6-2 | | | PLB1 | | | -2,07 | | |  |
|  | |  | | |  | | | Q15717-2 | | | ELAVL1 | | | -1,84 | | |  |
|  | |  | | |  | | | Q8N9Z0 | | | ZNF610 | | | -1,8 | | |  |
|  | |  | | |  | | | Q9NPJ4 | | | PNRC2 | | | -1,8 | | |  |
| D. **Dabigatran** | | | | | | | | | | | | | | | | | |
| Overexpressed (n=8) | | | | | | | | | Underexpessed (n=25) | | | | | | | | |
| UniProt ID | | | Gene Symbol | | | Log2 Abudance ratio | | | UniProt ID | | | Gene Symbol | | | Log2 Abudance ratio | | |
| Q53RT3 | | | ASPRV1 | | | 2,09 | | | P29372 | | | MPG | | | -6,64 | | |
| P17025 | | | ZNF182 | | | 2,26 | | | Q4G0J3 | | | LARP7 | | | -6,64 | | |
| Q9H6U6 | | | BCAS3 | | | 2,4 | | | Q8N2W9 | | | PIAS4 | | | -6,64 | | |
| Q8NA61-2 | | | CBY2 | | | 2,41 | | | Q16831 | | | UPP1 | | | -6,64 | | |
| O14530 | | | TXNDC9 | | | 2,93 | | | P56545 | | | CTBP2 | | | -6,64 | | |
| Q8N584-2 | | | TTC39C | | | 3,69 | | | Q7Z7A1-2 | | | CNTRL | | | -6,64 | | |
| Q155Q3-2 | | | DIXDC1 | | | 3,96 | | | Q3ZCX4-2 | | | ZNF568 | | | -6,64 | | |
| Q14524 | | | SCN5A | | | 6,64 | | | Q9C0C2-2 | | | TNKS1BP1 | | | -3,94 | | |
|  | | |  | | |  | | | Q9NR64 | | | KLHL1 | | | -3,21 | | |
|  | | |  | | |  | | | Q9H6X5 | | | C19orf44 | | | -3,21 | | |
|  | | |  | | |  | | | P09564 | | | CD7 | | | -3,19 | | |
|  | | |  | | |  | | | Q06889-2 | | | EGR3 | | | -2,93 | | |
|  | | |  | | |  | | | Q9H503 | | | BANF2 | | | -2,9 | | |
|  | | |  | | |  | | | Q6ZU67-3 | | | BEND4 | | | -2,86 | | |
|  | | |  | | |  | | | Q658Y4 | | | FAM91A1 | | | -2,44 | | |
|  | | |  | | |  | | | P01344 | | | IGF2 | | | -2,39 | | |
|  | | |  | | |  | | | Q06730-3 | | | ZNF33A | | | -2,38 | | |
|  | | |  | | |  | | | Q9Y3T9 | | | NOC2L | | | -2,32 | | |
|  | | |  | | |  | | | Q02413-2 | | | DSG1 | | | -2,31 | | |
|  | | |  | | |  | | | Q99598 | | | TSNAX | | | -2,25 | | |
|  | | |  | | |  | | | P82933 | | | MRPS9 | | | -2,23 | | |
|  | | |  | | |  | | | Q9H310 | | | RHBG | | | -2,18 | | |
|  | | |  | | |  | | | P26006 | | | ITGA3 | | | -2,16 | | |
|  | | |  | | |  | | | Q9BZE4-3 | | | GTPBP4 | | | -2,11 | | |
|  | | |  | | |  | | | Q9NYK5 | | | MRPL39 | | | -2,07 | | |

## 1.4 Table S4: Representative LC–MS injection sequence.

Representative sample sequence showing grouped injection of the experimental conditions, with three consecutive technical replicate injections for each sample followed by an intermediate blank injection. Blank injections were included to monitor potential peptide carryover between sample groups.

| **Injection order** | **RAW file name (example)** | **Sample ID (your label)** | **Treatment** | **Experiment** | **Biological replicate** | **Injection type** | **Notes** |
| --- | --- | --- | --- | --- | --- | --- | --- |
| 1 | proteomics 80min first_run_of_the_day_blank.raw | Blank_1 | Blank | – | – | System blank | Start-of-day system blank |
| 2 | proteomics 80min [Sample_1].raw | Sample_1 | [e.g. Apix] | 1 | 1 | Biological sample | First biological injection |
| 3 | proteomics 80min [Sample_2].raw | Sample_2 | [e.g. Apix] | 1 | 1 | Biological sample |  |
| 4 | proteomics 80min [Sample_3].raw | Sample_3 | [e.g. Apix] | 1 | 1 | Biological sample |  |
| 5 | proteomics 80min intermediate_blank1.raw | Blank_2 | Blank | – | – | Intermediate blank | After first sample block |
| 6 | proteomics 80min [Sample_4].raw | Sample_4 | [e.g. Riva] | 1 | 2 | Biological sample |  |
| 7 | proteomics 80min [Sample_5].raw | Sample_5 | [e.g. Riva] | 1 | 2 | Biological sample |  |
| 8 | proteomics 80min [Sample_6].raw | Sample_6 | [e.g. Riva] | 1 | 2 | Biological sample |  |
| 9 | proteomics 80min [Sample_7].raw | Sample_7 | [e.g. Edox] | 1 | 3 | Biological sample |  |
| 10 | proteomics 80min [Sample_8].raw | Sample_8 | [e.g. Edox] | 1 | 3 | Biological sample |  |
| 11 | proteomics 80min [Sample_9].raw | Sample_9 | [e.g. Edox] | 1 | 3 | Biological sample |  |
| 12 | proteomics 80min intermediate_blank2.raw | Blank_3 | Blank | – | – | Intermediate blank |  |
| 13 | proteomics 80min [Sample_10].raw | Sample_10 | [e.g. Dabi] | 1 | 1 | Biological sample |  |
| 14 | proteomics 80min [Sample_11].raw | Sample_11 | [e.g. Dabi] | 1 | 1 | Biological sample |  |
| 15 | proteomics 80min [Sample_12].raw | Sample_12 | [e.g. Dabi] | 1 | 1 | Biological sample |  |
| 16 | proteomics 80min [Sample_13].raw | Sample_13 | [e.g. Control] | 1 | 1 | Biological sample |  |
| 17 | proteomics 80min [Sample_14].raw | Sample_14 | [e.g. Control] | 1 | 1 | Biological sample |  |
| 18 | proteomics 80min [Sample_15].raw | Sample_15 | [e.g. Control] | 1 | 1 | Biological sample |  |
| 19 | proteomics 80min intermediate_blank3.raw | Blank_4 | Blank | – | – | Intermediate blank |  |
| 20 | proteomics 80min [Sample_16].raw | Sample_16 | [e.g. Apix] | 2 | 1 | Biological sample | Second experiment block |
| 21 | proteomics 80min [Sample_17].raw | Sample_17 | [e.g. Apix] | 2 | 1 | Biological sample |  |
| 22 | proteomics 80min [Sample_18].raw | Sample_18 | [e.g. Apix] | 2 | 1 | Biological sample |  |
| 23 | proteomics 80min [Sample_19].raw | Sample_19 | [e.g. Riva] | 2 | 2 | Biological sample |  |
| 24 | proteomics 80min [Sample_20].raw | Sample_20 | [e.g. Riva] | 2 | 2 | Biological sample |  |
| 25 | proteomics 80min [Sample_21].raw | Sample_21 | [e.g. Riva] | 2 | 2 | Biological sample |  |
| 26 | proteomics 80min intermediate_blank4.raw | Blank_5 | Blank | – | – | Intermediate blank |  |
| 27 | proteomics 80min [Sample_22].raw | Sample_22 | [e.g. Edox] | 2 | 3 | Biological sample |  |
| 28 | proteomics 80min [Sample_23].raw | Sample_23 | [e.g. Edox] | 2 | 3 | Biological sample |  |
| 29 | proteomics 80min [Sample_24].raw | Sample_24 | [e.g. Edox] | 2 | 3 | Biological sample |  |
| 30 | proteomics 80min [Sample_25].raw | Sample_25 | [e.g. Dabi] | 2 | 1 | Biological sample |  |
| 31 | proteomics 80min [Sample_26].raw | Sample_26 | [e.g. Dabi] | 2 | 1 | Biological sample |  |
| 32 | proteomics 80min [Sample_27].raw | Sample_27 | [e.g. Dabi] | 2 | 1 | Biological sample |  |
| 33 | proteomics 80min intermediate_blank5.raw | Blank_6 | Blank | – | – | Intermediate blank |  |
| 34 | proteomics 80min [Sample_28].raw | Sample_28 | [e.g. Control] | 2 | 2 | Biological sample |  |
| 35 | proteomics 80min [Sample_29].raw | Sample_29 | [e.g. Control] | 2 | 2 | Biological sample |  |
| 36 | proteomics 80min [Sample_30].raw | Sample_30 | [e.g. Control] | 2 | 2 | Biological sample |  |
| … | … | … | … | … | … | … | … |
| Last – 3 | proteomics 80min intermediate_blank9.raw | Blank_10 | Blank | – | – | Intermediate blank | Final intermediate blank |
| Last – 2 | proteomics 80min washing method 7 seesaw.raw | Wash_1 | Wash | – | – | Column wash | End-of-sequence wash |
| Last – 1 | endsequence_seesaw_wash.raw | Wash_2 | Wash | – | – | Column wash |  |
| Last | easy spray shut down method.raw | Shutdown | Shutdown | – | – | System shutdown |  |
